# Functional Environmental Screening of a Metagenomic Library Identifies *stlA*; A Unique Salt Tolerance Locus from the Human Gut Microbiome 

**DOI:** 10.1371/journal.pone.0082985

**Published:** 2013-12-12

**Authors:** Eamonn P. Culligan, Roy D. Sleator, Julian R. Marchesi, Colin Hill

**Affiliations:** 1 Alimentary Pharmabiotic Centre, University College Cork, Cork, Ireland; 2 School of Microbiology, University College Cork, Cork, Ireland; 3 Department of Biological Sciences, Cork Institute of Technology, Cork, Ireland; 4 Cardiff School of Biosciences, Cardiff University, Cardiff, United Kingdom; 5 Department of Hepatology and Gastroenterology, Imperial College London, London, United Kingdom; Wilfrid Laurier University, Canada

## Abstract

Functional environmental screening of metagenomic libraries is a powerful means to identify and assign function to novel genes and their encoded proteins without any prior sequence knowledge. In the current study we describe the identification and subsequent analysis of a salt-tolerant clone from a human gut metagenomic library. Following transposon mutagenesis we identified an unknown gene (*stlA*, for “salt tolerance locus A”) with no current known homologues in the databases. Subsequent cloning and expression in *Escherichia coli* MKH13 revealed that *stlA* confers a salt tolerance phenotype in its surrogate host. Furthermore, a detailed *in silico* analysis was also conducted to gain additional information on the properties of the encoded StlA protein. The *stlA* gene is rare when searched against human metagenome datasets such as MetaHit and the Human Microbiome Project and represents a novel and unique salt tolerance determinant which appears to be found exclusively in the human gut environment.

## Introduction

The human gastrointestinal (GI) tract is home to hundreds of bacterial species [1] which play an important and complex role in host health, metabolism and physiology [2]. This relatively diverse community is dominated by two bacterial phyla; the *Bacteroidetes* and *Firmicutes*, with most of the remaining microbes represented by members of the *Actinobacteria*, *Proteobacteria*, *Verrucomicrobia*, and *Fusobacteria* [3]. A significant proportion (estimates range from approximately 50-80%) of this bacterial community has thus far proved recalcitrant to traditional laboratory culture [3,4], although that number is constantly decreasing [5-7]. The emergence of culture-independent techniques such as metagenomics in the past 10-15 years has enabled researchers to study these “unculturable organisms” (although “as-yet uncultured” would be more accurate) through direct sequencing of metagenomic DNA or through cloning and functional expression in a heterologous host - an approach referred to as functional genomics [8,9].

The human GI tract imposes numerous stresses on its resident and transient microbiota [10]. The ability to adapt to and resist conditions such as low pH, bile acids, elevated osmolarity, nutrient limitation, host immune factors and competing microorganisms is a determining factor in niche colonisation and proliferation [11]. Our research focuses specifically on the osmotic stress response. Bacteria generally elicit a phased response when challenged in such a manner, firstly by the rapid accumulation of potassium (K^+^) ions (primary response), followed by the synthesis or accumulation of osmoprotectant compounds (secondary response) [12-15]. A third mechanism is also employed which can involve a broad range of genes that are seemingly unrelated to the primary and secondary responses [16-19]. These atypical, ancillary systems are arguably more interesting and provide a more complete view of the cellular response to osmotic stress in different bacteria and may also identify specific strategies employed by specific bacteria in distinct environments. 

Our aim was to identify novel genes encoding proteins that could confer a salt tolerance phenotype. It is hoped that the identification of atypical genes, which have not previously been linked to salt tolerance will help to broaden our understanding and possibly lead to the identification of novel and unusual systems that play as yet undefined roles in salt tolerance. While sequenced-based metagenomics can define the abundance and diversity of different bacteria within a given microbiome, it cannot enable researchers to assign novel functions to new or known genes. This task can only be achieved through functional screening of metagenomic libraries using activity-based assays. Approximately 30-40% of genes in a given genome will be annotated as hypothetical, conserved hypothetical or function unknown [20], while ~75% of functions important for life in the gut consist of uncharacterized orthologous groups and/or completely novel gene families [1], emphasising the significant degree of novelty that exists in these (meta)genomes. 

A previous study from our group identified five genes (which were previously annotated) from the human gut microbiome, to which a novel function of salt tolerance could be assigned [21]. In the current study, we describe the identification of a gene with no currently known homologues. Bioinformatic analysis suggests that the gene encodes a putative membrane protein, while transposon mutagenesis and subsequent cloning and heterologous expression of the gene revealed that it conferred a salt tolerance phenotype in *Escherichia coli*. This study illustrates the power of functional environmental screening of metagenomic libraries as means to identify and assign a function to as yet unknown genes and their encoded proteins.

## Materials and Methods

### Bacterial strains and growth conditions

Bacterial strains and plasmids used in this study are listed in Table S1, while primers (Eurofins, MWG Operon, Germany) used are listed in Table S2. *E. coli* EPI300::pCC1FOS (Epicentre Biotechnologies, Madison, WI, USA) was grown in Luria-Bertani (LB) medium containing 12.5μg/ml chloramphenicol (Cm) and in 12.5μg/ml chloramphenicol plus 50μg/ml kanamycin (Kan) following EZ-Tn*5* transposon mutagenesis reactions *E. coli* MKH13 [22] and *Lactococcus lactis* MG1363 [23] were grown in LB and M17 (+0.5% glucose; GM17 media) media respectively. Media was supplemented with 20µg/ml Cm for strains transformed with the plasmid pCI372 [24]. Media was supplemented with 1.5% (w/v) agar when required. Overnight cultures of *E. coli* were grown at 37°C with shaking, while *L. lactis* cultures were grown statically at 30°C.

### Construction and screening of metagenomic library

A previously constructed fosmid clone library [25,26], created from metagenomic DNA isolated from a faecal sample from a healthy 26 year old Caucasian male was used to screen for salt-tolerant clones. The library was screened as outlined previously [21]. Briefly, a total of 23,040 clones from the library were screened on LB agar supplemented with 6.5% (w/v) NaCl and 12.5μg/ml chloramphenicol using a Genetix QPix 2 XT™ colony picking/gridding robotics platform. Plates were incubated at 37°C for 2-3 days and checked periodically for growth of likely salt-tolerant clones.

### Transposon mutagenesis

Transposon mutagenesis was carried out in accordance with the manufacturer’s instructions, using the EZ-Tn*5* <*oriV*/ KAN-2> *in vitro* transposition kit (Epicentre Biotechnologies). *E. coli* EPI300 cells were transformed with the transposon reaction mixture and selected on plates containing Cm and Kan (12.5 and 50µg/ml, respectively). The transposon insertion clones were subsequently replica plated onto LB with and without 6.5% added NaCl. Clones which grew on LB but not on LB + 6.5% NaCl suggested a likely insertion event in a gene involved in salt tolerance. Presumptive salt-tolerant knock-outs were grown overnight and a fosmid DNA extraction was performed. The extracted fosmid containing metagenomic DNA was subjected to sequencing from the ends of the transposon using the primers EZ-Tn FP-1 and EZ-Tn RP-1 (Table S2.). All sequencing was performed by GATC Biotech (Konstanz, Germany). 

### DNA manipulations

Induction of fosmids from low to high copy number for downstream applications such as transposon mutagenesis and sequencing was performed as per manufacturer’s instructions and as described previously [21]. The Qiagen QIAprep® Spin mini-prep kit was used to extract fosmids as per manufacturer’s instructions. PCR products were purified with a Qiagen PCR purification kit and digested with restriction enzymes XbaI and PstI (Roche Applied Science), followed by ligation using the Fast-Link DNA ligase kit (Epicentre Biotechnologies) to similarly digested plasmid pCI372. Electro-competent *E. coli* MKH13 and *L. lactis* MG1363 were transformed with the ligation mixture and plated on LB and GM17 agar respectively, containing 20µg/ml Cm for selection. Colony PCR was performed on resistant transformants using a primer on the *stlA* gene (*stlA* FP) and a primer on the plasmid (pCI372 RP) to confirm the presence and size of the insert.

Detection of the *stlA* gene in metagenomic DNA isolated from human stool samples was attempted using PCR. Twenty samples from the ELDERMET study [27]; which consisted of five community (healthy), five long stay (frail) old subjects and five healthy young and five young subjects with irritable bowel syndrome (IBS) were used as template DNA. Furthermore, five samples from healthy adults from a separate study [28] were also tested using the following primer pairs: *stlA* FP and *stlA* RP, *stlA*-J FP and *stlA*-J RP, *stlA*-OUT FP and *stlA*-OUT RP, *stlA*-IN FP and *stlA*-IN RP (see Table S2).

### Growth experiments

Cultures were grown overnight in appropriate media. Cells were subsequently harvested, washed in one quarter strength sterile Ringer’s solution and re-suspended in fresh broth. A 2% inoculum was sub-cultured in fresh broth containing the appropriate stress (i.e. sodium chloride (NaCl), potassium chloride (KCl), sucrose, glycerol, low pH or bile as required) and 200µl was transferred to individual wells of a sterile 96-well micro-titre plate (Sarstedt Inc. Newton, USA). Plates were incubated at 37°C (or 30° for *L. lactis* strains) for 24-48 hours in an automated spectrophotometer (Tecan Genios) which recorded the optical density at 595 nanometres (OD_595nm_) every hour. For experiments using bile, uninoculated media containing bile were dispensed as blanks in the 96-well plate and their OD_595nm_ values were subtracted from the corresponding inoculated wells to give the OD_595nm_ for the microbial fraction. The data was subsequently retrieved and analysed using the Magellan 3 software program. Representative graphs were created using the Sigma Plot 10.0 software programme (Systat Software Inc, London, UK). Results are presented as the average of triplicate experiments, with error bars being representative of the standard error of the mean (SEM). 

### BIOLOG Phenotype Microarray (PM) Assay

The phenotype microarray (PM) osmolytes microplate (PM9) was used to compare the cellular phenotypes [29] of *E. coli* MKH13::pCI372 and MKH13::pCI372-*stlA* under 96 different conditions. The BIOLOG PM protocol for *E. coli* and other Gram-negative bacteria was followed for preparation of the different inoculating fluid (IF) solutions (IF-0 and IF-10; supplied by BIOLOG) and inoculation of the PM plates. Briefly, isolated colonies were added to IF-0 fluid until a cell suspension of 42% T (transmittance) was achieved. This was subsequently diluted in IF-0 + dye mix A to achieve 85% T. Finally, this was diluted in IF-10 + dye mix A and 100ul was inoculated to each well of the PM 9 microplates. Plates were incubated at 37°C and readings were taken over a 24 hour period using an automated plate reader (BIOTEK Synergy 2) which measured the absorbance at 590nm. 

### Sequencing and bioinformatic analysis

The fosmid insert from clone SMG 25 was fully sequenced and assembled by GATC Biotech (Konstanz, Germany) using the GS FLX (Roche) pyrosequencing platform on a titanium mini-run. Putative open reading frames were predicted using Softberry FGENESB bacterial operon and gene prediction software (available at www.softberry.com). Retrieved nucleotide and translated amino acid sequences were functionally annotated by homology searches using the Basic Local Alignment and Search Tool (BLAST) using a maximum e-value cut-off of 1e^-03^, to identify homologous sequences from the National Centre for Biotechnology Information (NCBI) website: http://www.ncbi.nlm.nih.gov/blast/Blast.cgi. A list of proteins encoded on SMG 25 fosmid insert is presented in Table 1.

**Table 1:** List of putative proteins encoded on SMG 25 fosmid insert.

**Protein**	**Length (aa)**	**Highest similarity (BLASTP)**	**Highest similarity organism (BLASTP)**	**E-value**	**% coverage**	**% ID (Length of similarity, aa)**	**Putative conserved domain(s)**
1	555	Serine/ threonine protein kinase	*Akkermansia* *sp.* CAG:344	7.00E-53	50%	44% (285)	None
2	108	Hypothetical protein (Amuc_1368)	*Akkermansia muciniphila* ATCC BAA-835	1.00E-04	86%	34% (98)	None
**3**	**84**	**No significant similarity found**	**n/a**	**n/a**	**n/a**	**n/a**	**n/a**
**4**	**160**	**No significant similarity found**	**n/a**	**n/a**	**n/a**	**n/a**	**n/a**
**5**	**135**	**No significant similarity found**	**n/a**	**n/a**	**n/a**	**n/a**	**n/a**
6*	**257**	**No significant similarity found**	**n/a**	**n/a**	**n/a**	**n/a**	**n/a**
**7**	**157**	**No significant similarity found**	**n/a**	**n/a**	**n/a**	**n/a**	**DnaJ zinc-finger**
8	201	Hypothetical protein O71_18246	*Pontibacter* *sp.* BAB1700	2.00E-06	36%	39% (77)	DUF4339
9	153	Hypothetical protein HALAR_0188	Halophilic archaeon DL31	1.00E-04	53%	36% (83)	TM2
10	320	Ankyrin repeat protein	*Synergistetes bacterium SGP1*	2.00E-47	88%	45% (291)	Ankyrin repeat
11	73	Uncharacterized protein BN502_01474	*Akkermansia muciniphila* CAG:154	3.00E-04	54%	50% (40)	None
**12**	**338**	**No significant similarity found**	**n/a**	**n/a**	**n/a**	**n/a**	**n/a**
13	283	Uncharacterised protein BN502_01467	*Akkermansia muciniphila* CAG:154	0.00+00	100%	94% (283)	DUF932
14	99	Uncharacterized protein BN502_01466	*Akkermansia muciniphila* CAG:154	1.00E-59	100%	94% (99)	None
15	52	Uncharacterized protein BN502_01465	*Akkermansia muciniphila* CAG:154	2.00E-22	100%	98% (52)	None
16	160	Uncharacterized protein BN502_01464	*Akkermansia muciniphila* CAG:154	2.00E-99	100%	91% (160)	None
17	43	Uncharacterized protein BN502_01463	*Akkermansia muciniphila* CAG:154	4.00E-04	95%	90% (41)	None
18	317	Hypothetical protein (Amuc_1352 )	*Akkermansia muciniphila* ATCC BAA-835	9.00E-08	31%	40% (101)	None
**19**	**79**	**No significant similarity found**	**n/a**	**n/a**	**n/a**	**n/a**	**n/a**
20	159	Phage-associated protein	*Rhizobium lupini* HPC(L)	3.00E-36	100%	52% (159)	DUF4065, GepA
21	264	Hypothetical protein EC2865200_1013	*Escherichia coli* 2865200	5.00E-26	67%	45% (181)	None
22	154	Uncharacterized protein BN502_01474	*Akkermansia muciniphila* CAG:154	2.00E-31	72%	56% (114)	None
**23**	**514**	**No significant similarity found**	**n/a**	**n/a**	**n/a**	**n/a**	**n/a**
**24**	**129**	**No significant similarity found**	**n/a**	**n/a**	**n/a**	**n/a**	**Fimbrial OM usher protein**
**25**	**551**	**No significant similarity found**	**n/a**	**n/a**	**n/a**	**n/a**	**n/a**
26	52	Hypothetical protein HALA3H3_770002	*Halomonas* *sp.* A3H3	2.00E-04	73%	63% (38)	None
27	657	H(+)-transporting two-sector ATPase	*Akkermansia* *sp.* CAG:344	0.00E+00	88%	92% (584)	TrkH superfamily
28	445	MATE efflux family protein (Amuc_1131)	*Akkermansia* *sp.* CAG:344	0.00E+00	95%	87% (445)	MATE, NorM
**29**	**49**	**No significant similarity found**	**n/a**	**n/a**	**n/a**	**n/a**	**n/a**
**30**	**54**	**No significant similarity found**	**n/a**	**n/a**	**n/a**	**n/a**	**n/a**
31	278	Putative uncharacterized protein	*Akkermansia* *sp.* CAG:344	8.00E-56	100%	70% (279)	None
32	87	Putative uncharacterized protein	*Akkermansia* *sp.* CAG:344	3.00E-31	100%	83% (87)	None
33	186	Hypothetical protein (Amuc_1127)	*Akkermansia muciniphila* ATCC BAA-835	2.00E-61	81%	83% (153)	None
34	450	Dimethyladenosine transferase	*Akkermansia* *sp.* CAG:344	0.00E+00	99%	91% (448)	ksgA, NUDIX hydrolase
35	393	UDP-galactopyranose mutase	*Chthoniobacter flavus Ellin428*	7.00E-109	96%	52% (381)	GLF, NAD binding
36	329	UDP-glucose 4-epimerase	*Akkermansia muciniphila* ATCC BAA-835	0.00E+00	100%	96% (329)	UDP_G4E_1_SDR_e
37	55	Hypothetical protein (Amuc_1123)	*Akkermansia muciniphila* ATCC BAA-835	6.40E-03	85%	43% (39)	None
38	511	Hypothetical protein (Amuc_1124)	*Akkermansia muciniphila* ATCC BAA-835	0.00E+00	98%	86% (504)	Isoprenoid_C2_like
39	144	Sulphate transporter/anti-sigma factor antagonist	*Akkermansia muciniphila* ATCC BAA-835	4.00E-89	100%	90% (144)	STAS superfamily
40	453	Putative uncharacterized protein	*Akkermansia* *sp.* CAG:344	3.00E-180	99%	69% (454)	DUF2851
41	466	Glutamate decarboxylase	*Akkermansia muciniphila* CAG:154	0.00E+00	100%	91% (466)	AAT_I superfamily
42	1217	Outer membrane auto-transporter protein	*Akkermansia* *sp.* CAG:344	3.00E-96	100%	83% (1217)	Auto-transporter superfamily
43	142	Hypothetical protein (ANACAC_03730 )	*Anaerostipes caccae DSM 14662*	4.00E-55	99%	69% (141)	NAT_SF domain
44	132	GCN5-related N-acetyltransferase	*Akkermansia* *sp.* CAG:344	8.00E-56	97%	81% (129)	NAF_SF domain
45	947	DNA polymerase III, alpha subunit	*Akkermansia muciniphila* CAG:154	0.00E+00	100%	95% (938)	DNA_polymerase_III

Abbreviations and symbols: aa (amino acids); n/a (not applicable); %ID (% identity at amino acid level); DUF (Domain of Unknown Function); OM (outer membrane); Asterisk (*) indicates *stlA* gene product. Text in bold indicates that no homologues for these gene products were found following BLAST searches of NCBI database.

The following databases and tools were used to gain additional information on the StlA protein: Expasy ProtParam server, Conserved Domain Database (CDD), PROSITE motif search, SignalP 4.0, HMMER, TMHMM, HHPred, Softberry BProm promoter search (www.softberry.com), SOPMA, SWISS MODEL, iTasser and QUARK. Relevant information and results can be found in Table 2 [30-41].

**Table 2:** Bioinformatic analysis of StlA protein sequence.

**Database/ Tool used**	**Comment(s)**	**Feature(s) identified**	**Ref.**
**Expasy ProtParam**	Allows the computation of various physical and chemical parameters for a given protein stored in Swiss-Prot or TrEMBL or for a user entered sequence	Molecular weight = 28.62 kDa; Theoretical pI = 6.39	[38]
**Conserved domain database (CDD)**	Protein annotation resource that consists of a collection of well-annotated multiple sequence alignment models for ancient domains and full-length proteins.	No conserved domains were detected	[34]
**PROSITE motif search**	Consists of documentation entries describing protein domains, families and functional sites as well as associated patterns and profiles to identify them	No motifs were detected	[36]
**SignalP 3.0**	Predicts the presence and location of signal peptide cleavage sites in amino acid sequences from different organisms	Predicted signal peptide at position 1-35	[31]
**HMMER**	Searches sequence databases for homologs of protein sequences, and for making protein sequence alignments	Predicted signal peptide at position 1-35; Four predicted TM regions and one disorder region	[32]
**TMHMM**	Prediction of transmembrane (TM) helices in proteins	Predicted four TM regions	[41]
**HHPred**	Homology detection & structure prediction by HMM-HMM (Hidden Markov Model) comparison	Detected outer membrane insertion C-terminal signal, OmpP85	[37]
**BProm promoter search**	Prediction of bacterial promoters	-10 box predicted 56 base pairs upstream of ATG start codon; TCTTATCAT; -35 box predicted 77 base pairs upstream of ATG start codon; TTGGCT	www.softberry.com
**SOPMA**	Secondary structure prediction	Alpha-helix; 166/257 residues = 64.6%; Extended strand; 26/257 residues = 10.1%; Beta-turn; 18/257 residues = 7.00%; Random coil; 47/257 residues = 18.3%	[33]
**SWISS MODEL**	Automated protein structure homology-modelling server	No similar or suitable template structures found	[30]
**iTASSER**	Protein structure and function predictions. 3D models are built based on multiple threading alignments	All 5 predicted 3D models had a C-score of -3.41 or less which are below the -1.50 threshold for a high-confidence prediction of structure	[35,40]
**QUARK**	Algorithm for *ab initio* protein folding and protein structure prediction, using amino acid sequence only. Since no global template information is used in QUARK simulation, the server is suitable for proteins which are considered without homologous templates	Of the 10 predicted 3D models, the top template modelling (TM) score was 0.342 ±0.083, which is below the threshold of TM-score >0.50 for predicted correct fold	[39]

The Fold and Functional Assignment System (FFAS03) is a profile-profile and fold recognition algorithm that can detect remote homology between proteins [42]. FFAS03 searches numerous databases including non-redundant NCBI protein sequence database (NCBI nr), Global Ocean Sampling (GOS) from JCVI (J. Craig Venter Institute), PDB (Protein Data Bank), SCOP (Structural Classification of Proteins), and COG (Clusters of Orthologous Groups), as well as numerous metagenome datasets (microbial metagenome samples from the Joint Genome Institute, human gut metagenome samples from the Hattori lab, human oral microbiome database from the Forsyth institute and GOS data from JCVI and CAMERA). Furthermore, FFAS03 searches against the MetaHit (Metagenomics of the Human Intestinal Tract) dataset [1], which contains over 3 million unique gene sequences from the human gut microbiome. The StlA protein sequence was submitted to the server to identify proteins with distant homology based on FFAS profiling or homologues by BLAST and PSI-BLAST (Position-Specific Iterated BLAST) against the databases and metagenome datasets. The FFAS03 server can be found at: http://ffas.burnham.org/ffas-cgi/cgi/document.pl.

The Integrated Microbial Genomes and Metagenomes (IMG/M) [43] is a data management system for the comparative analysis of metagenome sequence data. IMG/M-HMP [44] specifically contains metagenome data from the Human Microbiome Project (HMP) [45]. It contains 748 metagenome datasets generated from sequencing samples from 17 different body sites (number of samples from each site are in brackets); anterior nares (94), keratinised gingiva (6), buccal mucosa (122), hard palate (1), retroauricular crease (20), left (2) and right retroauricular crease (5), palatine tonsils (6), saliva (5), throat (7), tongue dorsum (133), sub- (8) and supra-gingival plaques (127), mid-vagina (2), posterior fornix (60), vaginal introitus (3) and stool (147). It also provides tools for comparative analysis between hosted sequences and user supplied sequences. The StlA protein sequence was searched (maximum e-value cut-off of 1e^-50^) against all the available metagenomes (748) from the HMP as well against all bacterial (9049), archaeal (323), viral/phage (2809) and eukaryotic genome (183) sequences (both assembled and draft), as well as all sequenced plasmids (1193) and all other non-human metagenome datasets (representing >1,300 non-human samples) from diverse environments, including terrestrial, aquatic and host-associated plants and animals (File S1) stored in the database at the time of writing (search date 19/09/13). The IMG/M-HMP server can be found at: http://img.jgi.doe.gov/cgi-bin/imgm_hmp/main.cgi. PhiSpy [46] was used to identify putative prophage genes and the boundaries of the putative prophage region on SMG 25. 

### Taxonomic assignment of scaffolds

The scaffolds on which an *stlA* homologue was identified were subjected to BLASTX analysis (maximum e-value cut-off of 1e^-50^). The BLASTX results were downloaded and imported to MEGAN 4 (Metagenome Analyser 4) software program [47] for taxonomic assignment.

## Results

### Screening the metagenomic library

**Figure 1:**
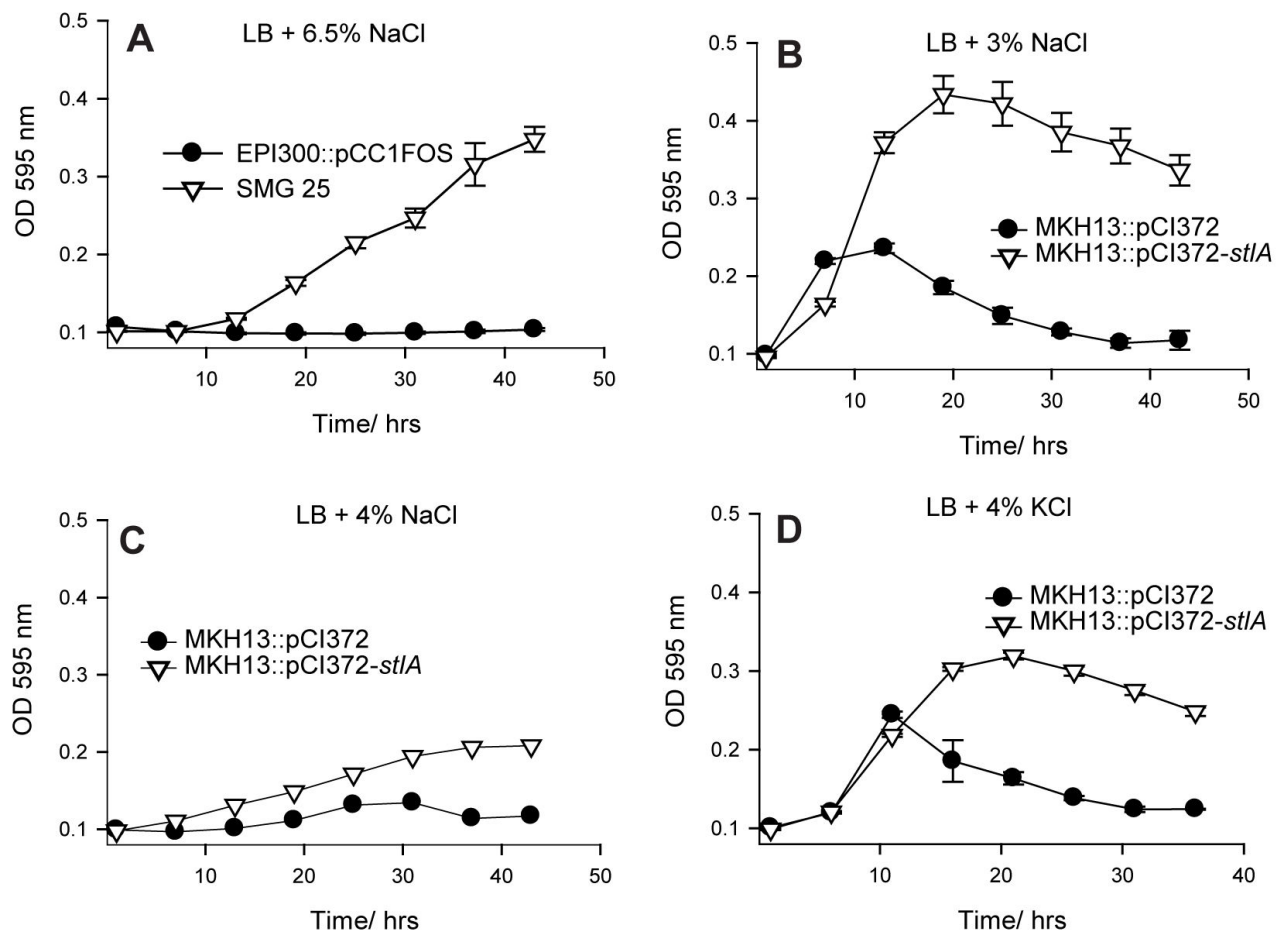
Growth experiments in NaCl and KCl. (**A**) Growth of *E. coli* EPI300::pCC1FOS and clone SMG 25 in LB broth supplemented with 6.5% sodium chloride (NaCl) (*P* <0.0001). (**B**) Growth in LB broth and LB broth supplemented with 3% NaCl (*P* =0.0470), (**C**) 4% NaCl (*P* <0.0001) and (**D**) 4% KCl (*P* <0.0001). *P*-values were determined using the student *t*-test (unpaired). Results are presented as the average of triplicate experiments, with error bars being representative of the standard error of the mean (SEM).

Screening approximately 23,000 clones from a human gut metagenomic library led to the identification of 53 clones which were designated as conferring salt-tolerance (i.e. facilitating growth on LB supplemented with 6.5% NaCl, a concentration which inhibits the growth of the cloning host carrying an empty fosmid vector). Six clones (annotated Salt MetaGenome; SMG 1-6) grew within 24 hours and the remaining 47 grew in the following 24-48 hours. SMG 25 represents one of the “late-bloomers” and was chosen at random for analysis. End sequencing of the fosmid insert revealed it shared highest genetic identity to species from the genus *Akkermansia*, namely *Akkermansia muciniphila* ATCC BAA-835*, Akkermansia muciniphila* CAG:154 and *Akkermansia* *sp.* CAG:344. *A muciniphila* ATCC BAA-835, the type strain [48,49], is a mucin degrading member of the Phylum *Verrucomicrobia* which is commonly found in the human gut microbiome [50]. Growth was monitored spectrophotometrically, by measuring the optical density at 595_nm_ (OD_595nm_). SMG 25 was shown to have a significant (unpaired student *t*-test, *P* <0.0001) growth advantage in the presence of NaCl compared to the EPI300 host strain carrying an empty fosmid vector (pCC1FOS) (Figure 1 A). 

### Fosmid sequencing and analysis

**Figure 2:**
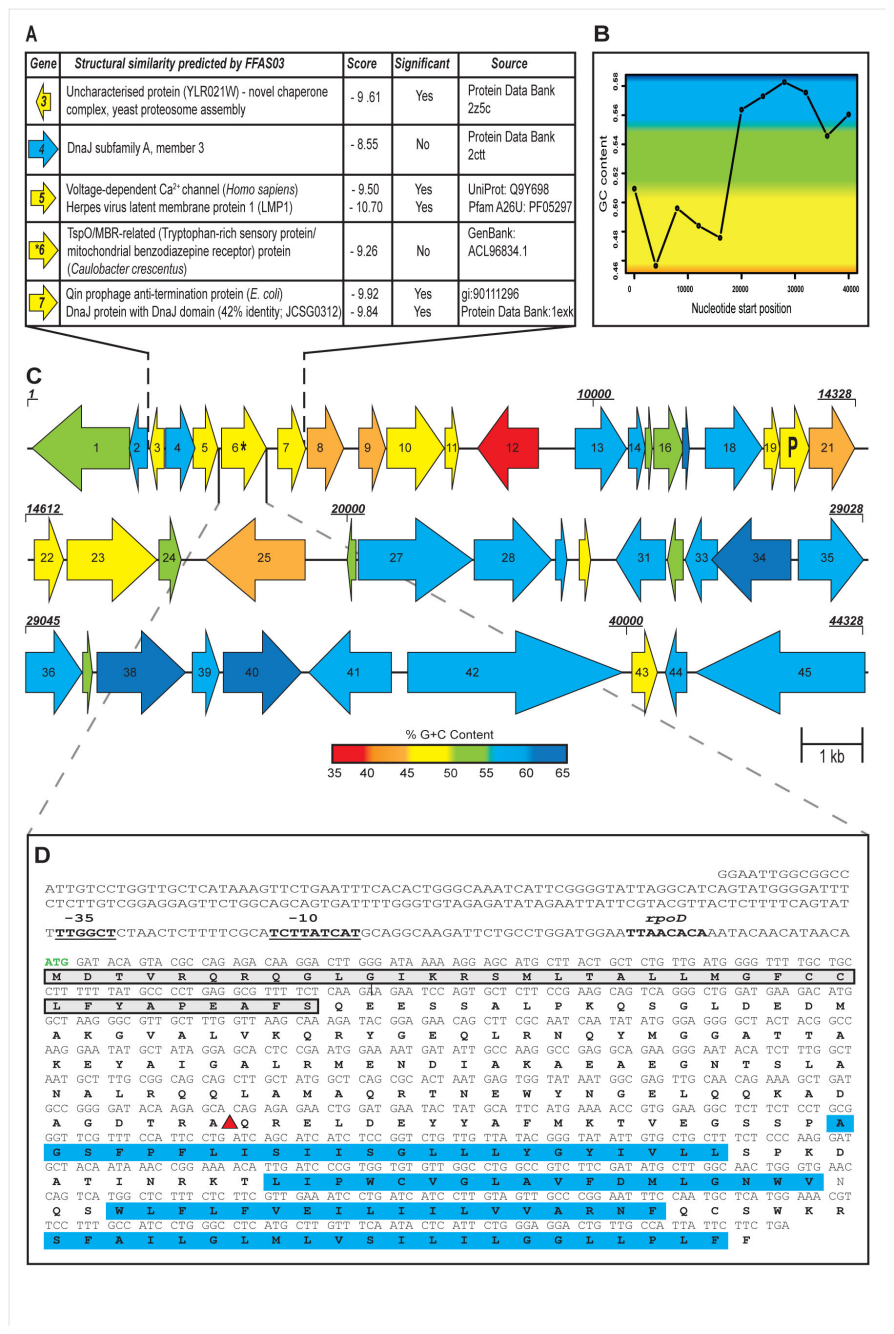
Bioinformatic analysis of SMG 25 fosmid insert. (**A**) FFAS03 analysis of the StlA protein and the encoded proteins of flanking genes was performed to identify putative distant structural homologues. A score of -9.50 or lower is considered significant. (**B**) Representation of the G+C skew of the entire fosmid insert of SMG 25. (**C**) Representation of the gene arrangement on SMG 25. Gene lengths are approximately to scale and colour coding represents G+C content of each individual gene which can be determined from the G+C content gradient bar. The presence of a phage-associated gene and clear separation in G+C content over the length of the fosmid insert indicates much of this region may have been acquired via lateral gene transfer (LGT). Phage-associated gene is marked “P”, while the *stlA* gene is indicated with an asterisk (*) symbol. Genes are numbered as indicated in Table 1 and as mentioned in the text. Numbering of some shorter genes has been excluded for clarity. Selected nucleotide positions (in base pairs) are displayed in bold italic font above genes. (**D**) A detailed view is presented of the nucleotide and amino acid sequence of the *stlA* gene and StlA protein respectively. The putative start codon is in green, while a 250 base-pair region upstream of this is shown to include putative -35 and -10 promoter regions (underlined) and a predicted *rpoD* transcription factor binding site (in bold). Amino acids surrounded by grey box indicate the predicted signal sequence of StlA and those highlighted in blue represent four transmembrane regions. The location of the EZTn*5* transposon insertion is indicated with a red triangle.

The fosmid insert from SMG 25 was fully sequenced and assembled by GATC Biotech (Konstanz, Germany) and was predicted to contain 45 putative open reading frames that encode proteins (see Table 1). Translated nucleotide sequences were subjected to BLASTP analysis to identify homologous sequences in the database. Twenty-six of the genes encoded proteins corresponding to different species of *Akkermansia* (ranging from 34-98% amino acid identity), but a sizeable proportion of the encoded proteins, approximately 27% (12/45) (highlighted in bold in Table 1) had no significant similarity to sequences in the database, indicating the presence of novel sequences. Overall, only 13 proteins could be assigned a putative function based on BLASTP searches, whilst the remaining genes encoded hypothetical or uncharacterised proteins. The full fosmid insert sequence of SMG 25 can be found in GenBank (accession number=JQ269600.1; gi=375342965). The G+C (guanine and cytosine) skew of the entire fosmid insert as well a picture of the G+C content of each individual gene are presented in Figure 2 B and C respectively. 

### Transposon (EZ-Tn*5*) mutagenesis and cloning of the *stlA* gene

Transposon mutagenesis was performed on clone SMG 25 and a transposon insertion in gene 6 was identified which eliminated the growth advantage under osmotic stress; this locus (designated *stlA*) is predicted to encode a protein of 257 amino acids which, at the time of writing, has no homologues in the database. The transposon insertion was found to be between amino acid position 136 (alanine) and 137 (glutamine) of the protein. The *stlA* gene was cloned, along with some flanking DNA that was predicted to contain the native promoter region (predicted with BProm program; see Table 2 for details), into the shuttle plasmid pCI372 and transformed into *E. coli* MKH13 and *L. lactis* MG1363.

### Growth experiments and BIOLOG phenotypic microarray

*E. coli* MKH13 cells transformed with a plasmid bearing a copy of the *stlA* gene were grown in LB broth containing various concentrations of NaCl (from 0-5% w/v added NaCl). It was observed that a statistically significant (unpaired student *t*-test) growth advantage was conferred upon the *stlA* ^+^ cells compared to wild-type MKH13 carrying an empty plasmid in LB broth supplemented with both 3% (*P* =0.0019) and 4% NaCl (*P* <0.0001) (Figure 1 B and C, respectively), while growth was similar in LB alone (data not shown).

Due to the uncharacterised and non-homologous nature of the *stlA* gene and its encoded protein, growth of wild-type MKH13 and *stlA* ^*+*^ strains was compared under 96 different conditions using BIOLOGs phenotypic microarray (PM) technology [29] to identify possible further phenotypic changes. Strains were tested on BIOLOG plate PM9, which contains different osmotic stress conditions and osmolytes for analysis. In addition to NaCl, the results indicated *stlA* ^*+*^ had an increased growth phenotype in the presence of potassium chloride (KCl). Confirmatory growth curves were performed in LB broth supplemented with a concentration of 4% KCl. A statistically significant (unpaired student *t*-test) growth advantage was observed for *stlA* ^*+*^ in LB supplemented with 4% KCl (*P* <0.0001) compared to wild-type MKH13 (Figure 1 D). 

Growth of both strains was also assessed under conditions of non-ionic osmotic stress (in the form of glycerol and sucrose), low pH and in both porcine and human bile, as all three stress conditions are commonly encountered in the GI tract. Growth of both strains was inhibited at pH 2.5 and pH 3.5, while no significant difference in growth was observed at pH 4.5 or pH 5.5, in the presence of sucrose or glycerol, or in the presence of either porcine or human bile (Figure S1).

The *stlA* genes’ ability to increase salt tolerance was also tested in a Gram positive host; *L. lactis* MG1363. There was no observable increase in salt tolerance in *L. lactis* MG1363 carrying a plasmid encoded copy of *stlA* compared to *L. lactis* carrying an empty copy of the plasmid, while a similar growth rate and final OD value was observed for both strains in GM17 broth alone (Figure S2 A and B) 

### Bioinformatic analysis of StlA

The databases and tools used to identify features of StlA are presented in Table 2 below, along with the results of the analyses. An illustration of the *stlA* gene and its associated features is presented in Figure 2 D.

### IMG/M-HMP analysis

The StlA protein sequence was screened against all available metagenomes from the human microbiome project (HMP) using BLASTP on the IMG/M-HMP website (http://img.jgi.doe.gov/cgi-bin/imgm_hmp/main.cgi) [45], which were sampled from 17 body sites giving a total of 748 samples. In addition, all available finished, permanent draft and draft genome sequences for *Bacteria*, *Archaea*, *Eukarya* and viruses/phages, as well as all available sequenced plasmids were searched using BLASTP for homologous sequences to StlA. There was no significant similarity for the StlA protein to any of the bacterial, archaeal, eukaryotic, viral or plasmid genomes/sequences, nor to any non-human associated metagenomes (over 1,300 samples from more than 200 metagenomes, see File S1). The only similarity to StlA among the sampled microbiomes was to the stool microbiome samples, where 10 similar proteins from 8 different subjects (out of 100) (Table 3) were identified on different scaffolds. The date of the last search was on 19/09/13. The taxonomic assignment of the scaffolds can be seen in Figure S3.

**Table 3:** Gene, scaffold and subject information from which *stlA* homologues were found in Human Microbiome Project (HMP) dataset.

**Stool Microbiome Subject ID (Visit number)**	**Gene ID**	**Strand**	**Start Coordinate**	**End Coordinate**	**Length** (**bp**)	**Length (aa)**	**% ID to StlA (aa)**	**Gene Product Name (*stlA* homologue)**	**Scaffold Length (bp)**	**Scaffold GC %**
**N/A**	***stlA* (from clone SMG 25; this study)**	**+**	**3193**	**3966**	**774**	**257**	**100% (257**)	**putative membrane protein**	**44331 (fosmid insert)**	**0.53**
*159753524 (2)	SRS053214_LANL_scaffold_17021__gene_42707	-	25802	26569	768	255	59% (237)	hypothetical protein	33560	0.5
*159753524 (3)	SRS077730_LANL_scaffold_24345__gene_72567	+	2793	3560	768	255	59% (237)	membrane protein	13529	0.49
**^*ǂ*^**764143897 (1)	SRS015217_WUGC_scaffold_30292__gene_65222	-	463	1236	774	257	82% (237)	membrane protein	5672	0.5
**^*ǂ*^**764143897 (2)	SRS051882_Baylor_scaffold_22757__gene_50812	-	1791	2564	774	257	82% (237)	membrane protein	7074	0.49
160643649 (1)	C2121591__gene_151559	+	1333	2157	825	274	89% (234)	membrane protein	5507	0.48
158944319 (1)	C3406971__gene_199744	-	248	1072	825	274	80% (234)	membrane protein	3122	0.52
159591683 (2)	SRS024549_LANL_scaffold_1815__gene_4559	-	4475	5248	774	257	82% (237)	membrane protein	10434	0.5
158337416 (2)	C2998990__gene_162710	+	340	972	633	211	81% (211)	hypothetical protein	974	0.45
765013792 (1)	SRS018656_WUGC_scaffold_544__gene_591	-	13762	14535	774	257	83% (237)	membrane protein	26364	0.51
159510762 (2)	SRS024075_LANL_scaffold_21370__gene_63545	-	21610	22320	711	236	82% (216)	hypothetical protein	35617	0.5

Information for *stlA* homologues found in HMP dataset, including scaffold and subject of origin. Symbols (* and ǂ) indicate detection of *stlA* homologue more than once from same subject. The StlA amino acid sequence was used to search against all 748 metagenome datasets from different body sites from the Human Microbiome Project (HMP) (1e-50 maximum e-value cut-off). Ten StlA homologues were identified in 8 different subjects. No StlA homologues were found in any other body site metagenome.

The gene neighbourhoods around the genes homologous to *stlA* on each scaffold were investigated in an attempt to gain information on possible functions and conserved gene arrangements (Figure S4). The genes most commonly flanking the stlA homologues were on the same strand of DNA and encoded an ankyrin repeat protein (COG0666), a DnaJ class molecular chaperone with C-terminal zinc-finger domain (COG0484) and a predicted membrane protein (COG2314; Pfam05154 –TM2 domain). There are also a number of hypothetical proteins, for which no additional information is currently known. On two of the larger scaffolds, genes for a restriction modification system are present, as well as an integrase/site specific recombinase protein (COG4974; Pfam00589), indicating some of this region may have been acquired by lateral gene transfer (LGT) and may represent prophage DNA. A phage-associated protein is predicted to be encoded by gene 20 (designated “P” in Figure 2 C) indicating the presence of a prophage on SMG 25 also. The fosmid insert of SMG 25 was analysed with PhiSpy [46] to identify possible prophage genes and the boundaries of the prophage region. PhiSpy predicted the prophage region to run from the start of gene 3 (nucleotide position 2024) to the end of gene 42 (nucleotide position 39972).

### FFAS03 analysis

The FFAS03 server [42] was used to detect distant homology and fold recognition to StlA. FFAS analysis was also carried out on the translated protein sequences of the neighbouring genes to *stlA* on SMG 25, which also lacked any homologues in the databases (i.e. gene 3, 4, 5 and 7; gene 6 is *stlA*). The results of FFAS03 analysis are summarised in Figure 2 A). . 

In addition to a profile-profile and a fold and functional assignment, the FFAS03 server also carries out a BLAST and PSI-BLAST search of the user sequence against numerous databases and metagenome datasets. The StlA protein was found to share significant similarity to a protein from two individuals from the MetaHit dataset [1]. These sequences corresponded to samples MH0011 (a healthy Danish female) and V1.CD-14 (a Spanish female with Crohn’s disease) which shared 60% identity (over 210 amino acids) and 82% identity (over 224 amino acids) respectively to StlA. 

### Detection of *stlA* in metagenomic DNA from human stool samples using PCR

The primer pair (*stlA* FP and *stlA* RP) initially used to amplify the *stlA* gene for cloning was unable to amplify PCR products in any of the metagenomic DNA samples (isolated from human stool microbiota), so a set of primers (*stlA*-J FP and *stlA*-J RP) were designed to amplify an internal fragment of the gene. This set of primers amplified numerous products of the correct size but these were found to be false positives following sequencing. An alignment was generated for StlA and homologous sequences from the stool microbiome from the HMP and MetaHit datasets to identify the most highly conserved regions (Figure S5) and different primer pairs were designed (*stlA*-OUT FP and RP; *stlA*-IN FP and RP). Two of the 25 metagenomic DNA samples tested (isolated from human faecal samples from ELDERMET [27] and another study [28]) generated PCR products of the correct size, which were confirmed to be *stlA* homologues following sequencing by using the *stlA*-IN FP and RP primer pair. One positive PCR product shared 72% nucleotide identity over approximately 300 base pairs (BLASTN versus stlA gene) and 64% identity (over 100 amino acids) using BLASTX, while one ELDERMET [27] sample was positive (community care/ healthy old) and confirmed by sequencing (87% identity over 339 nucleotides and 85% identity over 112 amino acids).

## Discussion

Functional screening of metagenomic libraries has the power to reveal novel functions for known genes or to identify completely novel genes and proteins. In the present study we describe the identification of an unknown protein (annotated StlA) from the human gut microbiome, which lacks any current homologues in the databases. The encoding gene (*stlA*), when expressed in *E. coli*, conferred a salt tolerance phenotype and may represent a novel stress resistance gene found exclusively among the human gut microbiota. This builds on previous work by our group, where we identified a novel function (i.e. increased salt tolerance) for five previously annotated genes (*galE*, *mazG* and *murB*) when expressed in *E. coli* [21]. 

Sequencing of the full fosmid insert from SMG 25 revealed an interesting gene landscape (Table 1), with approximately 58% of the predicted genes encoding proteins which shared highest genetic identity to different species of *Akkermansia* and 27% having no homologues in the databases. The *Akkermansia*-associated proteins and the “unknown” proteins are interspersed with proteins associated with different phyla such as *Bacteroidetes/Chlorobi* group, *Synergistetes*, *Proteobacteria*, *Chlamydiae/ Verrucomicrobia* group and *Firmicutes*, as well as *Archaea*. The percentage identity at the amino acid level ranges from 36-69%, revealing a diverse range of proteins encoded within approximately 44kb of fosmid insert DNA (Table 1). 

The G+C content of the entire fosmid insert is 52.97%, which is close to the average G+C content (55.8%) of the *A. muciniphila* genome [49]. The region from position 2024 (gene 3) to position 20148 (gene 26), which mainly consists of unknown genes or non-*Akkermansia*-associated genes has a lower G+C content of 47.97%. The region of the fosmid containing mainly *Akkermansia*-associated genes (from gene 27 at position 20120 to the end of the fosmid) has a G+C content of 56.73%, in line with the *A. muciniphila* genome (55.8%). A putative prophage region was predicted (using PhiSpy) to be present on SMG 25, running from gene 3 to 42 inclusive. It is difficult to say how reliable this prediction is because the criteria used by PhiSpy to predict prophage genes are strongly assisted by the degree of relatedness of the PhiSPy training genome sets and the genome/ DNA of the query organism [46]. Unfortunately PhiSPy does not contain an *Akkermansia* or Verrucomicrobial training genome, which would increase the predictive value of the result. However, by looking at the G+C skew of SMG 25 and the G+C content of each individual gene on SMG 25 (Figure 2 B and C, respectively), it seems the prophage could indeed begin at gene 3, but it is possible that it ends somewhere between gene 23 and 26, as there is a clear difference in G+C content visible between this region and from gene 27 to 45 at the 3’-end of the fosmid (Figure 2, B and C). Taken together these data suggest that much of this region may have been acquired through LGT. 

StlA is predicted to be a 257 amino acid, 28.62kDa membrane protein with four transmembrane regions. No conserved domains or motifs were detected, indicating the novelty of the protein. A signal peptide and a C-terminal outer membrane insertion signal are predicted to be present, suggesting that StlA may be exported to and inserted in the outer membrane. Furthermore, StlA possesses C-terminal phenylalanine residues, which are characteristic and highly conserved in outer membrane proteins [51]. A detailed illustration of these features is presented in Figure 2 D, along with putative promoter and transcription binding sites. The outer membrane itself is an important mediator to external stresses, serving as a permeability barrier and protecting the cell from compounds in the environment, while outer membrane proteins, specifically porins, play an significant role in the cellular responses to salt and osmotic stress [15,52,53]. It is noteworthy, given the likely location in the outer membrane, that StlA did not confer a salt tolerance phenotype on a Gram-positive host (*L. lactis*) (Figure S2).

Predictive 3D modelling was carried out with SWISS MODEL [30] and iTasser [35,40]. However, the results were not statistically significant, most likely due to the lack of any suitable template structure in the databases to build an appropriate model. *Ab initio* structure prediction was attempted using QUARK [39] as no template information is required and is thus suitable for proteins with no homologues. Again the results were not significant, but this is most likely due to the inherent difficulty and current limited ability of *ab initio* prediction. Successful cases of *ab initio* prediction have been limited to proteins of 100 residues or less and the fact remains that there are really no methods to predictively fold proteins of >200 amino acids without template modelling at present [39].

As no sequence-based homology for StlA could be determined with BLAST analysis, a more sensitive profile-profile comparison with FFAS03 [42] was used to detect remote homology through fold and structure recognition, as proteins with a similar structure or fold can have a common function in the absence of any sequence similarity. The highest score for StlA corresponded to a hypothetical protein from *C. crescentus*, which has a TspO/MBR domain. Members of this group are involved in transmembrane signalling and are located in the outer membrane [54,55]. They are associated with the major outer membrane porins (in prokaryotes) and with the voltage-dependent anion channel (in mitochondria), which links with the earlier observation that StlA may be inserted in the outer membrane. Such proteins have also been linked to desiccation stress in the bacterium *Bradyrhizobium japonicum* [56].

FFAS analysis of the encoded proteins in the gene neighbourhood of *stlA* on SMG 25 revealed some structural similarities to DnaJ and another type of molecular chaperone for the encoded proteins of genes 3, 4 and 7, while gene 5 encodes a protein with some structural similarity to a human voltage-gated calcium channel to which TspO has been linked, and it also shares a structural homology to Herpes virus latent membrane protein 1 (LMP 1). In addition to DnaJ, the predicted product of gene 7 also exhibited structural similarity to an anti-termination protein from the Qin prophage. This could indicate some of this region was acquired via integration of a phage into the host chromosome. The novelty of the sequences may point to an uncharacterized phage. It is also noteworthy that gene 20 on SMG 25 is predicted to encode a phage-associated protein, while on two of the larger scaffolds from the stool microbiome samples; a gene encoding a phage integrase protein is present, revealing a commonality of such genes in this region. An elegant study by Wang and co-workers, has demonstrated prophage DNA plays a significant role in host resistance to numerous stresses, including osmotic stress [57]. The phage-associated protein on SMG 25 shares 52% identity with a similar protein from *Rhizobium lupini* HPC(L) (100% coverage over 159 amino acids), Interestingly, this organism was recently sequenced following isolation from a saline desert soil [58]. *Rhizobium* species belong to the phylum *Proteobacteria* and, based on taxonomic assignment with MEGAN 4, proteobacterial sequences were found on all the larger scaffolds with an *stlA* homologue (Figure S3.) and may indicate the origin of the phage. Furthermore, a number of genes on SMG 25 are predicted to encode proteins that share a high level of similarity to halophilic and halotolerant microorganisms (Table 1). For example, gene 8 and 9 are predicted to encode hypothetical proteins with similarity to *Pontibacter* *sp.* BAB1700 and a halophilic archaeon, respectively. *Pontibacter* species are halotolerant members of the phylum Bacteroidetes and have been isolated from saline and marine environments, while gene 26 is predicted to encode a protein with similarity to *Halomonas*, a genus of halophilic *Proteobacteria* with biotechnological and medical relevance [59-61]. It seems possible the phage originated in a “salty” environment such as saline soil, a salt lake, a solar saltern or marine ecosystem. 

When compared against all the available samples from the HMP, homologues of the *stlA* sequence were found to be present only in stool microbiome samples. Furthermore, no homologous sequences were found in any bacterial, archaeal, eukaryotic or viral genome sequences, or in any sequenced plasmids. This indicates that *stlA* gene is extremely rare in the sequences tested and may be a gut-specific gene and present only in species of low abundance, as no homologues were found in any of the common or dominant members of the human gut microbiome. In addition, we could only detect *stlA* homologues by PCR in two of 25 metagenomic DNA samples isolated from stool. Gene neighbourhood analysis around the *stlA* homologues revealed they were most often found in combination with genes encoding DnaJ-type molecular chaperones (COG0484), an ankyrin repeat protein (COG0666) or a predicted membrane protein containing a TM-2 domain (COG02314), which is also similar to the gene organisation on SMG 25. 

DnaJ-domain proteins are molecular chaperones that aid protein folding, prevent aggregation and repair damaged proteins following cellular stress [62]. They are members of the heat shock protein (Hsp) family, which have been shown to play important roles in the response to numerous stress conditions including osmotic stress and also can act as co-chaperones by stimulating the activity of other chaperones such as DnaK [63-65]. TM2 domain proteins are composed of a pair of alpha helices connected by a short linker. The function of this domain is unknown; however it occurs in a wide range of protein contexts. It occurs most often on its own or in tandem with another TM2 domain, but interestingly, the third most frequent association is with a DnaJ domain.

Ankyrin-repeat proteins are found across all three domains of life and modulate a number of diverse functions through protein-protein interactions [66]. The repeat has been found in proteins of diverse function [67,68] and these proteins have also been linked to cellular stress responses, including osmotic stress [69-71].

With information gained from gene neighbourhood analysis and distant structural homology we can speculate as to the mechanisms of salt tolerance conferred by *stlA*. Overall, *stlA* and its neighbouring genes share common features that categorise them as stress responsive and may therefore constitute a stress operon. Three of the five encoded unknown proteins share a distant structural homology to chaperones. These chaperones could play a role in protein disaggregation and folding following stress as outlined above, or they could guide StlA through the periplasm and assist in inserting it in the membrane, although the latter situation would require *E. coli* chaperones to function in a similar capacity when *stlA* is cloned in isolation. StlA itself, being a predicted membrane protein could act as a sensor to external stresses or indeed stabilise the outer membrane during stress. It is noteworthy that the most significant homology predicted by FFAS for StlA was to a TspO/MBR protein which is involved in membrane signalling and is associated with voltage-dependent anion channels in mitochondria. 

In conclusion, we have identified a novel salt tolerance gene, *stlA*, from the human gut microbiome through functional screening of a metagenomic library. The gene is rare among the HMP and MetaHit datasets and has no bacterial, archaeal, viral, plasmid or eukaryotic homologues in the current databases. Furthermore, no homologues were found in any non-human metagenome datasets nor in any of the human microbiome datasets (HMP and MetaHit) other than stool, indicating it is gut specific and present in a novel species of low abundance. The *stlA* gene appears to be on a prophage, indicating it may have been acquired (along with some of its neighbouring genes) through a LGT event and may confer a competitive advantage to its particular host species under stressful conditions in the gut or if there is an absence of or deficiency in some of the classical osmotolerance systems, such as in *C. jejuni* [72]. 

Overall this study illustrates the utility of functionally screening metagenomic libraries to assign a function to a completely novel gene and its encoded protein and suggests that novel mechanisms of osmotolerance may exist in different environmental niches. Mining (gut) microbiomes and the development of more sensitive and innovative screening assays will facilitate the discovery of novel stress resistance genes, antibiotics, biopharmaceuticals and biotherapeutics for use in biotechnology, medicine and health [73-77].

## Supporting Information

Figure S1 **Growth in GI-associated stresses.** Growth of *E. coli* MKH13::pCI372 and *E. coli* MKH13::pCI372-*stlA* in LB broth supplemented with numerous stresses associated with the GI (gastrointestinal) tract, such as non-ionic osmotic stress (sucrose and glycerol), low pH and bile. A plasmid-encoded copy of the *stlA* gene did not confer increased tolerance to any of these stresses when expressed in *E. coli* MKH13. Results are presented as the average of triplicate experiments, with error bars being representative of the standard error of the mean (SEM).
(PDF)

Figure S2 **Effect of *stlA* on growth of *Lactococcus lactis* under NaCl stress.** Growth of *L. lactis* MG1363::pCI372 and *L. lactis*MG1363::pCI372-*stlA* in GM17 broth and GM17 broth + 4% NaCl. The *stlA* gene did not provide a protective effect in a Gram-positive host under NaCl stress, which is noteworthy as the StlA protein is predicted to be inserted in the outer membrane. Results are presented as the average of triplicate experiments, with error bars being representative of the standard error of the mean (SEM).
(PDF)

Figure S3 **Taxonomic assignment of scaffold sequences from Human Microbiome Project on which an *stlA* homologue was found.** Scaffold sequences were analysed using BLASTX. The BLASTX results were then downloaded and imported in MEGAN 4 software program which performed taxonomic assignment of each scaffold based on BLAST reads. Two of the shorter scaffolds, indicated with an asterisk (*), could not be assigned any taxonomic classification.
(PDF)

Figure S4 **Comparisons of gene arrangement on SMG 25 fosmid insert and scaffolds with *stlA* homologues from Human Microbiome Project.** The gene neighbourhood region of the *stlA* gene from SMG 25 is compared with gene neighbourhoods from scaffolds with a *stlA* homologue. Homologues of *stlA* were identified through similarity searches (BLASTP; 1e-^50^ cut-off) to the Human Microbiome Project (HMP) datasets. Ten *stlA* homologues were identified and only from the stool microbiome. A legend describing putative gene functions is presented. **Legend**: Red = Hypothetical/membrane protein (*stlA* and homologues); Cream = Hypothetical protein; Dark purple = NADH:ubiquinone oxidoreductase (COG0838); Medium brown = Fucose permease (COG0838); Light blue = Site-specific recombinase, XerD (COG4974); Dark brown = Uncharacterized protein related to capsule biosynthesis enzymes (COG3550); Dark blue/grey = Predicted restriction endonuclease (COG3183); Green = Predicted metal-dependent hydrolase (COG1451); Light-medium blue = Type I site-specific restriction-modification system (COG0610); Light purple = Restriction endonuclease (COG0732); Light maroon = Type I restriction-modification system methyltransferase subunit (COG0286); Light pink = Restriction endonuclease (COG1715); Medium blue = ATP-dependent nuclease (COG3857); Yellow = Predicted membrane protein (TM2 domain) (COG2314); Purple = DnaJ-class molecular chaperone with C-terminal Zn finger domain (COG0484); Brown = Ankyrin repeat protein (COG0666); Dark pink = Serine/threonine protein kinase (COG0515); Olive = Uncharacterized protein with von Willebrand factor (vWF) domain (COG4245); Dark blue = Uncharacterized protein with protein kinase and helix-hairpin-helix DNA-binding domains (COG4248); Light mint green = Virulence protein (COG3943); Mint green = RecB family exonuclease (COG2887); Pink = Predicted oxidoreductase (COG0667); Purple/grey = Hydrolases of the alpha/beta superfamily (COG1073); Light green = Transcriptional regulator, AraC-type DNA-binding domain-containing proteins (COG2207); Orange = Predicted ATPase (AAA+ superfamily) (COG1373); Light grey = Site-specific recombinase, DNA invertase Pin homologs (COG1961); Dark cream = Filamentation induced by cAMP protein (COG3177); Light orange = Predicted helicase (COG4889).
(PDF)

Figure S5 **Multiple sequence alignment of StlA protein sequence with HMP and MetaHit homologues.** Black shading indicates regions of 100% amino acid identity. Putative transmembrane regions for StlA, predicted by TMHMM, are indicated with red boxes. Truncated or partial sequence fragments from HMP were not included (n=4). Information on the protein sequences (A) – (L) is indicated in the legend. (A) StlA protein sequence; (B) SRS053214_LANL_scaffold_17021__gene_42707; (C) SRS024549_LANL_scaffold_1815__gene_4559; (D) C3406971__gene_199744; (E)SRS018656_WUGC_scaffold_544__gene_591;(F)SRS015217_WUGC_scaffold_30292__gene_65222; (G)SRS077730_LANL_scaffold_24345__gene_72567;(H) SRS024075_LANL_scaffold_21370__gene_63545;(I)Baylor_scaffold_22757__gene_50812; (J) C2121591__gene_151559; (K) MetaHit_MH0011_GL0108025 [Complete]locus=scaffold6530_52:7938:8564; (L) MetaHit_V1_GL0100177 [Complete] locus=scaffold36986_1:2178:2888.
(PDF)

File S1 **List of metagenomes available on IMG-M/HMP database to BLAST search query sequences.** Date of last BLAST search against all available metagenome samples was on 19/09/13.
(PDF)

Table S1 **Bacterial strains, plasmids and transposon used in this study.**
(PDF)

Table S2 **Primers used in this study.**
(PDF)
