# Supplementary material for: Functional Environmental Screening of a Metagenomic Library Identifies stlA; A Unique Salt Tolerance Locus from the Human Gut Microbiome
Source: PLoS One. 2013 Dec 12;8(12):e82985. doi: 10.1371/journal.pone.0082985 (PMC3861447; doi:10.1371/journal.pone.0082985)
Supplement: Figure S1 — Growth in GI-associated stresses. Growth of E. coli MKH13::pCI372 and E. coli MKH13::pCI372-stlA in LB broth supplemented with numerous stresses associated with the GI (gastrointestinal) tract, such as non-ionic osmotic stress (sucrose and glycerol), low pH and bile. A plasmid-encoded copy of the stlA gene did not confer increased tolerance to any of these stresses when expressed in E. coli MKH13. Results are presented as the average of triplicate experiments, with error bars being representative of the standard error of the mean (SEM). (PDF) [file pone.0082985.s001.pdf]

Figure S1. Growth in LB broth supplemented with various GI-associated stresses.

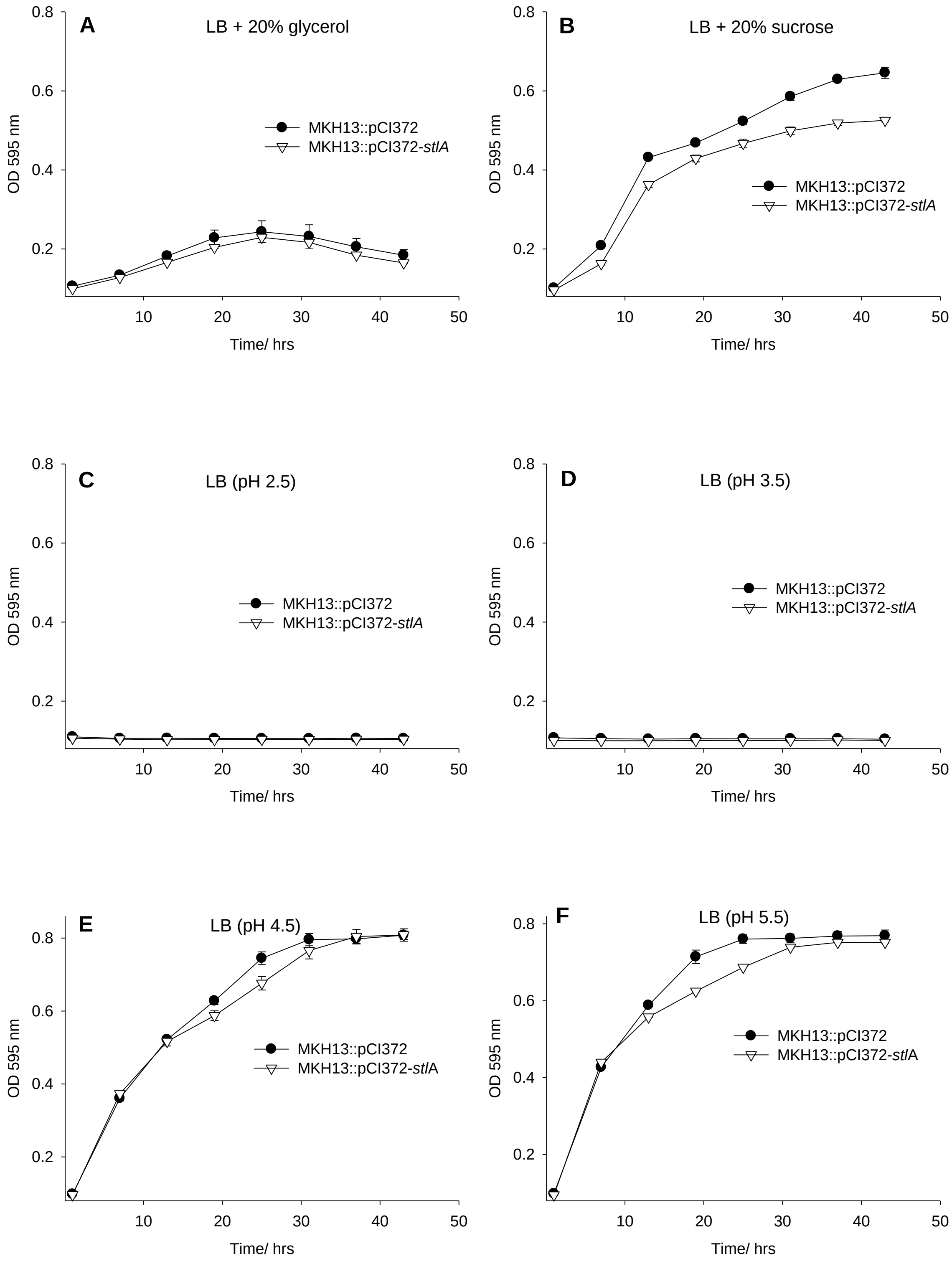

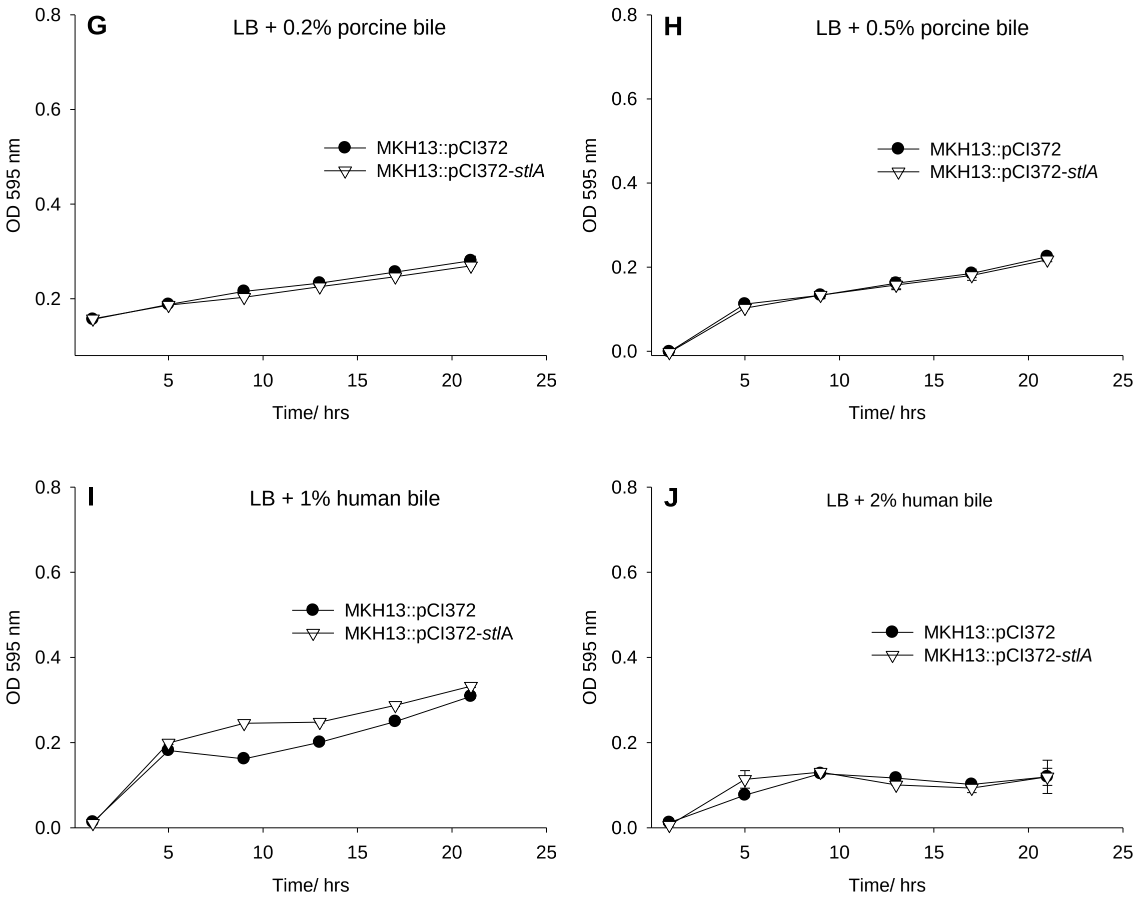

Figure S1. Growth in LB broth supplemented with various GI-associated stresses. Growth of *E. coli* MKH13::pCI372 and *E. coli* MKH13::pCI372-*stlA* in LB broth supplemented with numerous stresses associated with the GI (gastrointestinal) tract, such as non-ionic osmotic stress (sucrose and glycerol), low pH and bile. A plasmid-encoded copy of the *stlA* gene did not confer increased tolerance to any of these stresses when expressed in *E. coli* MKH13. Results are presented as the average of triplicate experiments, with error bars being representative of the standard error of the mean (SEM).
