# Supplementary material for: Functional Environmental Screening of a Metagenomic Library Identifies stlA; A Unique Salt Tolerance Locus from the Human Gut Microbiome
Source: PLoS One. 2013 Dec 12;8(12):e82985. doi: 10.1371/journal.pone.0082985 (PMC3861447; doi:10.1371/journal.pone.0082985)
Supplement: Figure S2 — Effect of stlA on growth of Lactococcus lactis under NaCl stress. Growth of L. lactis MG1363::pCI372 and L. lactisMG1363::pCI372-stlA in GM17 broth and GM17 broth + 4% NaCl. The stlA gene did not provide a protective effect in a Gram-positive host under NaCl stress, which is noteworthy as the StlA protein is predicted to be inserted in the outer membrane. Results are presented as the average of triplicate experiments, with error bars being representative of the standard error of the mean (SEM). (PDF) [file pone.0082985.s002.pdf]

Figure S2. Effect of *stlA* on growth of *Lactococcus lactis* under NaCl stress.

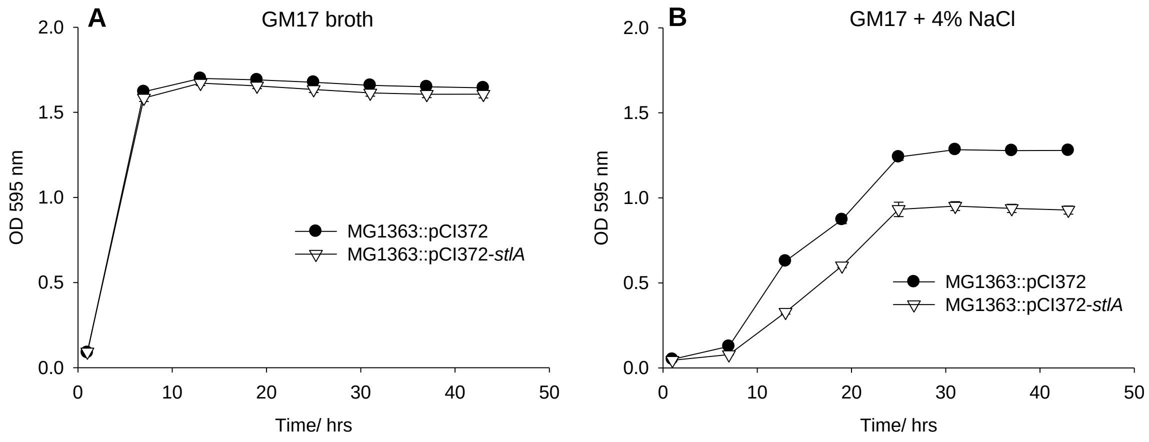

Figure S2. Effect of *stlA* on growth of *Lactococcus lactis* under NaCl stress.

Growth of *L. lactis* MG1363::pCI372 and *L. lactis*MG1363::pCI372-*stlA* in GM17 broth and GM17 broth + 4% NaCl. The *stlA* gene did not provide a protective effect in a Gram-positive host under NaCl stress, which is noteworthy as the StlA protein is predicted to be inserted in the outer membrane. Results are presented as the average of triplicate experiments, with error bars being representative of the standard error of the mean (SEM).
