# Supplementary material for: Functional Environmental Screening of a Metagenomic Library Identifies stlA; A Unique Salt Tolerance Locus from the Human Gut Microbiome
Source: PLoS One. 2013 Dec 12;8(12):e82985. doi: 10.1371/journal.pone.0082985 (PMC3861447; doi:10.1371/journal.pone.0082985)
Supplement: Figure S3 — Taxonomic assignment of scaffold sequences from Human Microbiome Project on which an stlA homologue was found. Scaffold sequences were analysed using BLASTX. The BLASTX results were then downloaded and imported in MEGAN 4 software program which performed taxonomic assignment of each scaffold based on BLAST reads. Two of the shorter scaffolds, indicated with an asterisk (*), could not be assigned any taxonomic classification. (PDF) [file pone.0082985.s003.pdf]

Figure S3. Taxonomic assignment of scaffold sequences from Human Microbiome Project on which an *stlA* homologue was found.

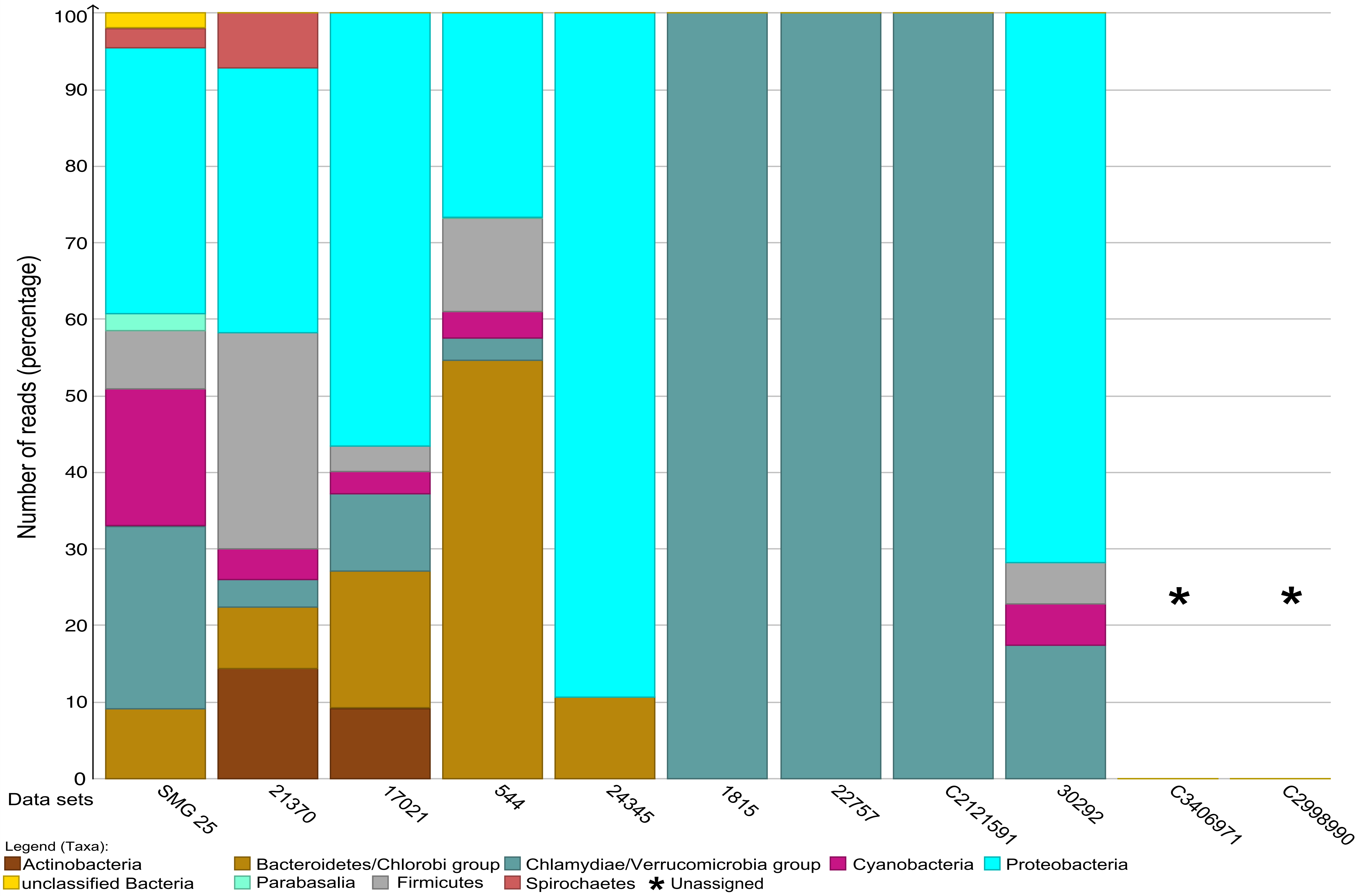

Figure S3. Taxonomic assignment of scaffold sequences from Human Microbiome Project on which an stlA homologue was found. Scaffold sequences were analysed using BLASTX. The BLASTX results were then downloaded and imported in MEGAN 4 software program which performed taxonomic assignment of each scaffold based on BLAST reads. Two of the shorter scaffolds indicated with an asterisk (*) could not be assigned any taxonomic classification.
