# Supplementary material for: Functional Environmental Screening of a Metagenomic Library Identifies stlA; A Unique Salt Tolerance Locus from the Human Gut Microbiome
Source: PLoS One. 2013 Dec 12;8(12):e82985. doi: 10.1371/journal.pone.0082985 (PMC3861447; doi:10.1371/journal.pone.0082985)
Supplement: Figure S4 — Comparisons of gene arrangement on SMG 25 fosmid insert and scaffolds with stlA homologues from Human Microbiome Project. The gene neighbourhood region of the stlA gene from SMG 25 is compared with gene neighbourhoods from scaffolds with a stlA homologue. Homologues of stlA were identified through similarity searches (BLASTP; 1e-50 cut-off) to the Human Microbiome Project (HMP) datasets. Ten stlA homologues were identified and only from the stool microbiome. A legend describing putative gene functions is presented. Legend: Red = Hypothetical/membrane protein (stlA and homologues); Cream = Hypothetical protein; Dark purple = NADH:ubiquinone oxidoreductase (COG0838); Medium brown = Fucose permease (COG0838); Light blue = Site-specific recombinase, XerD (COG4974); Dark brown = Uncharacterized protein related to capsule biosynthesis enzymes (COG3550); Dark blue/grey = Predicted restriction endonuclease (COG3183); Green = Predicted metal-dependent hydrolase (COG1451); Light-medium blue = Type I site-specific restriction-modification system (COG0610); Light purple = Restriction endonuclease (COG0732); Light maroon = Type I restriction-modification system methyltransferase subunit (COG0286); Light pink = Restriction endonuclease (COG1715); Medium blue = ATP-dependent nuclease (COG3857); Yellow = Predicted membrane protein (TM2 domain) (COG2314); Purple = DnaJ-class molecular chaperone with C-terminal Zn finger domain (COG0484); Brown = Ankyrin repeat protein (COG0666); Dark pink = Serine/threonine protein kinase (COG0515); Olive = Uncharacterized protein with von Willebrand factor (vWF) domain (COG4245); Dark blue = Uncharacterized protein with protein kinase and helix-hairpin-helix DNA-binding domains (COG4248); Light mint green = Virulence protein (COG3943); Mint green = RecB family exonuclease (COG2887); Pink = Predicted oxidoreductase (COG0667); Purple/grey = Hydrolases of the alpha/beta superfamily (COG1073); Light green = Transcriptional regulator, AraC-type DNA-bind [file pone.0082985.s004.pdf]

Figure S4. Comparisons of gene arrangement on SMG 25 fosmid insert and scaffolds with *stlA* homologues from Human Microbiome Project.

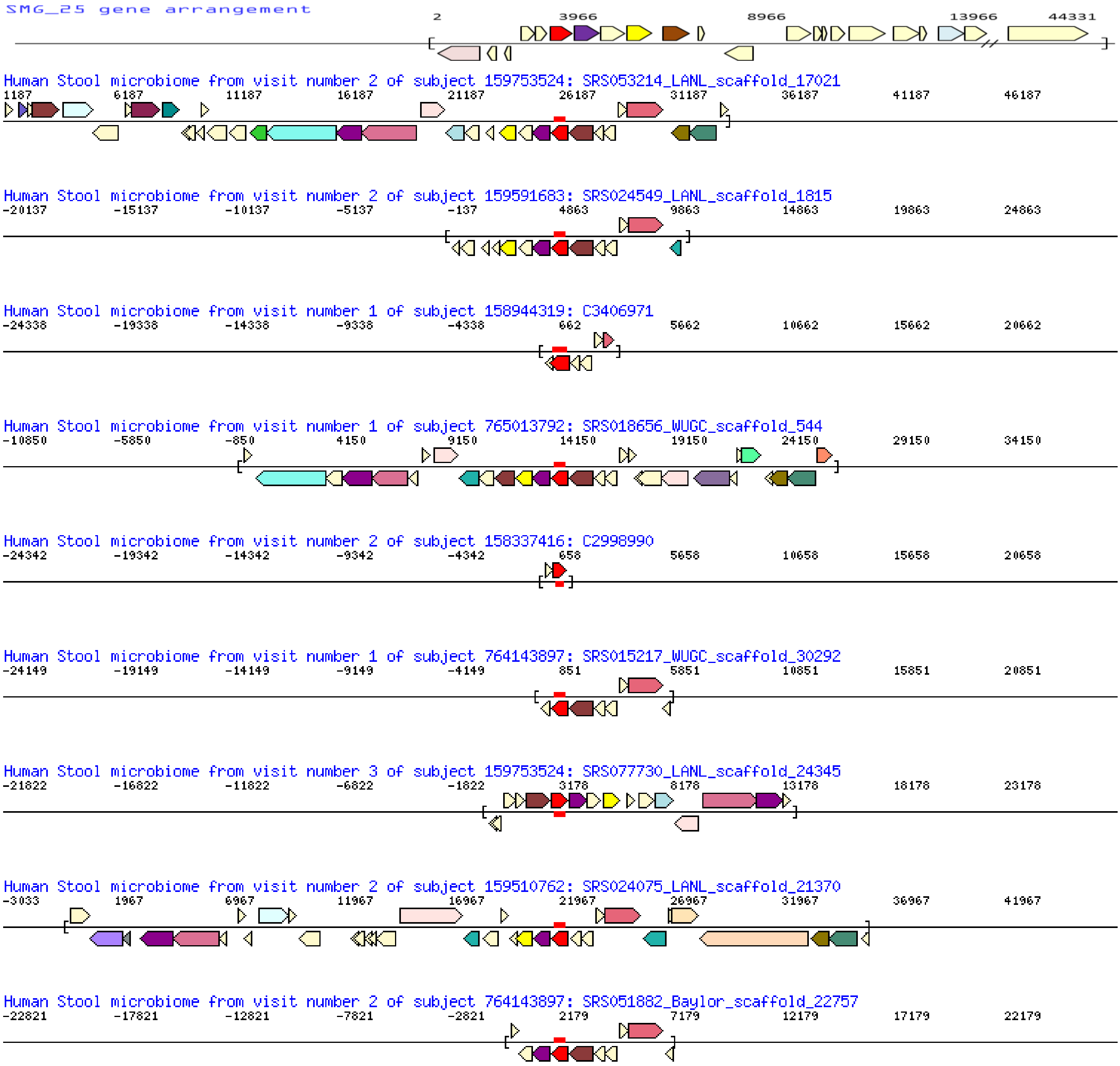

Figure S4. The gene neighbourhood region of the *stlA* gene from SMG 25 is compared with gene neighbourhoods from scaffolds with an *stlA* homologue. Homologues of *stlA* were identified through similarity searches (BLASTP; 1e-^{50} cut-off) to the Human Microbiome Project (HMP) datasets. Ten *stlA* homologues were identified and only from the stool microbiome. A legend describing putative

gene functions is presented below datasets. Ten *stlA* homologues were identified and only from the stool microbiome. A legend describing putative gene functions is presented below.

Legend: Red = Hypothetical/membrane protein (*stlA* and homologues); Cream = Hypothetical protein; Dark purple = NADH:ubiquinone oxidoreductase (COG0838); Medium brown = Fucose permease (COG0838); Light blue = Site-specific recombinase, XerD (COG4974); Dark brown = Uncharacterized protein related to capsule biosynthesis enzymes (COG3550); Dark blue/grey = Predicted restriction endonuclease (COG3183); Green = Predicted metal-dependent hydrolase (COG1451); Light-medium blue = Type I site-specific restriction-modification system (COG0610); Light purple = Restriction endonuclease (COG0732); Light maroon = Type I restriction-modification system methyltransferase subunit (COG0286); Light pink = Restriction endonuclease (COG1715); Medium blue = ATP-dependent nuclease (COG3857); Yellow = Predicted membrane protein (TM2 domain) (COG2314); Purple = DnaJ-class molecular chaperone with C-terminal Zn finger domain (COG0484); Brown = Ankyrin repeat protein (COG0666); Dark pink = Serine/threonine protein kinase (COG0515); Olive = Uncharacterized protein with von Willebrand factor (vWF) domain (COG4245); Dark blue = Uncharacterized protein with protein kinase and helix-hairpin-helix DNA-binding domains (COG4248); Light mint green = Virulence protein (COG3943); Mint green = RecB family exonuclease (COG2887); Pink = Predicted oxidoreductase (COG0667); Purple/grey = Hydrolases of the alpha/beta superfamily (COG1073); Light green = Transcriptional regulator, AraC-type DNA-binding domain-containing proteins (COG2207); Orange = Predicted ATPase (AAA+ superfamily) (COG1373); Light grey = Site-specific recombinase, DNA invertase Pin homologs (COG1961); Dark cream = Filamentation induced by cAMP protein (COG3177); Light orange = Predicted helicase (COG4889).
