# Supplementary material for: Functional Environmental Screening of a Metagenomic Library Identifies stlA; A Unique Salt Tolerance Locus from the Human Gut Microbiome
Source: PLoS One. 2013 Dec 12;8(12):e82985. doi: 10.1371/journal.pone.0082985 (PMC3861447; doi:10.1371/journal.pone.0082985)
Supplement: Figure S5 — Multiple sequence alignment of StlA protein sequence with HMP and MetaHit homologues. Black shading indicates regions of 100% amino acid identity. Putative transmembrane regions for StlA, predicted by TMHMM, are indicated with red boxes. Truncated or partial sequence fragments from HMP were not included (n=4). Information on the protein sequences (A) – (L) is indicated in the legend. (A) StlA protein sequence; (B) SRS053214_LANL_scaffold_17021__gene_42707; (C) SRS024549_LANL_scaffold_1815__gene_4559; (D) C3406971__gene_199744; (E)SRS018656_WUGC_scaffold_544__gene_591;(F)SRS015217_WUGC_scaffold_30292__gene_65222; (G)SRS077730_LANL_scaffold_24345__gene_72567;(H) SRS024075_LANL_scaffold_21370__gene_63545;(I)Baylor_scaffold_22757__gene_50812; (J) C2121591__gene_151559; (K) MetaHit_MH0011_GL0108025 [Complete]locus=scaffold6530_52:7938:8564; (L) MetaHit_V1_GL0100177 [Complete] locus=scaffold36986_1:2178:2888. (PDF) [file pone.0082985.s005.pdf]

Figure S5. Multiple sequence alignment of StlA protein sequence with HMP and MetaHit homologues.

```
                                        10        20        30        40        50        60        70        80        90
                                                                                     *         *     *********   * *   *** ** **
(A) -----------------MDTVRQRQGLGIKRSMLTALLMGFCCLFYAPEAFSQEESSALPKQSGLDEDMAKGVALVKQRYGEQLRNQYMGGATTAKEYAIGALRMENDIAKAEA  97
(B) -----------------MNTIRHVQGAWFKKRILTVLLLGVCCLFYPHNALSQDEPSTPPQKTGMQLDMEKGMEQARKRSGDQLYNQYMGGATTAGQYAIGAAQMENAIAEAER  97
(C) -----------------MNTVRQRQRLGIKRSILTALLMGFCFLFYTPEAFSQDEPGSPPKKSGLAEDMEKGIAQANQRYGQQLYQQYMGGATTAKECAIGVLRMENAIAEAEG  97
(D) MQAKSYPDGINTNTNITMNTVRQRQRLGIKRSILTALLMGFCCLFYAPEAFSQDEPGAPSQRTAMDDDFDKSMAQARQQYGNRLYQQYMGGATTAKERAIGVLRMENAIAEAEG 114
(E) -----------------MNTVRQRQRLGIKRSILTALLMGFCFLFYTPEAFSQDEPGSPPKKSGLAEDMEKGIAQADQRYGQQLYQQYMGGATTAKECAIGVLRMENAIAEAEG  97
(F) -----------------MNTVRQRQRLGIKRSILTALLMGFCFLFYTPEAFSQDEPGSPPKKSGLAEDMEKGIAQANQRYGQQLYQQYMGGATTAKECAIGVLRMENAIAEAEG  97
(G) -----------------MNTIRHVQGAWFKKRILTVLLLGVCCLFYPHNALSQDEPSTPPQKTGMQLDMEKGMEQARKRSGDQLYNQYMGGATTAGQYAIGAAQMENAIAEAER  97
(H) -------------------------------------MGFCFLFYTPEAFSQDEPGSPPKKSGLDEDMEKGIAQARQQYGNRLYQQYMGGATTAKERAIGVLRMENAIAEAEG  76
(I) -----------------MNTVRQRQRLGIKRSILTALLMGFCFLFYTPEAFSQDEPGSPPKKSGLAEDMEKGIAQANQRYGQQLYQQYMGGATTAKECAIGVLRMENAIAEAEG  97
(J) MQAKSCREAINTNTNITMNTVRQRQRLRIKRSILTALLMGFCFLFYTPEAFSQEESSALPKQSGLDEDMAKGIALAKQRYGEQLRNQYMGGATTAKEYAIGALRMENDIAKAEG 114
(K) -------------------------------------------------------------MQAEFDKGTAQNRKRYGDQVYDQYMGGTTTAGQKAVGAAQMENAIAVAEG  50
(L) -------------------------------------MGFCFLFYTPEAFSQDEPGSPPKKSGLDEDMEKGMAQARQQYGNRLYQQYMGGATTAKERAIGALRMENAIAEAEG  76

     *    *  ** ***     *       * ***  **  ***** ***    **    *            **   *** *   ** ** *    ****** *****  ****** * **
(A) EGNTSLANALRQQLAMAQRTNEWYNGELQQKADAGDTRAQRELDEYYAFMKTVEGSSPAGSFPFLISIISGLLLYGYIVLLSPKDATINRKTLIPWCVGLAVFDMLGNWVNQSW 211
(B) NGNTGRANMLREQLARANSNNQWYLNELKQKADAGDAGAQRELNEYYELIKTSTPSLP--AVPFVVSIIFGLLFYWCMVLFSPRDTILNRKTLILWCVGLTVFDMLGNLTNSSW 209
(C) KGDTAQANALRQQLARAQSNNEWSIGHLQQKANVGDTRAQRELDEYYAFMKTVEGSSPGEAFPFLISIISGLLLYGYIVLLSPKDATINRKTLIPWCVGLAIFDMLGNWVNQSW 211
(D) KGDTAQANALRQQLAMAQSNNEWSTGHLQQKANAGDTRAQRELDEYYAFMKTVEGSSPGEAFPSLISIISGLLLYGYIVLLSPKDATINRKTLIPWCVGLAIFDMLGNWVNQSW 228
(E) KGDTAQANALRQQLAMAQSNNEWSIGHLQQKANVGDTRAQRELDEYYAFMKTVEGSSPGEAFPFLISIISGLLLYGYIVLLSPKDATINRKTLIPWCVGLAIFDMLGNWVNQSW 211
(F) KGDTAQANALRQQLARAQSNNEWSIGHLQQKANVGDTRAQRELDEYYAFMKTVEGSSPGEAFPFLISIISGLLLYGYIVLLSPKDATINRKTLIPWCVGLAIFDMLGNWVNQSW 211
(G) NGNTGRANMLREQLARANSNNQWYLNELKQKADAGDAGAQRELNEYYELIKTSTPSLP--AVPFVVSIIFGLLFYWCMVLFSPRDTILNRKTLILWCVGLTVFDMLGNLTNSSW 209
(H) KGDTAQANALRQQLAMAQSNNEWSTGHLQQKANAGDTRAQRELDEYYAFMKTVEGSSPGEAFPFLISIVSGLLLYGYIVLLSPKDATINRKTLIPWCVGLAIFDMLGNWVNQSW 190
(I) KGDTAQANALRQQLARAQSNNEWSIGHLQQKANVGDTRAQRELDEYYAFMKTVEGSSPGEAFPFLISIISGLLLYGYIVLLSPKDATINRKTLIPWCVGLAIFDMLGNWVNQSW 211
(J) EGNTELANMLRQQLAVQQANQISNTELQQKANVGDTRAQRELDEYYAFMKTVEGSSPGEAFPFLISIISGLLLYGYIVLLSPKDATINRKTLIPWCVGLAIFDMLGNWVNQSW 228
(K) KGDTAQADMLRQQLARANSNNQWYLNELKQKADAGDAGAQRELNEYYELIKTSTPSLP--AVPFVVSIIFGLLFYWCMVLFSPRDTILNRKTLILWCVGLTVFDMLGNLTNSSW 162
(L) KGDSTQANYLRQQLAMAQSNNEWANGKLQQKANAGDTRAQRELDEYYAFMKTVKGSSSADLFPFLISIISGLLLYGYIVLLSPKDATINRKTLIPWCVGLAIFDMLGNWVNQSW 190

    **** * * **   ** *  ****   **  ***
(A) LFLFVEILIILVVARNFQCSWKRSFAILGLMLVSILILGGLLPLFF 257
(B) LFLFGEVLAILLIARSFKYSWKRTFSFLGIVLVSVLILGGLLTTFL 255
(C) LFLFVEILVILVVARNFQCSWKRSFAILGLMLVSILILGGLLPLFF 257
(D) LFLFVEILVILVVARNFQCSWKRSFAILGLMLVSILILGGLLPLFF 274
(E) LFLFVEILVILVVARNFQCSWKRSFAILGLMLVSILILGGLLPLFL 257
(F) LFLFVEILVILVVARNFQCSWKRSFAILGLMLVSILILGGLLPLFF 257
(G) LFLFGEVLAILLIARSFKYSWKRTFSFLGIVLVSVLILGGLLTTFL 255
(H) LFLFVEILIILVVARNFQCSWKRSFAILGLMLVSILILGGLLPLFF 236
(I) LFLFVEILVILVVARNFQCSWKRSFAILGLMLVSILILGGLLPLFF 257
(J) LFLFVEILVILVVARNFQCSWKRSFAILGLMLVSILILGGLLPLFL 274
(K) LFLFGEVLAILLIARSFKYSWKRTFSILGIVLVSVLILGGLLTTFL 208
(L) LFLFVEILVILVVARNFQCSWKRSFAILGLMLVS------------ 224
```

Figure S5. Multiple protein sequence alignment of StlA and homologous sequences from Human Stool Microbiome from Human Microbiome Project (HMP) and MetaHit. Black shading indicates regions of 100% amino acid identity. Putative transmembrane regions for StlA, predicted by TMHMM, are indicated with red boxes. Truncated or partial sequence fragments from HMP were not included (n=4). Information on the protein sequences (A) – (L) is indicated in the legend below.

Legend:

(A) = StlA protein sequence (B) SRS053214_LANL_scaffold_17021__gene_42707 (C) SRS024549_LANL_scaffold_1815__gene_4559 (D) C3406971__gene_199744 (E) SRS018656_WUGC_scaffold_544__gene_591 (F) SRS015217_WUGC_scaffold_30292__gene_65222 (G) SRS077730_LANL_scaffold_24345__gene_72567 (H) SRS024075_LANL_scaffold_21370__gene_63545 (I) Baylor_scaffold_22757__gene_50812 (J) C2121591__gene_151559 (K) MetaHit_MH0011_GL0108025 [Complete]locus=scaffold6530_52:7938:8564 (L) MetaHit_V1_GL0100177 [Complete] locus=scaffold36986_1:2178:2888
