# Supplementary material for: Functional Environmental Screening of a Metagenomic Library Identifies stlA; A Unique Salt Tolerance Locus from the Human Gut Microbiome
Source: PLoS One. 2013 Dec 12;8(12):e82985. doi: 10.1371/journal.pone.0082985 (PMC3861447; doi:10.1371/journal.pone.0082985)
Supplement: File S1 — List of metagenomes available on IMG-M/HMP database to BLAST search query sequences. Date of last BLAST search against all available metagenome samples was on 19/09/13. (PDF) [file pone.0082985.s006.pdf]

LIST OF METAGENOMES AVAILABLE ON IMG-M/HMP DATABASE TO BLAST SEARCH QUERY SEQUENCES (19/09/13)

Soil microbial communities from switchgrass rhizosphere
Switchgrass soil microbial communities from Univ ... Rhizosphere soil sample from switchgrass (Panicum virgatum)) (MER-FS) (*)[F]

Arabidopsis rhizosphere microbial communities from University of North Carolina

Arabidopsis rhizosphere microbial communities from ... (Arabidopsis Col-0 young rhizosphere DNA, March 2102 Assem) (MER-FS) (*)[D]

Arabidopsis rhizosphere microbial communities from Universit ... na, sample Mutant cpr5 (cpr5 454/Illumina combined assembly) (*)[D]

Arabidopsis rhizosphere microbial communities from ... North Carolina, sample Mutant cpr5 (cpr5 mutant 454 Newbler) (MER-FS) (*)[D]

Arabidopsis rhizosphere microbial communities from ... of North Carolina, sample Wild type Col-0 (454 only Newbler) (MER-FS) (*)[D]

Arabidopsis rhizosphere microbial communities from ... ere microbiome-wild type Col-0 454/Illumina 2011 July Assem) (MER-FS) (*)[D]

Arabidopsis rhizosphere microbial communities from ... ample Wild type Col-0 (Col-0 454/Illumina combined assembly) (MER-FS) (*)[D]

Arabidopsis rhizosphere microbial communities from ... zosphere (Arabidopsis Col-0 old rhizosphere, Nov 2011 assem) (MER-FS) (*)[D]

Arabidopsis rhizosphere microbial communities from ... izosphere (Arabidopsis cpr5 old rhizosphere, Nov 2011 assem) (MER-FS) (*)[D]
Arabidopsis rhizosphere microbial communities from ... osphere (Arabidopsis cpr5 young rhizosphere, Nov 2011 assem) (MER-FS) (*)[D]

Blue grama grass rhizosphere eukaryotic and microbial communities from Sevilleta Long Term Ecological Research site, New Mexico, US

Blue Grama Grass Combined Assembly (MER-FS) (*)[D]

Blue Grama Grass Combined Assembly (MER-FS) (*)[D]
Blue Grama RHZ soil N,W,P treatment plot 28 (MER-FS) (*)[D]

Soil microbial communities from switchgrass rhizosphere

Maize rhizosphere soil microbial communities from ... Urbana, IL (Soil sample from rhizosphere of corn (Zea mays)) (MER-FS) (*)[F]

Miscanthus rhizosphere soil microbial communities ... bana, IL (Rhizosphere soil sample of Miscanthus x giganteus) (MER-FS) (*)[F]

Soil microbial communities from Miscanthus in Kellogg Biological Station, MSU

Miscanthus rhizosphere microbial communities from ... ate 1: eDNA_1 (Rhizosphere replicate 1 April 2011 assembly) (MER-FS) (*)[D]

Miscanthus rhizosphere microbial communities from ... eplicate 2: eDNA_1 (Rhizo 2 January 2011 combined assembly) (MER-FS) (*)[D]

Switchgrass rhizosphere microbial community from Michigan, US

Switchgrass rhizosphere microbial community from M ... ake rhizosphere BV2.2 (BV2.2 January 2011 combined assembly) (MER-FS) (*)[D]

Switchgrass rhizosphere microbial community from M ... e rhizosphere RL2 (Rhizo RL2 January 2011 combined assembly) (MER-FS) (*)[D]

Switchgrass rhizosphere microbial community from M ... e Lake rhizosphere RL2 (Rhizosphere RL2 April 2011 assembly) (MER-FS) (*)[D]

Switchgrass rhizosphere microbial community from Michigan, US, sample from East Lansing bulk soil (MER-FS) (*)[D]

Switchgrass rhizosphere microbial community from M ... ng bulk soil (Bulk soil GOTP January 2011 combined assembly) (MER-FS) (*)[D]

Switchgrass rhizosphere microbial community from M ... , US, sample from East Lansing, 10341 (454/Illumina contigs) (MER-FS) (*)[D]

Endophytic microbiome from Rice

Endophytic microbiome from Rice (MER-FS) (*)[D]

Switchgrass rhizosphere microbial community from Michigan, US

Switchgrass rhizosphere microbial community from Michigan, U ... ea, Rhizosphere BV2.1 (BV2.1 January 2011 combined assembly) (*)[D]

Unclassified

11_1 (MER-FS) (*)[D]

12_2 (MER-FS) (*)[D]

Termite hindgut microbiota from Amitermes wheeleri in the Arizona desert and from Nasutitermes corniger in Florida

Amitermes wheeleri hindgut microbiota, collected f ... Arizona desert, USA (Metagenome Assembly) (Newbler assembly) (MER-FS) (*)[D]

Amitermes wheeleri hindgut microbiota, collected f ... the Arizona desert, USA (Metatranscriptome assembly_May2011) (MER-FS) (*)[D]

Nasutitermes corniger hindgut microbiota, obtained ... USA (Metagenome and Metatranscriptome Co-assembly_Oct 2011) (MER-FS) (*)[D]

Nasutitermes corniger hindgut microbiota, obtained ... ony in Florida, USA (Metagenome assembly) (Newbler assembly) (MER-FS) (*)[D]

Nasutitermes corniger hindgut microbiota, obtained from a la ... colony in Florida, USA (Metatranscriptome assembly_May2011) (*)[D]

Termite hindgut microbiota from Amitermes wheeleri in the Arizona desert and from Nasutitermes corniger in Florida

Amitermes wheeleri hindgut microbiota, collected f ... USA (Metagenome and Metatranscriptome Co-assembly_Oct 2011) (MER-FS) (*)[D]

Unclassified

Gut microbiome of Costa Rica Nasutitermes termites from P3 luminal contents

Hindgut microbiome of Nasutitermes sp. (Costa Rica) (*)[D]

Hindgut microbiome of Nasutitermes sp. (Costa Rica) (version 3) (*)[D]

Hindgut microbiome of Nasutitermes sp. (Costa Rica), fosmids (*)[D]

Unclassified

Gut microbiome of Panchlora sp. from refuse piles of leaf-cutter ants in Panama

Panchlora_hindgut_metagenome (Panchlora hindgut, UnAmp Illumina Only Nov 2011 assem) (MER-FS) (*)[D]

Unclassified

Honey Bee colony (Honey Bee colony, Assem Contigs Jan 2012 Assem) (MER-FS) (*)[D]

Honey Bee colony (Honey Bee colony, Assem Scaffolds Jan 2012 Assem) (MER-FS) (*)[D]

Metagenomics and metatranscriptomics of the gut microbiota of higher termites

Termite gut Cu122C (Termite gut Cu122C, ASSEMBLY_DATE=20130606) (MER-FS) (*)[D]

Termite gut Cu122M (Termite gut Cu122M, ASSEMBLY_DATE=20130606) (MER-FS) (*)[D]

Termite gut Cu122P1 (MER-FS) (*)[D]

Termite gut Cu122P3 (Termite gut Cu122P3, ASSEMBLY_DATE=20130606) (MER-FS) (*)[D]

Termite gut Cu122P4 (Termite gut Cu122P4, ASSEMBLY_DATE=20130606) (MER-FS) (*)[D]

Termite gut Nc150C (Termite gut Nc150C, ASSEMBLY_DATE=20130618) (MER-FS) (*)[D]

Termite gut Nc150M (Termite gut Nc150M, ASSEMBLY_DATE=20130606) (MER-FS) (*)[D]

Termite gut Nc150P1 (Termite gut Nc150P1, ASSEMBLY_DATE=20130606) (MER-FS) (*)[D]

Termite gut Nc150P1 (Termite gut Nc150P1, ASSEMBLY_DATE=20130606) (MER-FS) (*)[D]

Termite gut Nc150P3 (Termite gut Nc150P3, ASSEMBLY_DATE=20130420) (MER-FS) (*)[D]

Termite gut Nc150P4 (Termite gut Nc150P4, ASSEMBLY_DATE=20130425) (MER-FS) (*)[D]

Termite gut Nc150P5 (Termite gut Nc150P5, ASSEMBLY_DATE=20130425) (MER-FS) (*)[D]

Gut microbiome of honey bee

Tucson sample 1, colony 176 (*)[D]

Neotropical beetle gut microbiome from Costa Rica

Intestinal microbiome of Neotropical beetle Adult ... 4BA+4MSA) (Adult (4MA+4BA+4MSA) Oct 2011 assem - Assem Ctgs) (MER-FS) (*)[D]

Intestinal microbiome of Neotropical beetle Adult (4MA+4BA+4MSA) (Adult (4MA+4BA+4MSA) Oct 2011 assem) (MER-FS) (*)[D]

Intestinal microbiome of Neotropical beetle Galler ... 3BSU) (Gallery material (4MSU+4BSU+3MSU+3BS) Dec 2011 assem) (MER-FS) (*)[D]

Intestinal microbiome of Neotropical beetle Larvae ... iome of Neotropical beetle Larvae (1ML+1BSL) Oct 2011 assem) (MER-FS) (*)[D]

Intestinal microbiome of Neotropical beetle Larvae (1ML+1BSL) (Larvae (1ML+1BSL) August 2011 assem) (MER-FS) (*)[D]

Intestinal microbiome of Neotropical beetle Larvae (2ML+2BL) (Larvae (2ML+2BL) August 2011 assem) (MER-FS) (*)[D]

Intestinal microbiome of Neotropical beetle Larvae (3ML+3BL) (Larvae (3ML+3BL)Sept 2011 Assem - Assem Ctgs) (MER-FS) (*)[D]

Intestinal microbiome of Neotropical beetle Larvae (3ML+3BL) (Larvae (3ML+3BL)Sept 2011 Assem) (MER-FS) (*)[D]

Intestinal microbiome of Neotropical beetle Larvae (4BL+4ML+4MSL) (Larvae (4BL+4ML+4MSL) Sept. 2011 assem) (MER-FS) (*)[D]

Intestinal microbiome of Neotropical beetle Larvae (5ML+5BL) (Larvae (5ML+5BL) Oct 2011 assem - Assem Ctgs) (MER-FS) (*)[D]

Intestinal microbiome of Neotropical beetle Larvae (5ML+5BL) (Larvae (5ML+5BL) Oct 2011 assem) (MER-FS) (*)[D]

Gut microbiome of Panchlora sp. from refuse piles of leaf-cutter ants in Panama

Panchlora_midgut_metagenome (Midgut August 2011 assem) (MER-FS) (*)[D]

Gut microbiome of Anoplophora glabripennis

Larvae (6 May 2010 assembly) (MER-FS) (*)[D]

Unclassified

Fungus garden microbial communities from Apterostigma dentigerum

Apterostigma fungus garden Combined (MER-FS) (*)[D]

Fungus garden microbial communities from Cyphomyrmex longiscapus

Cyphomyrmex longiscapus fungus garden (MER-FS) (*)[D]

Fungus garden microbial communities from Trachymyrmex in Gamboa, Panama

Trachymyrmex fungus garden (MER-FS) (*)[D]

Garden dump

Leaf cutter ant microbial communities from fungus growing ant-garden

Atta colombica fungus garden Top (MER-FS) (*)[D]

Unclassified

Atta columbica fungus garden and dump (Dump bottom) (MER-FS) (*)[D]

Atta columbica fungus garden and dump (Dump top) (MER-FS) (*)[D]

Unclassified

Dendroctonus frontalis Fungal community (MER-FS) (*)[D]

Unclassified

Fungus gallery microbial communities from Dendroctonus ponderosae

Dendroctonus ponderosae fungus gallery (Hybrid pine) (MPB hybrid gallery) (MER-FS) (*)[D]

Mountain Pine Beetle microbial communities from Grand Prairie, Alberta

Mountain Pine Beetle microbial communities from Gr ... rairie, Alberta, sample from Hybrid pine (MPB hybrid beetle) (MER-FS) (*)[D]

Xyleborus affinis microbiome from Bern, Switzerland

Xyleborus affinis microbiome from Bern, Switzerland, sample of adult community (Ambrosia beetle adult) (MER-FS) (*)[D]

Xyleborus affinis microbiome from Bern, Switzerland, sample of gallery community (Gallery community) (MER-FS) (*)[D]

Xyleborus affinis microbiome from Bern, Switzerland, sample of larvae (Larvae community) (MER-FS) (*)[D]

Unclassified

Dendroctonus frontalis Bacterial community

Dendroctonus frontalis bacterial community (MER-FS) (*)[D]

Unclassified

Mountain Pine Beetle microbial communities from Grand Prairie, Alberta

Mountain Pine Beetle microbial communities from Mc ... olumbia, Canada, sample from Lodgepole pine (Lodgepole pine) (MER-FS) (*)[D]

Unclassified

Sirex noctilio microbiome

Sirex noctilio microbiome from Pennsylvania, sample of adult community (Adult June 2010 assembly) (MER-FS) (*)[D]

Unclassified

Spirochaeta_SingleCell

Bankia setacea (Shipworm) - single cell (*)[D]

Bankia setacea microbiome

Bankia setacea gill microbiome from Puget Sound WA (MER-FS) (*)[D]

Bankia setacea gill microbiome from Puget Sound, W ... mple from sunken wood 10383 (Combined 454/Illumina assembly) (MER-FS) (*)[D]

Marine microbial communities from multiple species of wood-boring bivalves (shipworms)

Bankia setacea gill microbiome from Puget Sound WA ... setacea gill BSg1 (Bankia setacea gill BSg1, Nov 2011 assem) (MER-FS) (*)[D]

Bankia setacea gill microbiome from Puget Sound WA ... setacea gill BSg4 (Bankia setacea gill BSg4, Nov 2011 assem) (MER-FS) (*)[D]

Bankia setacea microbiome

Bankia setacea gill microbiome from Puget Sound WA, LANL assembly (MER-FS) (*)[D]

Marine microbial communities from multiple species of wood-boring bivalves (shipworms)

Bankia setacea gill microbiome from Puget Sound WA ... setacea gill BSg2 (Bankia setacea gill BSg2, Nov 2011 assem) (MER-FS) (*)[D]

Bankia setacea gill microbiome from Puget Sound WA ... setacea gill BSg3 (Bankia setacea gill BSg3, Nov 2011 assem) (MER-FS) (*)[D]

Bankia setacea gill microbiome from Puget Sound WA ... setacea gill BSg3 (Bankia setacea gill BSg3, Nov 2011 assem) (MER-FS) (*)[D]

Viral community from bovine rumen

Bovine rumen viral communities from University of Illinois Dairy Farm in Urbana, IL, Cow rumen 6993 (Cow 6993) (MER-FS) (*)[D]

Bovine rumen viral communities from University of Illinois Dairy Farm in Urbana, IL, Cow rumen 7664 (Cow 7664) (MER-FS) (*)[D]

Bovine rumen viral communities from University of Illinois Dairy Farm in Urbana, IL, Cow rumen 7887 (Cow 7887) (MER-FS) (*)[D]

Rumen Metagenome Switchgrass associated / selected GH contigs

Sample 470 (HiSeq_Scaffolds_Complete_Set) (MER-FS) (*)[D]

Forestomach microbiome of Macropus eugenii

Macropus eugenii forestomach microbiome from Canberra, Australia, sample 10206 (*)[D]

Intestinal microbiome of Mouse lean and obese

Mouse Gut Community lean1 (*)[D]

Mouse Gut Community lean2 (*)[D]

Mouse Gut Community lean3 (*)[D]

Mouse Gut Community ob1 (*)[D]

Mouse Gut Community ob2 (*)[D]

Wild Panda gut microbiome from Shaanxi, China

Wild Panda gut microbiome from Saanxi China, sample from individual w1 (*)[P]

Wild Panda gut microbiome from Saanxi China, sample from individual w2 (GB1) (*)[P]

Wild Panda gut microbiome from Saanxi China, sample from individual w5 (GB9) (*)[P]

Fecal microbiome of Canis familiaris

sample 1 (MER-FS) (*)[D]

sample 2 (MER-FS) (*)[D]

Svalbard Reindeer rumen metagenome

Sample 549 (Fosmids) (*)[D]

Sample 549 (R.t.platyrhynchus_ua) (MER-FS) (*)[D]

Sample 549 (Svalbard Reindeer rumen metagenome) (MER-FS) (*)[D]

Crop microbiome from Hoatzin

Epithelial fraction 12 (Epithelial 12 combined assembly) (MER-FS) (*)[D]

Epithelial fraction 12 (Hoatzin crop microbiome epithelium fraction 12 (Dec 2011 assem Abyss hiseq+gaii)) (MER-FS) (*)[D]

Epithelial fraction 12 (Hoatzin crop microbiome epithelium fraction 12 (July 2011 assem HYIP.1652.1 k85) (MER-FS) (*)[D]

Hoatzin crop microbial communities from Cojedes, Venezuel, sample from Protozoan 21 (Protozoan 21 MDA) (*)[D]

Hoatzin crop microbial communities from Cojedes, Venezuela, ... from fiber fraction 12 (Fiber fraction 12 combined assembly) (*)[D]

Hoatzin crop microbial communities from Cojedes, Venezuela, ... from fiber fraction 14 (Fiber fraction 14 combined assembly) (*)[D]

Unclassified

Rhopaloeides odorabile metagenome replicate 1

Rhopaloeides odorabile metagenome replicate 1 (BBAY34) (MER-FS) (*)[F]

Rhopaloeides odorabile metagenome replicate 3

Rhopaloeides odorabile metagenome replicate 3 (BBAY36) (MER-FS) (*)[F]

Unclassified

Gut microbiome of Mt. Pinos Trichonympha termites

Termite Protist Endosymbiont Community (*)[D]

Deepwater Horizon Subsurface Plume Metatranscriptome

16-4 Below Plume (16-4 Below Plume) (*)[D]

16-5 In Plume (16-5 In Plume) (*)[D]

52-1 Below Plume (52-1 Below Plume) (MER-FS) (*)[D]

52-4 In plume (52-4 In Plume) (*)[D]

Guaymas Basin hydrothermal plume

GB plume transcript assembly (MER-FS) (*)[P]

Hydrothermal vent microbial communities from Guaymas and Carmen Basins, Gulf of California, Sample 457 (MER-FS) (*)[D]

Sediment microbial communities from Kolumbo Volcano mats

Marine sediment microbial communities from Kolumbo Volcano mats, Greece, sample red mat (MER-FS) (*)[D]

Marine sediment microbial communities from Kolumbo ... ano mats, Greece, sample red mat (Red mat combined assembly) (MER-FS) (*)[D]

Marine sediment microbial communities from Kolumbo Volcano mats, Greece, sample white/grey mat (MER-FS) (*)[D]

Marine sediment microbial communities from Kolumbo ... te/grey mat (white/grey mat, combined 454/Illumina assembly) (MER-FS) (*)[D]

Marine planktonic communities from Hawaii Ocean Times Series Station (HOT/ALOHA)

1_Upper_euphotic (MER-FS) (*)[D]

2_Base_of_chrolophyll_max (*)[D]

3_Below_base_of_euphotic (MER-FS) (*)[D]

6_Upper_euphotic (MER-FS) (*)[D]

Marine microbial communities from the Eastern Subtropical North Pacific Ocean, Expanding Oxygen minimum zones

Line P August 2008 P26 10m (Line P August 2008 P26 10m, March 2012 Assem) (MER-FS) (*)[D]

Line P August 2009 P16 10m (Line P August 2009 P16 10m, March 2012 Assem) (MER-FS) (*)[D]

Line P June 2009 P12 10m (Line P June 2009 P12 10m, March 2012 Assem) (MER-FS) (*)[D]

Line P June 2009 P4 10m (Line P June 2009 P4 10m, March 2012 Assem) (MER-FS) (*)[D]

Line P sample F_10_SI03_10 (F_10_S103_10) (MER-FS) (*)[D]

Line P sample F_10_SI03_10 (Line P sample_F_10_SI03_10, March 2012 Assem) (MER-FS) (*)[D]

Line P sample F_10_SI03_10 (sample_F_10_SI03_10 June 2011 assem) (MER-FS) (*)[D]

Line P sample_A_09_P04_10 (A_09_P04_10) (MER-FS) (*)[D]

Line P sample_A_09_P04_10 (Line P sample_A_09_P04_10, April 2012 Assem) (MER-FS) (*)[D]

Line P sample_A_09_P04_10 (sample_A_09_P04_10 June 2011 assem) (MER-FS) (*)[D]

Line P sample_F_10_SI03_100 (F_10_SI03_100) (MER-FS) (*)[D]

Line P sample_F_10_SI03_100 (Line P sample_F_10_SI03_100, March 2012 Assem) (MER-FS) (*)[D]

Line P sample_F_10_SI03_100 (sample_F_10_SI03_100 June 2011 assem) (MER-FS) (*)[D]

Line P sample_F_10_SI03_120 (Line P sample_F_10_SI03_120, March 2012 Assem) (MER-FS) (*)[D]

Line P sample_F_10_SI03_120 (sample_F_10_SI03_120 June 2011 assem) (MER-FS) (*)[D]

Line P sample_F_10_SI03_135 (F_10_SI03_135) (MER-FS) (*)[D]

Line P sample_F_10_SI03_135 (Line P sample_F_10_SI03_135, March 2012 Assem) (MER-FS) (*)[D]

Line P sample_F_10_SI03_135 (sample_F_10_SI03_135 June 2011 assem) (MER-FS) (*)[D]

Line P sample_F_10_SI03_150 (Line P sample_F_10_SI03_150, April 2012 Assem) (MER-FS) (*)[D]

Line P sample_F_10_SI03_150 (sample_F_10_SI03_150 June 2011 assem) (MER-FS) (*)[D]

Marine planktonic communities from Hawaii Ocean Times Series Station (HOT/ALOHA)

4_Deep_abyss (MER-FS) (*)[D]

Marine planktonic communities from Hawaii Ocean Times Series Station (HOT/ALOHA)

5_Below_upper_mesopelagic (MER-FS) (*)[D]

7_Oxygen_minimum_layer (MER-FS) (*)[D]

Marine microbial communities from the Eastern Subtropical North Pacific Ocean, Expanding Oxygen minimum zones

Line P August 2008 P12 1000m (Line P August 2008 P12 1000m, June 2012 Assem) (MER-FS) (*)[D]

Line P August 2008 P12 2000m (Line P August 2008 P12 2000m, March 2012 Assem) (MER-FS) (*)[D]

Line P August 2008 P20 2000m (Line P August 2008 P20 2000m, March 2012 Assem) (MER-FS) (*)[D]

Line P August 2008 P20 500m (Line P August 2008 P20 500m, March 2012 Assem) (MER-FS) (*)[D]

Line P August 2008 P26 1000m (Line P August 2008 P26 1000m, March 2012 Assem) (MER-FS) (*)[D]

Line P August 2009 P16 2000m (Line P August 2009 P16 2000m, March 2012 Assem) (MER-FS) (*)[D]

Line P August 2009 P16 500m (Line P August 2009 P16 500m, March 2012 Assem) (MER-FS) (*)[D]

Line P August 2009 P20 2000m (Line P August 2009 P20 2000m, June 2012 Assem) (MER-FS) (*)[D]

Line P August 2009 P26 500m (Line P August 2009 P26 500m, March 2012 Assem) (MER-FS) (*)[D]

Line P February 2009 P12 1000m (Line P February 2009 P12 1000m, March 2012 Assem) (MER-FS) (*)[D]

Line P February 2009 P12 500m (Line P February 2009 P12 500m, March 2012 Assem) (MER-FS) (*)[D]

Line P February 2009 P26 1000m (Line P February 2009 P26 1000m, March 2012 Assem) (MER-FS) (*)[D]

Line P February 2009 P26 500m (Line P February 2009 P26 500m, March 2012 Assem) (MER-FS) (*)[D]

Line P February 2010 P16 1000m (Line P February 2010 P16 1000m, May 2012 Assem) (MER-FS) (*)[D]

Line P February 2010 P16 2000m (Line P February 2010 P16 2000m, April 2012 Assem) (MER-FS) (*)[D]

Line P February 2010 P16 500m (Line P February 2010 P16 500m, March 2012 Assem) (MER-FS) (*)[D]

Line P June 2008 P12 1000m (Line P June 2008 P12 1000m, April 2012 Assem) (MER-FS) (*)[D]

Line P June 2008 P12 2000m (Line P June 2008 P12 2000m, March 2012 Assem) (MER-FS) (*)[D]

Line P June 2008 P12 500m (Line P June 2008 P12 500m, May 2012 Assem) (MER-FS) (*)[D]

Line P June 2008 P16 1000m (Line P June 2008 P16 1000m, March 2012 Assem) (MER-FS) (*)[D]

Line P June 2008 P16 500m (Line P June 2008 P16 500m, March 2012 Assem) (MER-FS) (*)[D]

Line P June 2008 P4 1000m (Line P June 2008 P4 1000m, March 2012 Assem) (MER-FS) (*)[D]

Line P June 2008 P4 1300m (Line P June 2008 P4 1300m, March 2012 Assem) (MER-FS) (*)[D]

Line P June 2008 P4 500m (Line P June 2008 P4 500m, March 2012 Assem) (MER-FS) (*)[D]

Line P June 2009 P12 500m (Line P June 2009 P12 500m, March 2012 Assem) (MER-FS) (*)[D]

Line P June 2009 P16 1000m (Line P June 2009 P16 1000m, March 2012 Assem) (MER-FS) (*)[D]

Line P June 2009 P16 2000m (Line P June 2009 P16 2000m, March 2012 Assem) (MER-FS) (*)[D]

Line P June 2009 P16 500m (Line P June 2009 P16 500m, March 2012 Assem) (MER-FS) (*)[D]

Line P June 2009 P20 2000m (Line P June 2009 P20 2000m, March 2012 Assem) (MER-FS) (*)[D]

Line P sample_A_09_P04_1000 (A_09_P04_1000 June 2011 assem) (MER-FS) (*)[D]

Line P sample_A_09_P04_1000 (Line P sample_A_09_P04_1000, March 2012 Assem) (MER-FS) (*)[D]

Line P sample_A_09_P04_1300 (A_09_P04_1300 June 2011 assembly) (MER-FS) (*)[D]

Line P sample_A_09_P04_1300 (A_09_P04_1300) (MER-FS) (*)[D]

Line P sample_A_09_P04_1300 (Line P sample_A_09_P04_1300, March 2012 Assem) (MER-FS) (*)[D]

Line P sample_A_09_P04_500 (A_09_P04_500) (MER-FS) (*)[D]

Line P sample_A_09_P04_500 (Line P sample_A_09_P04_500, March 2012 Assem) (MER-FS) (*)[D]

Line P sample_A_09_P04_500 (sample_A_09_P04_500 June 2011 assem) (MER-FS) (*)[D]

Line P sample_A_09_P20_1000 (A_09_P20_1000) (MER-FS) (*)[D]

Line P sample_A_09_P20_1000 (Line P sample_A_09_P20_1000, April 2012 Assem) (MER-FS) (*)[D]

Line P sample_A_09_P20_1000 (sample_A_09_P20_1000 June 2011 assem) (MER-FS) (*)[D]

Line P sample_A_09_P20_500 (Line P sample_A_09_P20_500, March 2012 Assem) (MER-FS) (*)[D]

Line P sample_A_09_P20_500 (sample_A_09_P20_500 June 2011 assem) (MER-FS) (*)[D]

Line P sample_F_10_SI03_200 (Line P sample_F_10_SI03_200, March 2012 Assem) (MER-FS) (*)[D]

Line P sample_F_10_SI03_200 (sample_F_10_SI03_200 June 2011 assem) (MER-FS) (*)[D]

Line P sample_J_08_P26_500 (J_08_P26_500) (MER-FS) (*)[D]

Line P sample_J_08_P26_500 (Line P sample_J_08_P26_500, March 2012 Assem) (MER-FS) (*)[D]

Line P sample_J_08_P26_500 (sample_J_08_P26_500 June 2011 assem) (MER-FS) (*)[D]

Line P sample_J_09_P20_1000 (Line P sample_J_09_P20_1000, April 2012 Assem) (MER-FS) (*)[D]

Line P sample_J_09_P20_1000 (sample_J_09_P20_1000 June 2011 assem) (MER-FS) (*)[D]

Line P sample_J_09_P20_500 (Line P sample_J_09_P20_500, March 2012 Assem) (MER-FS) (*)[D]

Line P sample_J_09_P20_500 (sample_J_09_P20_500 June 2011 assem) (MER-FS) (*)[D]

CSP_788545, ANME-1

ANME Fosmids Round 1 (ANME Fosmids Round 1, Sept 2012 Assem) (MER-FS) (*)[D]

Methane oxidizing archaeal communities in the Santa Barbara Basin

Marine sediment archaeal communities from Santa Ba ... t are methane-oxidizing, sample 0-3 cm (ANME Sed A12 0-3 cm) (MER-FS) (*)[D]

Marine sediment archaeal communities from Santa Ba ... e methane-oxidizing, sample 12-15 cm (ANME Sed A12 12-15 cm) (MER-FS) (*)[D]

Marine sediment archaeal communities from Santa Ba ... e methane-oxidizing, sample 15-18 cm (ANME Sed A12 15-18 cm) (MER-FS) (*)[D]

Marine sediment archaeal communities from Santa Ba ... t are methane-oxidizing, sample 3-6 cm (ANME Sed A12 3-6 cm) (MER-FS) (*)[D]

Marine sediment archaeal communities from Santa Ba ... t are methane-oxidizing, sample 6-9 cm (ANME Sed A12 6-9 cm) (MER-FS) (*)[D]

Marine sediment archaeal communities from Santa Ba ... are methane-oxidizing, sample 9-12 cm (ANME Sed A12 9-12 cm) (MER-FS) (*)[D]

Deep ocean microbial communities from the Global Malaspina Expedition

Deep seawater metagenome MMD0.2 (Deep seawater metagenome MMD0.2, ASSEMBLY_DATE=20130220) (MER-FS) (*)[D]

Deep seawater metagenome MMD3.0 (Deep seawater metagenome MMD3.0, ASSEMBLY_DATE=20130213) (MER-FS) (*)[D]

Deep seawater metagenome MP0103 (Deep seawater metagenome MP0103, ASSEMBLY_DATE=20130220) (MER-FS) (*)[D]

Deep seawater metagenome MP0104 (Deep seawater metagenome MP0104, ASSEMBLY_DATE=20130220) (MER-FS) (*)[D]

Deep seawater metagenome MP0144 (Deep seawater metagenome MP0144, ASSEMBLY_DATE=20130213) (MER-FS) (*)[D]

Deep seawater metagenome MP0145 (Deep seawater metagenome MP0145, ASSEMBLY_DATE=20130213) (MER-FS) (*)[D]

Deep seawater metagenome MP0203 (Deep seawater metagenome MP0203, ASSEMBLY_DATE=20130221) (MER-FS) (*)[D]

Deep seawater metagenome MP0204 (Deep seawater metagenome MP0204, ASSEMBLY_DATE=20130220) (MER-FS) (*)[D]

Deep seawater metagenome MP0261 (Deep seawater metagenome MP0261, ASSEMBLY_DATE=20130213) (MER-FS) (*)[D]

Deep seawater metagenome MP0262 (Deep seawater metagenome MP0262, ASSEMBLY_DATE=20130213) (MER-FS) (*)[D]

Deep seawater metagenome MP0326 (Deep seawater metagenome MP0326, ASSEMBLY_DATE=20130213) (MER-FS) (*)[D]

Deep seawater metagenome MP0327 (Deep seawater metagenome MP0327, ASSEMBLY_DATE=20130221) (MER-FS) (*)[D]

Deep seawater metagenome MP0371 (Deep seawater metagenome MP0371, ASSEMBLY_DATE=20130213) (MER-FS) (*)[D]

Deep seawater metagenome MP0372 (Deep seawater metagenome MP0372, ASSEMBLY_DATE=20130221) (MER-FS) (*)[D]

Deep seawater metagenome MP0440 (Deep seawater metagenome MP0440, ASSEMBLY_DATE=20130213) (MER-FS) (*)[D]

Deep seawater metagenome MP0441 (Deep seawater metagenome MP0441, ASSEMBLY_DATE=20130213) (MER-FS) (*)[D]

Deep seawater metagenome MP0555 (Deep seawater metagenome MP0555, ASSEMBLY_DATE=20130213) (MER-FS) (*)[D]

Deep seawater metagenome MP0556 (Deep seawater metagenome MP0556, ASSEMBLY_DATE=20130220) (MER-FS) (*)[D]

Deep seawater metagenome MP0626 (Deep seawater metagenome MP0626, ASSEMBLY_DATE=20130213) (MER-FS) (*)[D]

Deep seawater metagenome MP0627 (Deep seawater metagenome MP0627, ASSEMBLY_DATE=20130213) (MER-FS) (*)[D]

Deep seawater metagenome MP0739 (Deep seawater metagenome MP0739, ASSEMBLY_DATE=20130213) (MER-FS) (*)[D]

Deep seawater metagenome MP0740 (Deep seawater metagenome MP0740, ASSEMBLY_DATE=20130213) (MER-FS) (*)[D]

Deep seawater metagenome MP0758 (Deep seawater metagenome MP0758, ASSEMBLY_DATE=20130213) (MER-FS) (*)[D]

Deep seawater metagenome MP0759 (Deep seawater metagenome MP0759, ASSEMBLY_DATE=20130213) (MER-FS) (*)[D]

Deep seawater metagenome MP0900 (Deep seawater metagenome MP0900, ASSEMBLY_DATE=20130213) (MER-FS) (*)[D]

Deep seawater metagenome MP0901 (Deep seawater metagenome MP0901, ASSEMBLY_DATE=20130221) (MER-FS) (*)[D]

Deep seawater metagenome MP0959 (Deep seawater metagenome MP0959, ASSEMBLY_DATE=20130213) (MER-FS) (*)[D]

Deep seawater metagenome MP0960 (Deep seawater metagenome MP0960, ASSEMBLY_DATE=20130213) (MER-FS) (*)[D]

Deep seawater metagenome MP1091 (Deep seawater metagenome MP1091, ASSEMBLY_DATE=20130213) (MER-FS) (*)[D]

Deep seawater metagenome MP1092 (Deep seawater metagenome MP1092, ASSEMBLY_DATE=20130213) (MER-FS) (*)[D]

Deep seawater metagenome MP1140 (Deep seawater metagenome MP1140, ASSEMBLY_DATE=20130213) (MER-FS) (*)[D]

Deep seawater metagenome MP1141 (Deep seawater metagenome MP1141, ASSEMBLY_DATE=20130221) (MER-FS) (*)[D]

Deep seawater metagenome MP1201 (Deep seawater metagenome MP1201, ASSEMBLY_DATE=20130213) (MER-FS) (*)[D]

Deep seawater metagenome MP1202 (Deep seawater metagenome MP1202, ASSEMBLY_DATE=20130220) (MER-FS) (*)[D]

Deep seawater metagenome MP1241 (Deep seawater metagenome MP1241, ASSEMBLY_DATE=20130213) (MER-FS) (*)[D]

Deep seawater metagenome MP1374 (Deep seawater metagenome MP1374, ASSEMBLY_DATE=20130213) (MER-FS) (*)[D]

Deep seawater metagenome MP1434 (Deep seawater metagenome MP1434, ASSEMBLY_DATE=20130213) (MER-FS) (*)[D]

Deep seawater metagenome MP1482 (Deep seawater metagenome MP1482, ASSEMBLY_DATE=20130213) (MER-FS) (*)[D]

Deep seawater metagenome MP1483 (Deep seawater metagenome MP1483, ASSEMBLY_DATE=20130213) (MER-FS) (*)[D]

Deep seawater metagenome MP1493 (Deep seawater metagenome MP1493, ASSEMBLY_DATE=20130213) (MER-FS) (*)[D]

Deep seawater metagenome MP1494 (Deep seawater metagenome MP1494, ASSEMBLY_DATE=20130213) (MER-FS) (*)[D]

Deep seawater metagenome MP1648 (Deep seawater metagenome MP1648, ASSEMBLY_DATE=20130213) (MER-FS) (*)[D]

Deep seawater metagenome MP1649 (Deep seawater metagenome MP1649, ASSEMBLY_DATE=20130213) (MER-FS) (*)[D]

Deep seawater metagenome MP1787 (Deep seawater metagenome MP1787, ASSEMBLY_DATE=20130213) (MER-FS) (*)[D]

Deep seawater metagenome MP1788 (Deep seawater metagenome MP1788, ASSEMBLY_DATE=20130213) (MER-FS) (*)[D]

Deep seawater metagenome MP1896 (Deep seawater metagenome MP1896, ASSEMBLY_DATE=20130221) (MER-FS) (*)[D]

Deep seawater metagenome MP2016 (Deep seawater metagenome MP2016, ASSEMBLY_DATE=20130213) (MER-FS) (*)[D]

Deep seawater metagenome MP2052 (Deep seawater metagenome MP2052, ASSEMBLY_DATE=20130213) (MER-FS) (*)[D]

Deep seawater metagenome MP2053 (Deep seawater metagenome MP2053, ASSEMBLY_DATE=20130213) (MER-FS) (*)[D]

Deep seawater metagenome MP2097 (Deep seawater metagenome MP2097, ASSEMBLY_DATE=20130213) (MER-FS) (*)[D]

Deep seawater metagenome MP2098 (Deep seawater metagenome MP2098, ASSEMBLY_DATE=20130221) (MER-FS) (*)[D]

Deep seawater metagenome MP2158 (Deep seawater metagenome MP2158, ASSEMBLY_DATE=20130220) (MER-FS) (*)[D]

Deep seawater metagenome MP2252 (Deep seawater metagenome MP2252, ASSEMBLY_DATE=20130221) (MER-FS) (*)[D]

Deep seawater metagenome MP2253 (Deep seawater metagenome MP2253, ASSEMBLY_DATE=20130221) (MER-FS) (*)[D]

Deep seawater metagenome MP2497 (Deep seawater metagenome MP2497, ASSEMBLY_DATE=20130213) (MER-FS) (*)[D]

Deep seawater metagenome MP2633 (Deep seawater metagenome MP2633, ASSEMBLY_DATE=20130221) (MER-FS) (*)[D]

Deep seawater metagenome MP2634 (Deep seawater metagenome MP2634, ASSEMBLY_DATE=20130213) (MER-FS) (*)[D]

Deep seawater metagenome MP2913 (Deep seawater metagenome MP2913, ASSEMBLY_DATE=20130213) (MER-FS) (*)[D]

Deep seawater metagenome MP2914 (Deep seawater metagenome MP2914, ASSEMBLY_DATE=20130213) (MER-FS) (*)[D]

Deep seawater metagenome MP2968 (Deep seawater metagenome MP2968, ASSEMBLY_DATE=20130213) (MER-FS) (*)[D]

Deep seawater metagenome MP2969 (Deep seawater metagenome MP2969, ASSEMBLY_DATE=20130221) (MER-FS) (*)[D]

Marine viral communities from the Subarctic Pacific Ocean

Line P viral metagenome LP-28 (Line P viral metagenome LP-28, ASSEMBLY_DATE=20130529) (MER-FS) (*)[D]

Line P viral metagenome LP-32 (Line P viral metagenome LP-32, ASSEMBLY_DATE=20130607) (MER-FS) (*)[D]

Line P viral metagenome LP-53 (Line P viral metagenome LP-53, ASSEMBLY_DATE=20130525) (MER-FS) (*)[D]

Line P viral metagenome LP-53 (Line P viral metagenome LP-53, ASSEMBLY_DATE=20130525) (MER-FS) (*)[D]

Marine Trichodesmium cyanobacterial communities from the Bermuda Atlantic Time-Series

Marine Trichodesmium cyanobacterial communities from the Bermuda Atlantic Time-Series (MER-FS) (*)[D]

Marine Trichodesmium cyanobacterial communities from the Nor ... l Gyre outside Oahu, HI, sample from new species B colonies (*)[D]

Marine microbial communities from Delaware Coast

Marine microbial communities from Delaware Coast, sample from Delaware MO Early Summer May 2010 (MER-FS) (*)[D]

Marine microbial communities from Delaware Coast, ... May 2010 (Delaware MO Early Summer May 2010, Feb 2012 assem) (MER-FS) (*)[D]

Sediment archaeal communities from Eel River Basin

Anaerobic methane oxidation (AOM) community from Eel River Basin sediment, California (*)[D]

Coastal water and sediment microbial communities from Arctic

Sediment microbial communities from Arctic Ocean, ... 12-225-485cm (High methane PC12-225-485cm Dec 2010 assembly) (MER-FS) (*)[D]

Sediment microbial communities from Arctic Ocean, ... 12-225-485cm (High methane PC12-225-485cm Jan 2011 assembly) (MER-FS) (*)[D]

COGITO (Coastal Microbe Genomic & Taxonomic Observatory)

COGITO 998_met_01 (COGITO 998_met_01, ASSEMBLY_DATE=20130420) (MER-FS) (*)[D]

COGITO 998_met_02 (COGITO 998_met_02, ASSEMBLY_DATE=20130419) (MER-FS) (*)[D]

COGITO 998_met_05 (COGITO 998_met_05, ASSEMBLY_DATE=20130426) (MER-FS) (*)[D]

COGITO 998_met_10 (COGITO 998_met_10, ASSEMBLY_DATE=20130417) (MER-FS) (*)[D]

Marine microbial communities from Delaware Coast

Late spring/early summer (stable) metatranscriptome (MER-FS) (*)[D]

Marine microbial communities from Delaware Coast, ... g March 2010 (Delaware MO Spring March 2010, Nov 2011 assem) (MER-FS) (*)[D]

Marine microbial communities from Delaware Coast, ... mer July 2011 (Delaware MO Summer July 2011, Nov 2011 assem) (MER-FS) (*)[D]

Marine microbial communities from Delaware Coast, sample from Delaware MO Winter December 2010 (MER-FS) (*)[D]

Marine microbial communities from Delaware Coast, ... mber 2010 (Delaware MO Winter December 2010, Nov 2011 assem) (MER-FS) (*)[D]

Marine Bacterioplankton communities from Antarctic

Marine Bacterioplankton communities from Antarctic, Sample 10335 (Summer fosmids) (*)[D]

Marine Bacterioplankton communities from Antarctic, Sample 10334 (Winter fosmids) (*)[D]

Marine Bacterioplankton communities from the Antarctic

Marine Bacterioplankton communities from the Antarctic, sample from Summer (*)[P]

Marine Bacterioplankton communities from the Antarctic, sample from Summer (Summer fosmids Sept 2010 assemblies) (*)[D]

Marine Bacterioplankton communities from the Antarctic, sample from Winter (Winter fosmids Sept 2010 assemblies) (*)[D]

Olavius algarvensis microbiome from Mediterranean sea

Olavius algarvensis endosymbiont metagenome Delta1 (MER-FS) (*)[D]

Olavius algarvensis endosymbiont metagenome Delta4 (*)[D]

Olavius algarvensis endosymbiont metagenome Gamma1 (*)[D]

Olavius algarvensis endosymbiont metagenome Gamma3 (*)[D]

Coastal water and sediment microbial communities from Arctic

Sediment microbial communities from Arctic Ocean, ... m high methane PC12-236-260cm (High methane PC12-236-260cm) (MER-FS) (*)[D]

Sediment microbial communities from Arctic Ocean, ... PC12-244-90cm (Low methane PC12-244-90cm Sept2010 assembly) (MER-FS) (*)[D]

Sediment microbial communities from Arctic Ocean, ... e from low methane PC12-247-20cm (Low methane PC12-247-20cm) (MER-FS) (*)[D]

Sediment microbial communities from Arctic Ocean, ... -240-170cm (Medium methane PC12-240-170cm Sept2010 assembly) (MER-FS) (*)[D]

COGITO (Coastal Microbe Genomic & Taxonomic Observatory)

COGITO 998_met_03 (COGITO 998_met_03, ASSEMBLY_DATE=20130417) (MER-FS) (*)[D]

COGITO 998_met_03 (COGITO 998_met_03, ASSEMBLY_DATE=20130417) (MER-FS) (*)[D]

COGITO 998_met_04 (COGITO 998_met_04, ASSEMBLY_DATE=20130417) (MER-FS) (*)[D]

COGITO 998_met_06 (COGITO 998_met_06, ASSEMBLY_DATE=20130420) (MER-FS) (*)[D]

COGITO 998_met_07 (COGITO 998_met_07, ASSEMBLY_DATE=20130417) (MER-FS) (*)[D]

COGITO 998_met_08 (COGITO 998_met_08, ASSEMBLY_DATE=20130417) (MER-FS) (*)[D]

COGITO 998_met_09 (COGITO 998_met_09, ASSEMBLY_DATE=20130423) (MER-FS) (*)[D]

Marine Bacterioplankton communities from Antarctic

Marine Bacterioplankton communities from Antarctic, sample from Summer (Summer fosmid end sequences) (MER-FS) (*)[D]

Marine Bacterioplankton communities from Antarctic, sample from Winter (Winter fosmid end sequences) (MER-FS) (*)[D]

Mangrove

BB Mangrove A Sediment - Bioluminescent Bay in La ... id - Bioluminescent Bay in La Paraguera,PR, July 2012 Assem) (MER-FS) (*)[D]

BB Mangrove B Liquid - Bioluminescent Bay in La Pa ... uid - Bioluminescent Bay in La ParagueraPR, July 2012 Assem) (MER-FS) (*)[D]

Marine microbial communities from chronically polluted sediments in four geographic locations

Baltic Sea site KBA sample SWE 02_21m (Baltic Sea site KBA sample SWE 02_21m, Feb 2012 Assem) (MER-FS) (*)[D]

King George Island site S1 sample ANT 01_9.5m (King George Island site S1 sample ANT 01_9.5m, Oct 2011 Assem) (MER-FS) (*)[D]

King George Island site S1 sample ANT 02_9.5m (King George Island site S1 sample ANT 02_9.5m, Dec 2011 Assem) (MER-FS) (*)[D]

King George Island site S1 sample ANT 03_9.5m (King George Island site S1 sample ANT 03_9.5m, Dec 2011 Assem) (MER-FS) (*)[D]

King George Island site S2 sample ANT 04_23.45m (K ... George Island site S2 sample ANT 04_23.45m, Dec 2011 Assem) (MER-FS) (*)[D]

King George Island site S2 sample ANT 05_23.45m (K ... George Island site S2 sample ANT 05_23.45m, Jan 2012 Assem) (MER-FS) (*)[D]

King George Island site S2 sample ANT 06_23.45m (K ... George Island site S2 sample ANT 06_23.45m, Oct 2011 Assem) (MER-FS) (*)[D]

Svalbard Archipelago station 1 sample NOR 02_45m (... ard Archipelago station 1 sample NOR 02_45m, Jan 2012 Assem) (MER-FS) (*)[D]

Svalbard Archipelago station 1 sample NOR 05_45m (... ard Archipelago station 1 sample NOR 05_45m, Nov 2011 Assem) (MER-FS) (*)[D]

Svalbard Archipelago station 1 sample NOR 08_45m (... ard Archipelago station 1 sample NOR 08_45m, Dec 2011 Assem) (MER-FS) (*)[D]

Svalbard Archipelago station 2 sample NOR 13_50m (... ard Archipelago station 2 sample NOR 13_50m, Oct 2011 Assem) (MER-FS) (*)[D]

Svalbard Archipelago station 2 sample NOR 15_50m (... ard Archipelago station 2 sample NOR 15_50m, Dec 2011 Assem) (MER-FS) (*)[D]

Svalbard Archipelago station 2 sample NOR 18_50m (... ard Archipelago station 2 sample NOR 18_50m, Dec 2011 Assem) (MER-FS) (*)[D]

Tierra del Fuego site MC sample ARG 01_11.3m (Tierra del Fuego site MC sample ARG 01_11.3m, Nov 2011 Assem) (MER-FS) (*)[D]

Tierra del Fuego site MC sample ARG 02_11.3m (Tierra del Fuego site MC sample ARG 02_11.3m, Jan 2012 Assem) (MER-FS) (*)[D]

Tierra del Fuego site MC sample ARG 03_11.3m (Tierra del Fuego site MC sample ARG 03_11.3m, Oct 2011 Assem) (MER-FS) (*)[D]

Tierra del Fuego site OR sample ARG 04_12.3m (Tierra del Fuego site OR sample ARG 04_12.3m, Oct 2011 Assem) (MER-FS) (*)[D]

Tierra del Fuego site OR sample ARG 06_12.3m (Tierra del Fuego site OR sample ARG 06_12.3m, Oct 2011 Assem) (MER-FS) (*)[D]

Halophilic archaeon cultivated from rock salt

Halophilic archaeon cultivated from rock salt, sample 1 (AchaeonTV9) (MER-FS) (*)[D]

Halophilic bacterium cultivated from rock salt

Halophilic bacterium cultivated from rock salt, sample 2 (Halophilic bacterium TV14) (MER-FS) (*)[D]

Marine subseafloor sediment microbial communities from Peru Margin, Ocean Drilling Program Site 1229

Marine subseafloor sediment microbial communities, ... uary, NC, USA 14E (White Oak River Estuary June 2011 assem) (MER-FS) (*)[D]

PR Tt Sediment 1 - Bioluminescent Bay in La Paragu ... t 1 - Bioluminescent Bay in La ParagueraPR, July 2012 Assem) (MER-FS) (*)[D]

PR Tt Sediment 2 - Bioluminescent Bay in La Paragu ... t 2 - Bioluminescent Bay in La ParagueraPR, July 2012 Assem) (MER-FS) (*)[D]

Hypersaline water microbial communities from Lyngbya mats, Guerrero Negro, Mexico and Elkhorn Slough mats, California, USA

Elkhorn Slough mat CD6A Metagenome (Elkhorn Slough mat CD6A, June 2012 Assem) (MER-FS) (*)[D]

Estuarine microbial mat communities from Elkhorn Slough, Moss Landing, CA, that are H2-evolving and photosynthetic

Elkhorn Slough mat CR1B Metatranscriptome (MER-FS) (*)[D]

Elkhorn Slough mat CR2B Metatranscriptome (MER-FS) (*)[D]

Elkhorn Slough mat CR3A Metatranscriptome (MER-FS) (*)[D]

Elkhorn Slough mat CR3B Metatranscriptome (MER-FS) (*)[D]

Elkhorn Slough mat CR5A Metatranscriptome (MER-FS) (*)[D]

Elkhorn Slough mat CR5B Metatranscriptome (MER-FS) (*)[D]

Hypersaline water microbial communities from Lyngbya mats, Guerrero Negro, Mexico and Elkhorn Slough mats, California, USA

Elkhorn Slough mat CR6B Metatranscriptome (MER-FS) (*)[D]

Elkhorn Slough mat MD2A Metagenome (Elkhorn Slough mat MD2A Metagenome, ASSEMBLY_DATE=20121222) (MER-FS) (*)[D]

Elkhorn Slough mat MD2A Metagenome (Elkhorn Slough mat MD2A Metagenome, ASSEMBLY_DATE=20130423) (MER-FS) (*)[D]

Elkhorn Slough mat MR6A Metatranscriptome (MER-FS) (*)[D]

Water and sediment microbiomes of the Columbia River coastal margin, including river, estuary, plume and ocean

Estuarine microbial communities from Columbia River, sample from South Channel ETM site, GS313-0p1-ETM-15m (MER-FS) (*)[D]

Estuarine microbial communities from Columbia River, sample from South Channel ETM site, GS313-3LG-ETM-15m (MER-FS) (*)[D]

Marine microbial communities from chronically polluted sediments in four geographic locations

Tierra del Fuego site OR sample ARG 05_12.3m (Tierra del Fuego site OR sample ARG 05_12.3m, Oct 2011 Assem) (MER-FS) (*)[D]

Water and sediment microbiomes of the Columbia River coastal margin, including river, estuary, plume and ocean

Estuarine microbial communities from Columbia River, sample from South Channel ETM site, GS313-0p8-ETM-15m (MER-FS) (*)[D]

Marine microbial communities from Columbia River, CM, sample from CR-7km from mouth, GS312-0p1-CR7-chlmax (MER-FS) (*)[D]

Marine microbial communities from Columbia River, CM, sample from CR-7km from mouth, GS312-0p8-CR7-chlmax (MER-FS) (*)[D]

Marine microbial communities from Columbia River, CM, sample from CR-7km from mouth, GS312-3p0-CR7-chlmax (MER-FS) (*)[D]

Marine microbial communities from Columbia River, CM, sample from Cape Meares, GS311-0p1-Deep1200 (MER-FS) (*)[D]

Marine microbial communities from Columbia River, CM, sample from Cape Meares, GS311-0p8-Deep1200 (MER-FS) (*)[D]

Marine microbial communities from Columbia River, CM, sample from Cape Meares, GS311-3LG-Deep1200 (MER-FS) (*)[D]

Marine microbial communities from Columbia River, CM, sample from Newport Hydroline, GS310-0p1-Hyp-75m (MER-FS) (*)[D]

Marine microbial communities from Columbia River, CM, sample from Newport Hydroline, GS310-0p8-Hyp-75m (MER-FS) (*)[D]

Marine microbial communities from Columbia River, CM, sample from Newport Hydroline, GS310-3LG-Hyp-75m (MER-FS) (*)[D]

Pond

PR CR 10% Liquid 1 Cabo Rojo, PR (PR CR 10% Liquid 1 Cabo Rojo PR, June 2012 Assem) (MER-FS) (*)[D]

PR CR 10% Liquid 3 Cabo Rojo, PR (PR CR 10% Liquid 3 Cabo Rojo PR, June 2012 Assem) (MER-FS) (*)[D]

Pond 1C Liquid 1 Union City, CA (Pond 1C Liquid 1 Union City, June 2012 Assem) (MER-FS) (*)[D]

Pond 1C Liquid 2 Union City, CA (Pond 1C Liquid 2 Union City, July 2012 Assem) (MER-FS) (*)[D]

Pond 1C Liquid 3 Union City, CA (Pond 1C Liquid 3 Union City, June 2012 Assem) (MER-FS) (*)[D]

Pond 1C Sediment 2 Union City, CA (Pond 1C Sediment 2 Union City, June 2012 Assem) (MER-FS) (*)[D]

Pond 1C Sediment 3 Union City, CA (Pond 1C Sediment 3 Union City, June 2012 Assem) (MER-FS) (*)[D]

Pond 2C Liquid 1 Union City, CA (Pond 2C Liquid 1 Union City, June 2012 Assem) (MER-FS) (*)[D]

Pond 2C Liquid 2 Union City, CA (Pond 2C Liquid 2 Union City, July 2012 Assem) (MER-FS) (*)[D]

Pond 2C Liquid 3 Union City, CA (Pond 2C Liquid 3 Union City, June 2012 Assem) (MER-FS) (*)[D]

Pond 2C Sediment 1 Union City, CA (Pond 2C Sediment 1 Union City, June 2012 Assem) (MER-FS) (*)[D]

Pond 2C Sediment 3 Union City, CA (Pond 2C Sediment 3 Union City, June 2012 Assem) (MER-FS) (*)[D]

Pond A23 Liquid 1 Fremont,CA (Pond A23 Liquid 1 Fremont, June 2012 Assem) (MER-FS) (*)[D]

Pond A23 Liquid 2 Fremont,CA (Pond A23 Liquid 2 Fremont, July 2012 Assem) (MER-FS) (*)[D]

Pond A23 Liquid 3 Fremont,CA (Pond A23 Liquid 3 Fremont, July 2012 Assem) (MER-FS) (*)[D]

Marine microbial communities from the Eastern Subtropical North Pacific Ocean, Expanding Oxygen minimum zones

Saanich Inlet 34 06/16/09 100m (Saanich Inlet 34 06/16/09 100m, March 2012 Assem) (MER-FS) (*)[D]

Saanich Inlet 34 06/16/09 10m (Saanich Inlet 34 06/16/09 10m, March 2012 Assem) (MER-FS) (*)[D]

Saanich Inlet 34 06/16/09 120m (Saanich Inlet 34 06/16/09 120m, March 2012 Assem) (MER-FS) (*)[D]

Saanich Inlet 34 06/16/09 135m (Saanich Inlet 34 06/16/09 135m, March 2012 Assem) (MER-FS) (*)[D]

Saanich Inlet 34 06/16/09 150m (Saanich Inlet 34 06/16/09 150m, March 2012 Assem) (MER-FS) (*)[D]

Saanich Inlet 34 06/16/09 200m (Saanich Inlet 34 06/16/09 200m, May 2012 Assem) (MER-FS) (*)[D]

Saanich Inlet 36 08/11/09 100m (Saanich Inlet 36 08/11/09 100m, March 2012 Assem) (MER-FS) (*)[D]

Saanich Inlet 36 08/11/09 120m (Saanich Inlet 36 08/11/09 120m, March 2012 Assem) (MER-FS) (*)[D]

Saanich Inlet 36 08/11/09 135m (Saanich Inlet 36 08/11/09 135m, March 2012 Assem) (MER-FS) (*)[D]

Saanich Inlet 36 08/11/09 150m (Saanich Inlet 36 08/11/09 150m, March 2012 Assem) (MER-FS) (*)[D]

Saanich Inlet 36 08/11/09 200m (Saanich Inlet 36 08/11/09 200m, March 2012 Assem) (MER-FS) (*)[D]

Saanich Inlet 39 11/10/09 100m (Saanich Inlet 39 11/10/09 100m, June 2012 Assem) (MER-FS) (*)[D]

Saanich Inlet 39 11/10/09 10m (Saanich Inlet 39 11/10/09 10m, March 2012 Assem) (MER-FS) (*)[D]

Saanich Inlet 39 11/10/09 120m (Saanich Inlet 39 11/10/09 120m, March 2012 Assem) (MER-FS) (*)[D]

Saanich Inlet 39 11/10/09 135m (Saanich Inlet 39 11/10/09 135m, April 2012 Assem) (MER-FS) (*)[D]

Saanich Inlet 39 11/10/09 150m (Saanich Inlet 39 11/10/09 150m, March 2012 Assem) (MER-FS) (*)[D]

Saanich Inlet 39 11/10/09 200m (Saanich Inlet 39 11/10/09 200m, May 2012 Assem) (MER-FS) (*)[D]

Saanich Inlet 47 07/07/10 100m (Saanich Inlet 47 07/07/10 100m, March 2012 Assem) (MER-FS) (*)[D]

Saanich Inlet 47 07/07/10 120m (Saanich Inlet 47 07/07/10 120m, March 2012 Assem) (MER-FS) (*)[D]

Saanich Inlet 47 07/07/10 135m (Saanich Inlet 47 07/07/10 135m, March 2012 Assem) (MER-FS) (*)[D]

Saanich Inlet 47 07/07/10 150m (Saanich Inlet 47 07/07/10 150m, March 2012 Assem) (MER-FS) (*)[D]

Saanich Inlet 47 07/07/10 200m (Saanich Inlet 47 07/07/10 200m, March 2012 Assem) (MER-FS) (*)[D]

Saanich Inlet 48 08/11/10 100m (Saanich Inlet 48 08/11/10 100m, June 2012 Assem) (MER-FS) (*)[D]

Saanich Inlet 48 08/11/10 10m (Saanich Inlet 48 08/11/10 10m, March 2012 Assem) (MER-FS) (*)[D]

Saanich Inlet 48 08/11/10 120m (Saanich Inlet 48 08/11/10 120m, March 2012 Assem) (MER-FS) (*)[D]

Saanich Inlet 48 08/11/10 135m (Saanich Inlet 48 08/11/10 135m, March 2012 Assem) (MER-FS) (*)[D]

Saanich Inlet 48 08/11/10 150m (Saanich Inlet 48 08/11/10 150m, March 2012 Assem) (MER-FS) (*)[D]

Saanich Inlet 48 08/11/10 200m (Saanich Inlet 48 08/11/10 200m, April 2012 Assem) (MER-FS) (*)[D]

Saanich Inlet 53 01/11/11 100m (Saanich Inlet 53 01/11/11 100m, March 2012 Assem) (MER-FS) (*)[D]

Saanich Inlet 53 01/11/11 10m (Saanich Inlet 53 01/11/11 10m, March 2012 Assem) (MER-FS) (*)[D]

Saanich Inlet 53 01/11/11 120m (Saanich Inlet 53 01/11/11 120m, March 2012 Assem) (MER-FS) (*)[D]

Saanich Inlet 53 01/11/11 135m (Saanich Inlet 53 01/11/11 135m, March 2012 Assem) (MER-FS) (*)[D]

Saanich Inlet 53 01/11/11 150m (Saanich Inlet 53 01/11/11 150m, March 2012 Assem) (MER-FS) (*)[D]

Saanich Inlet 53 01/11/11 200m (Saanich Inlet 53 01/11/11 200m, March 2012 Assem) (MER-FS) (*)[D]

Saanich Inlet 54 02/08/11 100m (Saanich Inlet 54 02/08/11 100m, March 2012 Assem) (MER-FS) (*)[D]

Saanich Inlet 54 02/08/11 120m (Saanich Inlet 54 02/08/11 120m, March 2012 Assem) (MER-FS) (*)[D]

Saanich Inlet 54 02/08/11 135m (Saanich Inlet 54 02/08/11 135m, March 2012 Assem) (MER-FS) (*)[D]

Saanich Inlet 54 02/08/11 150m (Saanich Inlet 54 02/08/11 150m, April 2012 Assem) (MER-FS) (*)[D]

Saanich Inlet 54 02/08/11 200m (Saanich Inlet 54 02/08/11 200m, March 2012 Assem) (MER-FS) (*)[D]

Saanich Inlet 60 08/10/11 100m (Saanich Inlet 60 08/10/11 100m, March 2012 Assem) (MER-FS) (*)[D]

Saanich Inlet 60 08/10/11 150m (Saanich Inlet 60 08/10/11 150m, March 2012 Assem) (MER-FS) (*)[D]

Saanich Inlet 60 08/10/11 200m (Saanich Inlet 60 08/10/11 200m, April 2012 Assem) (MER-FS) (*)[D]

Marine photosynthetic community that grows at 940nm

Microbial Communities from Little Sippewissett Sal ... etic community that grows at 940nm (Marine_940nm_cellulose) (MER-FS) (*)[D]

Marine photosynthetic community that grows at 940nm with malate

Microbial Communities from Little Sippewissett Sal ... unity that grows at 940nm with malate (Marine_940nm_malate) (MER-FS) (*)[D]

Photosynthetic Consortia 590nm sample 1

Microbial Communities from Little Sippewissett Sal ... grown using light of 590nm sample 1 (Marine_590nm_sample1) (MER-FS) (*)[D]

Photosynthetic Consortia 590nm sample 2

Microbial Communities from Little Sippewissett Sal ... grown using light of 590nm sample 2 (Marine_590nm_sample2) (MER-FS) (*)[D]

Photosynthetic Consortia grown using light of 750nm

Microbial Communities from Little Sippewissett Sal ... nthetic Consortia grown using light of 750nm (Marine_750nm) (MER-FS) (*)[D]

Marine photosynthetic community that grows at 740nm

Microbial Communities from Little Sippewissett Sal ... thetic, sample photosynthetic consortia 740nm (Marine_740nm) (MER-FS) (*)[D]

Coral reef

Rhopaloeides odorabile metagenome replicate 2

Rhopaloeides odorabile metagenome replicate 2 (BBAY35) (MER-FS) (*)[F]

unclassified

Marine microbial communities from Near-Shore Anoxic Basin of Saanich Inlet of Vancouver

Saanich Inlet (MER-FS) (*)[D]

Sediment

Marine microbial communities from chronically polluted sediments in four geographic locations

Baltic Sea site KBA sample SWE 07_21m (Baltic Sea site KBA sample SWE 07_21m, Oct 2011 Assem) (MER-FS) (*)[D]

Baltic Sea site KBA sample SWE 12_21m (Baltic Sea site KBA sample SWE 12_21m, Oct 2011 Assem) (MER-FS) (*)[D]

Baltic Sea site KBB sample SWE 21_20.5m (Baltic Sea site KBB sample SWE 21_20.5m, Oct 2011 Assem) (MER-FS) (*)[D]

Baltic Sea site KBB sample SWE 26_20.5m (Baltic Sea site KBB sample SWE 26_20.5m, Nov 2011 Assem) (MER-FS) (*)[D]

Microbial community impact on carbon sequestration in managed wetland ?carbon farming?

Wetland Surface Sediment Aug2011 Site A1 Bulk Meta ... ent Aug2011 Site A1 Bulk Metagenome, ASSEMBLY_DATE=20130408) (MER-FS) (*)[D]

Wetland Surface Sediment Aug2011 Site B2 Bulk Meta ... ent Aug2011 Site B2 Bulk Metagenome, ASSEMBLY_DATE=20130408) (MER-FS) (*)[D]

Soil microbial communities from Twitchell Island in the Sacramento Delta

Wetland microbial communities from Twitchell Islan ... Sediment Feb2011 Site B2 Cattail, Assem Ctgs Oct 2011 assem) (MER-FS) (*)[D]

Wetland microbial communities from Twitchell Islan ... nd Surface Sediment Feb2011 Site B2 Cattail, Oct 2011 assem) (MER-FS) (*)[D]

Wetland microbial communities from Twitchell Islan ... tland Surface Sediment Feb2011 Site A1 Bulk, 2011 Sep Assem) (MER-FS) (*)[D]

Wetland microbial communities from Twitchell Islan ... ent Feb2011 Site A1 Bulk, Asm Ctgs IBYY,IIYG 2012 Mar Assem) (MER-FS) (*)[D]

Wetland microbial communities from Twitchell Islan ... diment Feb2011 Site A1 Bulk, Assem Ctgs IBYY 2011 Sep Assem) (MER-FS) (*)[D]

Wetland microbial communities from Twitchell Islan ... ment Feb2011 Site A1 Bulk, Assem Ctgs IIYG 2012 March Assem) (MER-FS) (*)[D]

Wetland microbial communities from Twitchell Islan ... ent Feb2011 Site A1 Bulk, Ctgs/Rds IBYY,IIYG 2012 Mar Assem) (MER-FS) (*)[D]

Wetland microbial communities from Twitchell Islan ... ment Feb2011 Site A1 Bulk, Ctgs/UnRds IIYG 2012 March Assem) (MER-FS) (*)[D]

Wetland microbial communities from Twitchell Islan ... e Sediment Feb2011 Site A1 Bulk-1% Merged Rds,0.25kb Insert) (MER-FS) (*)[D]

Wetland microbial communities from Twitchell Islan ... e Sediment Feb2011 Site A1 Bulk-1% Merged Rds,0.27kb Insert) (MER-FS) (*)[D]

Wetland microbial communities from Twitchell Islan ... etland Surface Sediment Feb2011 Site A1 Tule Jan 2012 assem) (MER-FS) (*)[D]

Wetland microbial communities from Twitchell Islan ... tland Surface Sediment Feb2011 Site A1 Tule, Oct 2011 assem) (MER-FS) (*)[D]

Wetland microbial communities from Twitchell Islan ... nd Surface Sediment Feb2011 Site A2 Cattail Sept 2011 assem) (MER-FS) (*)[D]

Wetland microbial communities from Twitchell Islan ... nd Surface Sediment Feb2011 Site A2 Cattail Sept 2011 assem) (MER-FS) (*)[D]

Wetland microbial communities from Twitchell Islan ... Sediment Feb2011 Site A2 Cattail, Assem Ctgs Jan 2012 Assem) (MER-FS) (*)[D]

Wetland microbial communities from Twitchell Islan ... Sediment Feb2011 Site A2 Cattail, Ctgs/UnRds Jan 2012 Assem) (MER-FS) (*)[D]

Wetland microbial communities from Twitchell Islan ... iment Feb2011 Site A2 Cattail, Ctgs/UnReads Sept 2011 assem) (MER-FS) (*)[D]

Wetland microbial communities from Twitchell Islan ... nd Surface Sediment Feb2011 Site A2 Cattail, Oct 2011 assem) (MER-FS) (*)[D]

Wetland microbial communities from Twitchell Islan ... ulk (Wetland Surface Sediment Feb2011 Site B1 Bulk Feb 2012) (MER-FS) (*)[D]

Wetland microbial communities from Twitchell Islan ... tland Surface Sediment Feb2011 Site B1 Bulk, Oct 2011 assem) (MER-FS) (*)[D]

Wetland microbial communities from Twitchell Islan ... tland Surface Sediment Feb2011 Site B1 Bulk, Oct 2011 assem) (MER-FS) (*)[D]

Wetland microbial communities from Twitchell Islan ... tland Surface Sediment Feb2011 Site B1 Bulk, Oct 2011 assem) (MER-FS) (*)[D]

Wetland microbial communities from Twitchell Islan ... tland Surface Sediment Feb2011 Site B1 Bulk, Oct 2011 assem) (MER-FS) (*)[D]

Wetland microbial communities from Twitchell Islan ... tland Surface Sediment Feb2011 Site B1 Bulk, Oct 2011 assem) (MER-FS) (*)[D]

Wetland microbial communities from Twitchell Islan ... tland Surface Sediment Feb2011 Site B1 Bulk, Oct 2011 assem) (MER-FS) (*)[D]

Wetland microbial communities from Twitchell Islan ... Sediment Feb2011 Site B1 Cattail, Assem Ctgs Sep 2011 assem) (MER-FS) (*)[D]

Wetland microbial communities from Twitchell Islan ... nd Surface Sediment Feb2011 Site B1 Cattail, Sep 2011 assem) (MER-FS) (*)[D]

Wetland microbial communities from Twitchell Islan ... ce Sediment Feb2011 Site B2 Bulk, Assem Ctgs Oct 2011 assem) (MER-FS) (*)[D]

Wetland microbial communities from Twitchell Islan ... tland Surface Sediment Feb2011 Site B2 Bulk, Oct 2011 assem) (MER-FS) (*)[D]

Wetland microbial communities from Twitchell Islan ... etland Surface Sediment Feb2011 Site B2 Tule Oct 2011 assem) (MER-FS) (*)[D]

Wetland microbial communities from Twitchell Islan ... Sediment Feb2011 Site B2 Tule, Ctgs/UnReads Oct 2011 assem) (MER-FS) (*)[D]

Wetland microbial communities from Twitchell Islan ... etland Surface Sediment Feb2011 Site L1 Bulk Jan 2012 assem) (MER-FS) (*)[D]

Wetland microbial communities from Twitchell Islan ... Sediment Feb2011 Site L1 Bulk, Ctgs/UnReads Jan 2012 assem) (MER-FS) (*)[D]

Wetland microbial communities from Twitchell Islan ... Sediment Feb2011 Site L1 Cattail, Assem Ctgs Sep 2011 assem) (MER-FS) (*)[D]

Wetland microbial communities from Twitchell Islan ... Sediment Feb2011 Site L1 Cattail, Assem Ctgs Sep 2011 assem) (MER-FS) (*)[D]

Wetland microbial communities from Twitchell Islan ... nd Surface Sediment Feb2011 Site L1 Cattail, Sep 2011 assem) (MER-FS) (*)[D]

Wetland microbial communities from Twitchell Islan ... nd Surface Sediment Feb2011 Site L1 Cattail, Sep 2011 assem) (MER-FS) (*)[D]

Wetland microbial communities from Twitchell Island in the S ... ent Feb2011 Site L1 Cattail,51 Arch Meth Ctg Sep 2011 assem) (*)[D]

Wetland microbial communities from Twitchell Islan ... ce Sediment Feb2011 Site L2 Tule, Assem Ctgs Sep 2011 assem) (MER-FS) (*)[D]

Wetland microbial communities from Twitchell Islan ... tland Surface Sediment Feb2011 Site L2 Tule, Sep 2011 assem) (MER-FS) (*)[D]

Marine microbial communities from six Antarctic regions

DNA Fragments from Six Antarctic Marine environments (*)[D]

Deep ocean microbial communities from the Global Malaspina Expedition

Deep seawater metagenome MP2159 (Deep seawater metagenome MP2159, ASSEMBLY_DATE=20130220) (MER-FS) (*)[D]

Marine microbial communities from the Eastern Subtropical North Pacific Ocean, Expanding Oxygen minimum zones

Line P August 2008 P26 500m (Line P August 2008 P26 500m, March 2012 Assem) (MER-FS) (*)[D]

Marine microbial communities from Deepwater Horizon Oil Spill

Marine microbial communities from Deepwater Horizon Oil Spill, sample DHOS BM58 plume (MER-FS) (*)[D]

Marine microbial communities from Deepwater Horizon Oil Spill, sample DHOS BM58 plume (BM58 Illumina assembly) (MER-FS) (*)[D]

Marine microbial communities from Deepwater Horizon Oil Spill, sample DHOS OV011 (MER-FS) (*)[D]

Fossil microbial community from Whale Fall, Santa Cruz Basin of the Pacific Ocean

Fossil microbial community from Whale Fall at Santa Cruz Basin of the Pacific Ocean Sample #1 (MER-FS) (*)[D]

Fossil microbial community from Whale Fall at Santa Cruz Basin of the Pacific Ocean Sample #2 (MER-FS) (*)[D]

Fossil microbial community from Whale Fall at Santa Cruz Basin of the Pacific Ocean Sample #3 (MER-FS) (*)[D]

Hot (42-90C)

Hot spring microbial communities from Yellowstone Bison Hot Spring Pool

1_050719N (MER-FS) (*)[D]

2_050719S (MER-FS) (*)[D]

3_050719R (MER-FS) (*)[D]

4_050719Q (MER-FS) (*)[D]

5_050719P (MER-FS) (*)[D]

Hypersaline water microbial communities from Lyngbya mats, Guerrero Negro, Mexico and Elkhorn Slough mats, California, USA

Beowulf Spring Fe mat T=65-68 Metatranscriptome (Beowulf Spring Fe mat T=65-68, June 2012 Assem) (MER-FS) (*)[D]

Cistern Spring metagenome (Cistern Spring - All contigs, June 2012 Assem) (MER-FS) (*)[D]

Cistern Spring metagenome (Cistern Spring, June 2012 Assem) (MER-FS) (*)[D]

Conch Spring, C T=80-84 metagenome (Conch Spring, C T=80-84, July 2012 Assem) (MER-FS) (*)[D]

Echinus Geyser, transect B T=78-80 C metagenome (Echinus Geyser transect B T=78-80 C, June 2012 Assem) (MER-FS) (*)[D]

Grendel Spring T=76-80 metatranscriptome (Grendel Spring T=76-80, June 2012 Assem) (MER-FS) (*)[D]

Mammoth Hot Spring (Liberty Cap Streamers) T=72 me ... th Hot Spring (Liberty Cap Streamers) T=72, June 2012 Assem) (MER-FS) (*)[D]

Octopus Spring Streamers T=80-84 metagenome (Octopus Spring Streamers T=80-84, June 2012 Assem) (MER-FS) (*)[D]

One Hundred Spring Plain, T=66-70 metagenome (One Hundred Spring Plain T=66-70, June 2012 Assem) (MER-FS) (*)[D]

One Hundred Spring Plain, T=74-76 metagenome (One Hundred Spring Plain T=74-76, June 2012 Assem) (MER-FS) (*)[D]

Hot Spring microbial communities from Yellowstone Obsidian Hot Spring

Hot Spring microbial communities from Yellowstone Obsidian Hot Spring, Sample 10594 (*)[D]

Hot spring microbial communities from Yellowstone National Park, US

Hot spring microbial community from Beowulf Spring ... nal Park, sample YNP_Beowulf Spring_D (YNP_Beowulf Spring_D) (MER-FS) (*)[D]

Hot spring microbial community from Beowulf Spring ... nal Park, sample YNP_Beowulf Spring_E (YNP_Beowulf Spring_E) (MER-FS) (*)[D]

Hot spring microbial community from Yellowstone Hot Springs, sample YNP8 from OSP Spring (MER-FS) (*)[D]

Hot spring microbial community from Yellowstone Hot Springs, sample YNP8 from OSP Spring (MER-FS) (*)[D]

Hot spring microbial community from Yellowstone Hot Springs, sample YNP1 from Alice Springs, Crater Hills (MER-FS) (*)[D]

Hot spring microbial community from Yellowstone Hot Springs, sample YNP1 from Alice Springs, Crater Hills (MER-FS) (*)[D]

Hot spring microbial community from Yellowstone Hot Springs, sample YNP10 from Narrow Gauge (*)[D]

Hot spring microbial community from Yellowstone Hot Springs, sample YNP10 from Narrow Gauge (*)[D]

Hot spring microbial community from Yellowstone Hot Springs, sample YNP11 from Octopus Springs (MER-FS) (*)[D]

Hot spring microbial community from Yellowstone Hot Springs, sample YNP11 from Octopus Springs (MER-FS) (*)[D]

Hot spring microbial community from Yellowstone Ho ... rings, sample YNP12 from Calcite Springs, Tower Falls Region (MER-FS) (*)[D]

Hot spring microbial community from Yellowstone Ho ... rings, sample YNP12 from Calcite Springs, Tower Falls Region (MER-FS) (*)[D]

Hot spring microbial community from Yellowstone Hot Springs, sample YNP13 from Bechler Spring (MER-FS) (*)[D]

Hot spring microbial community from Yellowstone Hot Springs, sample YNP13 from Bechler Spring (MER-FS) (*)[D]

Hot spring microbial community from Yellowstone Hot Springs, sample YNP14 from OSP Spring (MER-FS) (*)[D]

Hot spring microbial community from Yellowstone Hot Springs, sample YNP14 from OSP Spring (MER-FS) (*)[D]

Hot spring microbial community from Yellowstone Hot Springs, sample YNP15 from Mushroom Spring (MER-FS) (*)[D]

Hot spring microbial community from Yellowstone Hot Springs, sample YNP15 from Mushroom Spring (MER-FS) (*)[D]

Hot spring microbial community from Yellowstone Hot Springs, sample YNP16 from Fairy Spring Red Layer (MER-FS) (*)[D]

Hot spring microbial community from Yellowstone Hot Springs, sample YNP16 from Fairy Spring Red Layer (MER-FS) (*)[D]

Hot spring microbial community from Yellowstone Hot Springs, sample YNP17 from Obsidian Pool Prime (MER-FS) (*)[D]

Hot spring microbial community from Yellowstone Hot Springs, sample YNP17 from Obsidian Pool Prime (MER-FS) (*)[D]

Hot spring microbial community from Yellowstone Hot Springs, sample YNP18 from Washburn Springs #1 (MER-FS) (*)[D]

Hot spring microbial community from Yellowstone Hot Springs, sample YNP18 from Washburn Springs #1 (MER-FS) (*)[D]

Hot spring microbial community from Yellowstone Hot Springs, sample YNP19 from Cistern Spring (MER-FS) (*)[D]

Hot spring microbial community from Yellowstone Hot Springs, sample YNP19 from Cistern Spring (MER-FS) (*)[D]

Hot spring microbial community from Yellowstone Hot Springs, sample YNP2 from Nymph Lake 10 (MER-FS) (*)[D]

Hot spring microbial community from Yellowstone Hot Springs, sample YNP2 from Nymph Lake 10 (*)[D]

Hot spring microbial community from Yellowstone Hot Springs, sample YNP2 from Nymph Lake 10 (*)[D]

Hot spring microbial community from Yellowstone Ho ... sample YNP20 from Bath Lake Vista Annex - Purple-Sulfur Mats (MER-FS) (*)[D]

Hot spring microbial community from Yellowstone Ho ... sample YNP20 from Bath Lake Vista Annex - Purple-Sulfur Mats (MER-FS) (*)[D]

Hot spring microbial community from Yellowstone Ho ... prings, sample YNP3 from Monarch Geyser, Norris Geyser Basin (MER-FS) (*)[D]

Hot spring microbial community from Yellowstone Ho ... prings, sample YNP3 from Monarch Geyser, Norris Geyser Basin (MER-FS) (*)[D]

Hot spring microbial community from Yellowstone Ho ... prings, sample YNP3 from Monarch Geyser, Norris Geyser Basin (MER-FS) (*)[D]

Hot spring microbial community from Yellowstone Hot Springs, sample YNP4 from Joseph's Coat Springs (MER-FS) (*)[D]

Hot spring microbial community from Yellowstone Hot Springs, sample YNP4 from Joseph's Coat Springs (MER-FS) (*)[D]

Hot spring microbial community from Yellowstone Hot Springs, sample YNP5 from Bath Lake Vista Annex (MER-FS) (*)[D]

Hot spring microbial community from Yellowstone Hot Springs, sample YNP5 from Bath Lake Vista Annex (MER-FS) (*)[D]

Hot spring microbial community from Yellowstone Hot Springs, sample YNP6 from White Creek Site 3 (MER-FS) (*)[D]

Hot spring microbial community from Yellowstone Hot Springs, sample YNP6 from White Creek Site 3 (MER-FS) (*)[D]

Hot spring microbial community from Yellowstone Hot Springs, sample YNP7 from Chocolate Pots (MER-FS) (*)[D]

Hot spring microbial community from Yellowstone Hot Springs, sample YNP7 from Chocolate Pots (MER-FS) (*)[D]

Hot spring microbial community from Yellowstone Ho ... Springs, sample YNP9 from Dragon Spring, Norris Geyser Basin (MER-FS) (*)[D]

Hot spring microbial community from Yellowstone Ho ... Springs, sample YNP9 from Dragon Spring, Norris Geyser Basin (MER-FS) (*)[D]

Sediment and Water microbial communities from Great Boiling Spring

Water microbial communities from Great Boiling Spring, Nevada, sample 1 (13 Aug 2010 assembly with PE data) (MER-FS) (*)[D]

Water microbial communities from Great Boiling Spring, Nevada, sample 1 (Water borne 27 Oct 2010 assembly) (MER-FS) (*)[D]

Water microbial communities from Great Boiling Spring, Nevada, sample 1 (Water borne community) (MER-FS) (*)[D]

Water microbial communities from Great Boiling Spr ... lytic enrichment S 77C (Cellulolytic enrichment S 77C water) (MER-FS) (*)[D]

Water microbial communities from Great Boiling Spr ... 7C (GBS Cellulolytic enrichment S 77C water, Feb 2012 assem) (MER-FS) (*)[D]

Acidic

Archaeal viriome from Yellowstone Hot Springs

Crater Hills (*)[D]

Nymph Lake Bulk Water (*)[D]

Sediment and Water microbial communities from Great Boiling Spring

Sediment microbial communities from Great Boiling ... ichment CS 85C (GBS Cellulolytic enrichment CS 85C sediment) (MER-FS) (*)[D]

Sediment microbial communities from Great Boiling ... olytic enrichment CS 85C sediment, Combined June 2011 assem) (MER-FS) (*)[D]

Sediment microbial communities from Great Boiling ... GBS Cellulolytic enrichment CS 85C sediment, Feb 2012 assem) (MER-FS) (*)[D]

Sediment microbial communities from Great Boiling ... chment Sediment 77C (Cellulolytic enrichment S 77C sediment) (MER-FS) (*)[D]

Sediment microbial communities from Great Boiling ... C (GBS Cellulolytic enrichment 77S sediment, Feb 2012 assem) (MER-FS) (*)[D]

Sediment microbial communities from Great Boiling ... enrichment CS 77C (Cellulolytic enrichment CS 77C sediment) (MER-FS) (*)[D]

Sediment microbial communities from Great Boiling ... olytic enrichment CS 77C sediment, Combined June 2011 assem) (MER-FS) (*)[D]

Sediment microbial communities from Great Boiling ... GBS Cellulolytic enrichment CS 77C sediment, Feb 2012 assem) (MER-FS) (*)[D]

Sediment microbial communities from Great Boiling ... ing, Nevada, sample from surface sediment (Surface sediment) (MER-FS) (*)[D]

Hot spring microbial communities from Yellowstone National Park, US

Hot spring microbial communities from Yellowstone ... tional Park, One Hundred Springs Plain, sample OSP_B (OSP_B) (MER-FS) (*)[D]

Hot spring microbial communities from Yellowstone ... tional Park, One Hundred Springs Plain, sample OSP_C (OSP_C) (MER-FS) (*)[D]

Hot spring microbial communities from Yellowstone ... tional Park, One Hundred Springs Plain, sample OSP_D (OSP_D) (MER-FS) (*)[D]

Sediment and Water microbial communities from Great Boiling Spring

Water viral communities from Great Boiling Spring, Nevada (Water borne viral community) (MER-FS) (*)[D]

Acidic water microbial communities from Richmond acid mine drainage

Acid Mine Drainage (*)[D]

Acidic water microbial communities from Richmond acid mine drainage

Acid Mine Drainage (ARMAN) microbial communities f ... ichmond mine, Iron Mountain, CA, sample from Ultra Back A BS (MER-FS) (*)[D]

Groundwater microbial communities from subsurface biofilms in sulfidic aquifier in Frasassi Gorge, Italy

Groundwater microbial communities from subsurface ... iofilm samples from two redox zones-FS06_10, Dec 2011 Assem) (MER-FS) (*)[D]

Groundwater microbial communities from subsurface ... Biofilm samples from two redox zones-FS08_3, Oct 2011 Assem) (MER-FS) (*)[D]

Groundwater microbial communities from subsurface ... Biofilm samples from two redox zones-GS09_5, Oct 2011 Assem) (MER-FS) (*)[D]

Groundwater microbial communities from subsurface ... iofilm samples from two redox zones-GS10_10, Oct 2011 Assem) (MER-FS) (*)[D]

Groundwater microbial communities from subsurface ... Biofilm samples from two redox zones-PC08_3, Oct 2011 Assem) (MER-FS) (*)[D]

Groundwater microbial communities from subsurface ... iofilm samples from two redox zones-PC08_64, Feb 2012 Assem) (MER-FS) (*)[D]

Groundwater microbial communities from subsurface ... iofilm samples from two redox zones-PC08_66, Dec 2011 Assem) (MER-FS) (*)[D]

Rifle Groundwater A1 ((Wrighton et al. 2012 Metagenome MDM reference)) (MER-FS) (*)[P]

Subsurface groundwater monitoring well GMW60B unco ... r monitoring well GMW60B uncontam upgr,5.4m, Oct 2012 Assem) (MER-FS) (*)[D]

Lake Huron sinkhole microbial mat community

Sinkhole freshwater microbial communities from Lake Huron, US, Ph40x (*)[D]

Sinkhole freshwater microbial communities from Lake Huron, US, Sample 419 (MER-FS) (*)[D]

Groundwater microbial communities from subsurface biofilms in sulfidic aquifier in Frasassi Gorge, Italy

Groundwater microbial communities from subsurface ... Biofilm samples from two redox zones-AS07_7, Dec 2011 Assem) (MER-FS) (*)[D]

Groundwater microbial communities from subsurface ... Biofilm samples from two redox zones-LI09_3, Jan 2012 Assem) (MER-FS) (*)[D]

Groundwater microbial communities from subsurface ... Biofilm samples from two redox zones-LI09_4, Oct 2011 Assem) (MER-FS) (*)[D]

naphthalene biodegradation metagenome

Naphthalene biodegradation metagenome 12C5dose (Me ... biodegradation metagenome 12C5dose, ASSEMBLY_DATE=20130123) (MER-FS) (*)[D]

Naphthalene biodegradation metagenome 12C8dose (Me ... biodegradation metagenome 12C8dose, ASSEMBLY_DATE=20130227) (MER-FS) (*)[D]

Naphthalene biodegradation metagenome 13C3doseA (M ... biodegradation metagenome 13C3doseA, ASSEMBLY_DATE=20130123) (MER-FS) (*)[D]

Naphthalene biodegradation metagenome 13C3doseB (M ... biodegradation metagenome 13C3doseB, ASSEMBLY_DATE=20130123) (MER-FS) (*)[D]

Naphthalene biodegradation metagenome 13C3doseC (M ... biodegradation metagenome 13C3doseC, ASSEMBLY_DATE=20130123) (MER-FS) (*)[D]

Naphthalene biodegradation metagenome 13C5doseA (M ... biodegradation metagenome 13C5doseA, ASSEMBLY_DATE=20130123) (MER-FS) (*)[D]

Naphthalene biodegradation metagenome 13C5doseB (M ... biodegradation metagenome 13C5doseB, ASSEMBLY_DATE=20130123) (MER-FS) (*)[D]

Naphthalene biodegradation metagenome 13C5doseC (M ... biodegradation metagenome 13C5doseC, ASSEMBLY_DATE=20130123) (MER-FS) (*)[D]

Naphthalene biodegradation metagenome 13C8doseA (M ... biodegradation metagenome 13C8doseA, ASSEMBLY_DATE=20130409) (MER-FS) (*)[D]

Naphthalene biodegradation metagenome 13C8doseB (M ... biodegradation metagenome 13C8doseB, ASSEMBLY_DATE=20130123) (MER-FS) (*)[D]

Naphthalene biodegradation metagenome 13C8doseC (M ... biodegradation metagenome 13C8doseC, ASSEMBLY_DATE=20130123) (MER-FS) (*)[D]

Ground water

Oak Ridge Pristine Groundwater FRC FW301 (MER-FS) (*)[D]

Uranium Contaminated Groundwater FW106 (MER-FS) (*)[D]

Subsurface groundwater monitoring well GMW36 conta ... monitoring well GMW36 contaminated, ASSEMBLY_DATE=20130123) (MER-FS) (*)[D]

Subsurface groundwater monitoring well GMW37 conta ... ring well GMW37 contaminated, 5.8 m, ASSEMBLY_DATE=20130411) (MER-FS) (*)[D]

Subsurface groundwater monitoring well GMW46 conta ... er monitoring well GMW46 contaminated, 5.4m, Oct 2012 Assem) (MER-FS) (*)[D]

Freshwater microbial communities from Lake Kinneret

Aquatic microbial communities from Lake Kinneret (01) (*)[D]

Aquatic microbial communities from Lake Kinneret (02) (*)[D]

Aquatic microbial communities from Lake Kinneret (03) (*)[D]

Aquatic microbial communities from Lake Kinneret (04) (*)[D]

Aquatic microbial communities from Lake Kinneret (05) (*)[D]

Aquatic microbial communities from Lake Kinneret (06) (*)[D]

Aquatic microbial communities from Lake Kinneret (07) (*)[D]

Aquatic microbial communities from Lake Kinneret (08) (*)[D]

Fresh water microbial communities from LaBonte Lake

Fresh water microbial communities from LaBonte Lak ... bloom material peak-bloom 1 (algal/cyano bloom peak-bloom 1) (MER-FS) (*)[D]

Fresh water microbial communities from LaBonte Lak ... bloom material peak-bloom 2 (algal/cyano bloom peak-bloom 2) (MER-FS) (*)[D]

Fresh water microbial communities from LaBonte Lak ... Wyoming, sample from peak-bloom 2 (Peak bloom metagenome 2) (MER-FS) (*)[D]

Fresh water microbial communities from LaBonte Lake, Laramie, Wyoming, sample from post-bloom (post-bloom) (MER-FS) (*)[D]

Fresh water microbial communities from LaBonte Lake, Laramie, Wyoming, sample from pre-bloom (pre-bloom) (MER-FS) (*)[D]

Freshwater microbial communities from Antarctic Deep Lake

Freshwater microbial communities from Antarctic Deep Lake, sample 13m 0.1um (13m 0.1um 454 only) (MER-FS) (*)[D]

Freshwater microbial communities from Antarctic Deep Lake, sample 24m 0.1um (24 m 0.1 um 454 only) (MER-FS) (*)[D]

Freshwater microbial communities from Antarctic Deep Lake, sample 24m 0.8um (24 m 0.8 um 454 only Nov2010) (MER-FS) (*)[D]

Freshwater microbial communities from Antarctic De ... ample 24m 0.8um (24 m 0.8 um 454/Illumina combined Jan 2011) (MER-FS) (*)[D]

Freshwater microbial communities from Antarctic Deep Lake, sample 24m 0.8um (24 m 0.8 um Illumina only) (MER-FS) (*)[D]

Freshwater microbial communities from Antarctic Deep Lake, sample 24m 3.0um (24 m 3.0 um Illumina only) (MER-FS) (*)[D]

Freshwater microbial communities from Antarctic Deep Lake, sample 24m 3.0um (24 m 3.0 um Sept 2010 combined) (MER-FS) (*)[D]

Freshwater microbial communities from Antarctic Deep Lake, sample 24m 3.0um (24 m 3.0 um) (MER-FS) (*)[D]

Freshwater microbial communities from Antarctic De ... le 36m 3.0um, 0.8um, 0.1um pool (36m 3, 0.8 and 0.1 um pool) (MER-FS) (*)[D]

Freshwater microbial communities from Antarctic De ... ke, sample 36m 3.0um, 0.8um, 0.1um pool (HWGG+HTSY Jan 2011) (MER-FS) (*)[D]

Freshwater microbial communities from Antarctic Deep Lake, sample 5mRS 0.1um (5 mRS 0.1um 454 only) (MER-FS) (*)[D]

Freshwater microbial communities from Lake Sakinaw in Canada

Sakinaw Lake 454 metagenomics (120m): eDNA_2 (Sakinaw Lake metagenomic (120m), Feb 2012 assem) (*)[D]

Sediment microbial communities from Lake Washington for Methane and Nitrogen Cycles

Sediment microbial communities from Lake Washingto ... anaerobic plus nitrate (Flow sorted anaerobic plus nitrate) (MER-FS) (*)[D]

Freshwater microbial communities from Trout Bog Lake, WI and Lake Mendota, IL

Freshwater microbial communities from Lake Mendota ... (Lake Mendota Practice 15JUN2010 epilimnion June 2011 assem) (MER-FS) (*)[D]

Freshwater microbial communities from Lake Mendota ... ice 15JUN2010 epilimnion,new10pct subsampleHZIB MergedReads) (MER-FS) (*)[D]

Freshwater microbial communities from Lake Mendota ... m Practice 15JUN2010 epilimnion (NA, ASSEMBLY_DATE=20130319) (MER-FS) (*)[D]

Freshwater microbial communities from Lake Mendota ... (Lake Mendota Practice 20APR2010 epilimnion June 2011 assem) (MER-FS) (*)[D]

Freshwater microbial communities from Lake Mendota ... ce 20APR2010 epilimnion,new10pct subsampleHZHY Merged Reads) (MER-FS) (*)[D]

Freshwater microbial communities from Lake Mendota ... m Practice 20APR2010 epilimnion (NA, ASSEMBLY_DATE=20130319) (MER-FS) (*)[D]

Freshwater microbial communities from Lake Mendota ... m Practice 20APR2010 epilimnion (NA, ASSEMBLY_DATE=20130410) (MER-FS) (*)[D]

Freshwater microbial communities from Lake Mendota ... (Lake Mendota Practice 29OCT2010 epilimnion June 2011 assem) (MER-FS) (*)[D]

Freshwater microbial communities from Lake Mendota ... ice 29OCT2010 epilimnion,new10pct subsampleHZIA MergedReads) (MER-FS) (*)[D]

Freshwater microbial communities from Lake Mendota ... m Practice 29OCT2010 epilimnion (NA, ASSEMBLY_DATE=20130319) (MER-FS) (*)[D]

Freshwater microbial communities from Trout Bog La ... n (Trout Bog Practice 03JUN2009 epilimnion June 2011 assem) (MER-FS) (*)[D]

Freshwater microbial communities from Trout Bog La ... n (Trout Bog Practice 03JUN2009 epilimnion June 2011 assem) (MER-FS) (*)[D]

Freshwater microbial communities from Trout Bog La ... Bog Practice 03JUN2009 epilimnion 1000 subsample, June 2011) (MER-FS) (*)[D]

Freshwater microbial communities from Trout Bog La ... ice 03JUN2009 epilimnion,new10pct subsampleHZIF MergedReads) (MER-FS) (*)[D]

Freshwater microbial communities from Trout Bog La ... ce 18AUG2009 epilimnion,new10pct subsampleHZHX Merged Reads) (MER-FS) (*)[D]

Freshwater microbial communities from Trout Bog La ... on (Trout Bog Practice 18AUG2009 epilimnion June 2011 assem) (MER-FS) (*)[D]

Freshwater microbial communities from Trout Bog La ... on (Trout Bog Practice 18AUG2009 epilimnion June 2011 assem) (MER-FS) (*)[D]

Trout Bog Lake June 7 2007 Epilimnion (Trout Bog Lake Combined Assembly 47 Epilimnion Samples, Aug 2012 Assem) (MER-FS) (*)[D]

Microbial Interactions in Extremophilic Mat Communities

Hot Lake mat section #1 (Hot Lake mat section #1, ASSEMBLY_DATE=20130221) (MER-FS) (*)[D]

Hot Lake mat section #2 (Hot Lake mat section #2, ASSEMBLY_DATE=20130213) (MER-FS) (*)[D]

Hot Lake mat section #3 (Hot Lake mat section #3, ASSEMBLY_DATE=20130221) (MER-FS) (*)[D]

Hot Lake mat section #4 (Hot Lake mat section #4, ASSEMBLY_DATE=20130115) (MER-FS) (*)[D]

Hot Lake mat section #5 (Hot Lake mat section #5, ASSEMBLY_DATE=20130115) (MER-FS) (*)[D]

Metagenomics and metatranscriptomics of the Lake Erie 'dead zone': a seasonal source of greenhouse gases

Lake Erie CCB epilimnion July 2011 (Lake Erie CCB epilimnion July 2011, ASSEMBLY_DATE=20121208) (MER-FS) (*)[D]

High-resolution temporal and spatial dynamics of microbially-mediated carbon processing revealed though time-series metagenomics in freshwater lakes

Lake Mendota Deep Hole Epilimnion 05Aug08 (Lake Me ... Hole Epilimnion 05Aug08, 10pct subsample CXSH Merged Reads) (MER-FS) (*)[D]

Lake Mendota Deep Hole Epilimnion 05Aug08 (Lake Mendota Deep Hole Epilimnion 05Aug08, ASSEMBLY_DATE=20130319) (MER-FS) (*)[D]

Lake Mendota Deep Hole Epilimnion 05May10 (Lake Me ... Hole Epilimnion 05May10, 10pct subsample CXOT Merged Reads) (MER-FS) (*)[D]

Lake Mendota Deep Hole Epilimnion 05May10 (Lake Mendota Deep Hole Epilimnion 05May10, ASSEMBLY_DATE=20130319) (MER-FS) (*)[D]

Lake Mendota Deep Hole Epilimnion 08Oct08 (Lake Me ... Hole Epilimnion 08Oct08, 10pct subsample CSGY Merged Reads) (MER-FS) (*)[D]

Lake Mendota Deep Hole Epilimnion 08Oct08 (Lake Mendota Deep Hole Epilimnion 08Oct08, ASSEMBLY_DATE=20130319) (MER-FS) (*)[D]

Lake Mendota Deep Hole Epilimnion 09Jul08 (Lake Me ... Hole Epilimnion 09Jul08, 10pct subsample CXSP Merged Reads) (MER-FS) (*)[D]

Lake Mendota Deep Hole Epilimnion 09Jul08 (Lake Mendota Deep Hole Epilimnion 09Jul08, ASSEMBLY_DATE=20130319) (MER-FS) (*)[D]

Lake Mendota Deep Hole Epilimnion 12Sep08 (Lake Me ... Hole Epilimnion 12Sep08, 10pct subsample CSGW Merged Reads) (MER-FS) (*)[D]

Lake Mendota Deep Hole Epilimnion 12Sep08 (Lake Mendota Deep Hole Epilimnion 12Sep08, ASSEMBLY_DATE=20130319) (MER-FS) (*)[D]

Lake Mendota Deep Hole Epilimnion 13Aug08 (Lake Me ... Hole Epilimnion 13Aug08, 10pct subsample CSGT Merged Reads) (MER-FS) (*)[D]

Lake Mendota Deep Hole Epilimnion 13Aug08 (Lake Mendota Deep Hole Epilimnion 13Aug08, ASSEMBLY_DATE=20130319) (MER-FS) (*)[D]

Lake Mendota Deep Hole Epilimnion 13Jun10 (Lake Me ... Hole Epilimnion 13Jun10, 10pct subsample CXSG Merged Reads) (MER-FS) (*)[D]

Lake Mendota Deep Hole Epilimnion 13Jun10 (Lake Mendota Deep Hole Epilimnion 13Jun10, ASSEMBLY_DATE=20130319) (MER-FS) (*)[D]

Lake Mendota Deep Hole Epilimnion 13Sep08 (Lake Me ... Hole Epilimnion 13Sep08, 10pct subsample CXPY Merged Reads) (MER-FS) (*)[D]

Lake Mendota Deep Hole Epilimnion 13Sep08 (Lake Mendota Deep Hole Epilimnion 13Sep08, ASSEMBLY_DATE=20130319) (MER-FS) (*)[D]

Lake Mendota Deep Hole Epilimnion 16Jul10 (Lake Me ... Hole Epilimnion 16Jul10, 10pct subsample CSNS Merged Reads) (MER-FS) (*)[D]

Lake Mendota Deep Hole Epilimnion 16Jul10 (Lake Mendota Deep Hole Epilimnion 16Jul10, ASSEMBLY_DATE=20130319) (MER-FS) (*)[D]

Lake Mendota Deep Hole Epilimnion 17Oct08 (Lake Me ... Hole Epilimnion 17Oct08, 10pct subsample CXSN Merged Reads) (MER-FS) (*)[D]

Lake Mendota Deep Hole Epilimnion 17Oct08 (Lake Mendota Deep Hole Epilimnion 17Oct08, ASSEMBLY_DATE=20130319) (MER-FS) (*)[D]

Lake Mendota Deep Hole Epilimnion 18Jul08 (Lake Me ... Hole Epilimnion 18Jul08, 10pct subsample CSCX Merged Reads) (MER-FS) (*)[D]

Lake Mendota Deep Hole Epilimnion 18Jul08 (Lake Mendota Deep Hole Epilimnion 18Jul08, ASSEMBLY_DATE=20130319) (MER-FS) (*)[D]

Lake Mendota Deep Hole Epilimnion 19Jul08 (Lake Me ... Hole Epilimnion 19Jul08, 10pct subsample CSNB Merged Reads) (MER-FS) (*)[D]

Lake Mendota Deep Hole Epilimnion 19Jul08 (Lake Mendota Deep Hole Epilimnion 19Jul08, ASSEMBLY_DATE=20130319) (MER-FS) (*)[D]

Lake Mendota Deep Hole Epilimnion 20Aug08 (Lake Me ... Hole Epilimnion 20Aug08, 10pct subsample CXON Merged Reads) (MER-FS) (*)[D]

Lake Mendota Deep Hole Epilimnion 20Aug08 (Lake Mendota Deep Hole Epilimnion 20Aug08, ASSEMBLY_DATE=20130319) (MER-FS) (*)[D]

Lake Mendota Deep Hole Epilimnion 20May10 (Lake Me ... Hole Epilimnion 20May10, 10pct subsample CXPU Merged Reads) (MER-FS) (*)[D]

Lake Mendota Deep Hole Epilimnion 20May10 (Lake Mendota Deep Hole Epilimnion 20May10, ASSEMBLY_DATE=20130319) (MER-FS) (*)[D]

Lake Mendota Deep Hole Epilimnion 21Jul08 (Lake Me ... Hole Epilimnion 21Jul08, 10pct subsample CXPP Merged Reads) (MER-FS) (*)[D]

Lake Mendota Deep Hole Epilimnion 21Jul08 (Lake Mendota Deep Hole Epilimnion 21Jul08, ASSEMBLY_DATE=20130319) (MER-FS) (*)[D]

Lake Mendota Deep Hole Epilimnion 23Jul08 (Lake Me ... Hole Epilimnion 23Jul08, 10pct subsample CSGH Merged Reads) (MER-FS) (*)[D]

Lake Mendota Deep Hole Epilimnion 23Jul08 (Lake Mendota Deep Hole Epilimnion 23Jul08, ASSEMBLY_DATE=20130319) (MER-FS) (*)[D]

Lake Mendota Deep Hole Epilimnion 23Jul08 (Lake Mendota Deep Hole Epilimnion 23Jul08, ASSEMBLY_DATE=20130412) (MER-FS) (*)[D]

Lake Mendota Deep Hole Epilimnion 25Sep08 (Lake Me ... Hole Epilimnion 25Sep08, 10pct subsample CSCW Merged Reads) (MER-FS) (*)[D]

Lake Mendota Deep Hole Epilimnion 25Sep08 (Lake Mendota Deep Hole Epilimnion 25Sep08, ASSEMBLY_DATE=20130319) (MER-FS) (*)[D]

Lake Mendota Deep Hole Epilimnion 26Jun08 (Lake Me ... Hole Epilimnion 26Jun08, 10pct subsample CSNA Merged Reads) (MER-FS) (*)[D]

Lake Mendota Deep Hole Epilimnion 26Jun08 (Lake Mendota Deep Hole Epilimnion 26Jun08, ASSEMBLY_DATE=20130319) (MER-FS) (*)[D]

Lake Mendota Deep Hole Epilimnion 27Aug08 (Lake Me ... Hole Epilimnion 27Aug08, 10pct subsample CSHY Merged Reads) (MER-FS) (*)[D]

Lake Mendota Deep Hole Epilimnion 27Aug08 (Lake Mendota Deep Hole Epilimnion 27Aug08, ASSEMBLY_DATE=20130319) (MER-FS) (*)[D]

Lake Mendota Deep Hole Epilimnion 30Jul08 (Lake Me ... Hole Epilimnion 30Jul08, 10pct subsample CXSC Merged Reads) (MER-FS) (*)[D]

Lake Mendota Deep Hole Epilimnion 30Jul08 (Lake Mendota Deep Hole Epilimnion 30Jul08, ASSEMBLY_DATE=20130319) (MER-FS) (*)[D]

Lake Mendota Deep Hole Epilimnion 31Aug10 (Lake Me ... Hole Epilimnion 31Aug10, 10pct subsample CSGO Merged Reads) (MER-FS) (*)[D]

Lake Mendota Deep Hole Epilimnion 31Aug10 (Lake Mendota Deep Hole Epilimnion 31Aug10, ASSEMBLY_DATE=20130319) (MER-FS) (*)[D]

Freshwater microbial communities from Trout Bog Lake, WI and Lake Mendota, WI

Trout Bog Lake April 30 2008 Epilimnion (Trout Bog ... il 30 2008 Epilimnion, new10pct subsample IHTP Merged Reads) (MER-FS) (*)[D]

Trout Bog Lake August 11 2009 Epilimnion (Trout Bo ... st 11 2009 Epilimnion, new10pct subsample IHXC Merged Reads) (MER-FS) (*)[D]

Trout Bog Lake August 12 2008 Epilimnion (Trout Bo ... st 12 2008 Epilimnion, new10pct subsample IHTH Merged Reads) (MER-FS) (*)[D]

Trout Bog Lake August 18 2009 Epilimnion (Trout Bo ... st 18 2009 Epilimnion, new10pct subsample IHXB Merged Reads) (MER-FS) (*)[D]

Trout Bog Lake August 19 2008 Epilimnion (Trout Bo ... st 19 2008 Epilimnion, new10pct subsample IHTF Merged Reads) (MER-FS) (*)[D]

Trout Bog Lake August 20 2007 Epilimnion (Trout Bo ... st 20 2007 Epilimnion, new10pct subsample IHSG Merged Reads) (MER-FS) (*)[D]

Trout Bog Lake August 25 2008 Epilimnion (Trout Bo ... st 25 2008 Epilimnion, new10pct subsample IHTG Merged Reads) (MER-FS) (*)[D]

Trout Bog Lake August 27 2007 Epilimnion (Trout Bo ... st 27 2007 Epilimnion, new10pct subsample IHSF Merged Reads) (MER-FS) (*)[D]

Trout Bog Lake August 3 2009 Epilimnion (Trout Bog ... ust 3 2009 Epilimnion, new10pct subsample IHPS Merged Reads) (MER-FS) (*)[D]

Trout Bog Lake August 5 2008 Epilimnion (Trout Bog ... ust 5 2008 Epilimnion, new10pct subsample IHSZ Merged Reads) (MER-FS) (*)[D]

Trout Bog Lake August 9 2007 Epilimnion (Trout Bog ... ust 9 2007 Epilimnion, new10pct subsample IHSH Merged Reads) (MER-FS) (*)[D]

Trout Bog Lake July 1 2008 Epilimnion (Trout Bog L ... uly 1 2008 Epilimnion, new10pct subsample IHTO Merged Reads) (MER-FS) (*)[D]

Trout Bog Lake July 12 2007 Epilimnion (Trout Bog ... ly 12 2007 Epilimnion, new10pct subsample IHUY Merged Reads) (MER-FS) (*)[D]

Trout Bog Lake July 13 2009 Epilimnion (Trout Bog ... ly 13 2009 Epilimnion, new10pct subsample IHPW Merged Reads) (MER-FS) (*)[D]

Trout Bog Lake July 15 2008 Epilimnion (Trout Bog ... ly 15 2008 Epilimnion, new10pct subsample IHTA Merged Reads) (MER-FS) (*)[D]

Trout Bog Lake July 2 2007 Epilimnion (Trout Bog L ... uly 2 2007 Epilimnion, new10pct subsample IHUX Merged Reads) (MER-FS) (*)[D]

Trout Bog Lake July 21 2009 Epilimnion (Trout Bog ... ly 21 2009 Epilimnion, new10pct subsample IHPT Merged Reads) (MER-FS) (*)[D]

Trout Bog Lake July 22 2008 Epilimnion (Trout Bog ... ly 22 2008 Epilimnion, new10pct subsample IHTB Merged Reads) (MER-FS) (*)[D]

Trout Bog Lake July 25 2007 Epilimnion (Trout Bog ... ly 25 2007 Epilimnion, new10pct subsample IHWZ Merged Reads) (MER-FS) (*)[D]

Trout Bog Lake July 27 2009 Epilimnion (Trout Bog ... ly 27 2009 Epilimnion, new10pct subsample IHPP Merged Reads) (MER-FS) (*)[D]

Trout Bog Lake July 29 2008 Epilimnion (Trout Bog ... ly 29 2008 Epilimnion, new10pct subsample IHSY Merged Reads) (MER-FS) (*)[D]

Trout Bog Lake July 31 2007 Epilimnion (Trout Bog ... ly 31 2007 Epilimnion, new10pct subsample IHPZ Merged Reads) (MER-FS) (*)[D]

Trout Bog Lake July 7 2009 Epilimnion (Trout Bog L ... uly 7 2009 Epilimnion, new10pct subsample IHPX Merged Reads) (MER-FS) (*)[D]

Trout Bog Lake July 8 2008 Epilimnion (Trout Bog L ... 8 2008 Epilimnion, new10pct subsample of IHPI Merged Reads) (MER-FS) (*)[D]

Trout Bog Lake June 13 2007 Epilimnion (Trout Bog ... ne 13 2007 Epilimnion, new10pct subsample IHUT Merged Reads) (MER-FS) (*)[D]

Trout Bog Lake June 13 2008 Epilimnion (Trout Bog ... ne 13 2008 Epilimnion, new10pct subsample IHTI Merged Reads) (MER-FS) (*)[D]

Trout Bog Lake June 23 2009 Epilimnion (Trout Bog ... ne 23 2009 Epilimnion, new10pct subsample IHSN Merged Reads) (MER-FS) (*)[D]

Trout Bog Lake June 24 2008 Epilimnion (Trout Bog ... ne 24 2008 Epilimnion, new10pct subsample IHTN Merged Reads) (MER-FS) (*)[D]

Trout Bog Lake June 27 2007 Epilimnion (Trout Bog ... ne 27 2007 Epilimnion, new10pct subsample IHWC Merged Reads) (MER-FS) (*)[D]

Trout Bog Lake June 29 2009 Epilimnion (Trout Bog ... ne 29 2009 Epilimnion, new10pct subsample IHSI Merged Reads) (MER-FS) (*)[D]

Trout Bog Lake June 3 2009 Epilimnion (Trout Bog L ... une 3 2009 Epilimnion, new10pct subsample IHSW Merged Reads) (MER-FS) (*)[D]

Trout Bog Lake June 7 2007 Epilimnion (Trout Bog Epilimnion - Bur_E_0031_40) (MER-FS) (*)[D]

Trout Bog Lake June 7 2007 Epilimnion (Trout Bog Epilimnion - Bur_E_0116_55) (MER-FS) (*)[D]

Trout Bog Lake June 7 2007 Epilimnion (Trout Bog Epilimnion - Chl_Cvg0064_77) (MER-FS) (*)[D]

Trout Bog Lake June 7 2007 Epilimnion (Trout Bog Epilimnion - Chl_Cvg0330_53) (MER-FS) (*)[D]

Trout Bog Lake June 7 2007 Epilimnion (Trout Bog Epilimnion - Cvg064_78) (MER-FS) (*)[D]

Trout Bog Lake June 7 2007 Epilimnion (Trout Bog Epilimnion - Cvg328_58) (MER-FS) (*)[D]

Trout Bog Lake June 7 2007 Epilimnion (Trout Bog Epilimnion - Met_E_0024_73) (MER-FS) (*)[D]

Trout Bog Lake June 7 2007 Epilimnion (Trout Bog L ... une 7 2007 Epilimnion, new10pct subsample IHUW Merged Reads) (MER-FS) (*)[D]

Trout Bog Lake June 8 2009 Epilimnion (Trout Bog L ... une 8 2009 Epilimnion, new10pct subsample IHSS Merged Reads) (MER-FS) (*)[D]

Trout Bog Lake May 22 2008 Epilimnion (Trout Bog L ... ay 22 2008 Epilimnion, new10pct subsample IHTS Merged Reads) (MER-FS) (*)[D]

Trout Bog Lake May 29 2008 Epilimnion (Trout Bog L ... ay 29 2008 Epilimnion, new10pct subsample IHTT Merged Reads) (MER-FS) (*)[D]

Trout Bog Lake May 29 2009 Epilimnion (Trout Bog L ... ay 29 2009 Epilimnion, new10pct subsample IHSU Merged Reads) (MER-FS) (*)[D]

Trout Bog Lake November 14 2007 Epilimnion (Trout ... er 14 2007 Epilimnion, new10pct subsample IHTX Merged Reads) (MER-FS) (*)[D]

Trout Bog Lake November 5 2007 Epilimnion (Trout B ... ber 5 2007 Epilimnion, new10pct subsample IHTU Merged Reads) (MER-FS) (*)[D]

Trout Bog Lake October 1 2007 Epilimnion (Trout Bo ... ber 1 2007 Epilimnion, new10pct subsample IHTY Merged Reads) (MER-FS) (*)[D]

Trout Bog Lake October 16 2007 Epilimnion (Trout B ... er 16 2007 Epilimnion, new10pct subsample IHTW Merged Reads) (MER-FS) (*)[D]

Trout Bog Lake October 23 2008 Epilimnion (Trout B ... er 23 2008 Epilimnion, new10pct subsample IHSO Merged Reads) (MER-FS) (*)[D]

Trout Bog Lake October 4 2008 Epilimnion (Trout Bo ... ber 4 2008 Epilimnion, new10pct subsample IHSX Merged Reads) (MER-FS) (*)[D]

Trout Bog Lake September 17 2007 Epilimnion (Trout ... pt 17 2007 Epilimnion, new10pct subsample IHSC Merged Reads) (MER-FS) (*)[D]

Trout Bog Lake September 20 2008 Epilimnion (Trout ... pt 20 2008 Epilimnion, new10pct subsample IHTC Merged Reads) (MER-FS) (*)[D]

Trout Bog Lake September 9 2008 Epilimnion (Trout ... ber 9 2008 Epilimnion, new10pct subsample IHSP Merged Reads) (MER-FS) (*)[D]

Freshwater microbial communities from Trout Bog Lake, WI and Lake Mendota, IL

Freshwater microbial communities from Trout Bog La ... ce 03JUN2009 hypolimnion,new10pct subsampleHZHZ MergedReads) (MER-FS) (*)[D]

Freshwater microbial communities from Trout Bog La ... n (Trout Bog Practice 03JUN2009 hypolimnion June 2011 assem) (MER-FS) (*)[D]

Freshwater microbial communities from Trout Bog La ... n (Trout Bog Practice 03JUN2009 hypolimnion June 2011 assem) (MER-FS) (*)[D]

Freshwater microbial communities from Trout Bog La ... ce 18AUG2009 hypolimnion,new10pct subsampleHZIC MergedReads) (MER-FS) (*)[D]

Freshwater microbial communities from Trout Bog La ... n (Trout Bog Practice 18AUG2009 hypolimnion June 2011 assem) (MER-FS) (*)[D]

Freshwater microbial communities from Trout Bog La ... n (Trout Bog Practice 18AUG2009 hypolimnion June 2011 assem) (MER-FS) (*)[D]

Trout Bog Lake May 28 2007 Hypolimnion (Trout Bog ... ke Combined Assembly 47 Hypolimnion Samples, Aug 2012 Assem) (MER-FS) (*)[D]

Metagenomics and metatranscriptomics of the Lake Erie 'dead zone': a seasonal source of greenhouse gases

Lake Erie CCB hypolimnion July 2011 (Lake Erie CCB hypolimnion July 2011, ASSEMBLY_DATE=20121208) (MER-FS) (*)[D]

Freshwater microbial communities from Trout Bog Lake, WI and Lake Mendota, WI

TTrout Bog Lake June 27 2007 Hypolimnion (Trout Bo ... e 27 2007 Hypolimnion, new10pct subsample IHWI Merged Reads) (MER-FS) (*)[D]

Trout Bog Lake August 11 2009 Hypolimnion (Trout B ... t 11 2009 Hypolimnion, new10pct subsample IHUI Merged Reads) (MER-FS) (*)[D]

Trout Bog Lake August 12 2008 Hypolimnion (Trout B ... t 12 2008 Hypolimnion, new10pct subsample IHXF Merged Reads) (MER-FS) (*)[D]

Trout Bog Lake August 18 2009 Hypolimnion (Trout B ... t 18 2009 Hypolimnion, new10pct subsample IHUF Merged Reads) (MER-FS) (*)[D]

Trout Bog Lake August 19 2008 Hypolimnion (Trout B ... t 19 2008 Hypolimnion, new10pct subsample IHXU Merged Reads) (MER-FS) (*)[D]

Trout Bog Lake August 20 2007 Hypolimnion (Trout B ... t 20 2007 Hypolimnion, new10pct subsample IHWA Merged Reads) (MER-FS) (*)[D]

Trout Bog Lake August 25 2008 Hypolimnion (Trout B ... t 25 2008 Hypolimnion, new10pct subsample IHXS Merged Reads) (MER-FS) (*)[D]

Trout Bog Lake August 27 2007 Hypolimnion (Trout B ... t 27 2007 Hypolimnion, new10pct subsample IHWY Merged Reads) (MER-FS) (*)[D]

Trout Bog Lake August 3 2009 Hypolimnion (Trout Bo ... st 3 2009 Hypolimnion, new10pct subsample IHUH Merged Reads) (MER-FS) (*)[D]

Trout Bog Lake August 5 2008 Hypolimnion (Trout Bo ... st 5 2008 Hypolimnion, new10pct subsample IHXA Merged Reads) (MER-FS) (*)[D]

Trout Bog Lake August 9 2007 Hypolimnion (Trout Bo ... st 9 2007 Hypolimnion, new10pct subsample IHSA Merged Reads) (MER-FS) (*)[D]

Trout Bog Lake July 1 2008 Hypolimnion (Trout Bog ... ly 1 2008 Hypolimnion, new10pct subsample IHWX Merged Reads) (MER-FS) (*)[D]

Trout Bog Lake July 12 2007 Hypolimnion (Trout Bog ... y 12 2007 Hypolimnion, new10pct subsample IHWG Merged Reads) (MER-FS) (*)[D]

Trout Bog Lake July 13 2009 Hypolimnion (Trout Bog ... y 13 2009 Hypolimnion, new10pct subsample IHUA Merged Reads) (MER-FS) (*)[D]

Trout Bog Lake July 15 2008 Hypolimnion (Trout Bog ... y 15 2008 Hypolimnion, new10pct subsample IHXP Merged Reads) (MER-FS) (*)[D]

Trout Bog Lake July 2 2007 Hypolimnion (Trout Bog ... ly 2 2007 Hypolimnion, new10pct subsample IHWN Merged Reads) (MER-FS) (*)[D]

Trout Bog Lake July 21 2009 Hypolimnion (Trout Bog ... y 21 2009 Hypolimnion, new10pct subsample IHUB Merged Reads) (MER-FS) (*)[D]

Trout Bog Lake July 22 2008 Hypolimnion (Trout Bog ... y 22 2008 Hypolimnion, new10pct subsample IHXT Merged Reads) (MER-FS) (*)[D]

Trout Bog Lake July 25 2007 Hypolimnion (Trout Bog ... y 25 2007 Hypolimnion, new10pct subsample IHWH Merged Reads) (MER-FS) (*)[D]

Trout Bog Lake July 27 2009 Hypolimnion (Trout Bog ... y 27 2009 Hypolimnion, new10pct subsample IHUC Merged Reads) (MER-FS) (*)[D]

Trout Bog Lake July 29 2008 Hypolimnion (Trout Bog ... y 29 2008 Hypolimnion, new10pct subsample IHXH Merged Reads) (MER-FS) (*)[D]

Trout Bog Lake July 31 2007 Hypolimnion (Trout Bog ... y 31 2007 Hypolimnion, new10pct subsample IHUZ Merged Reads) (MER-FS) (*)[D]

Trout Bog Lake July 7 2009 Hypolimnion (Trout Bog ... ly 7 2009 Hypolimnion, new10pct subsample IHUP Merged Reads) (MER-FS) (*)[D]

Trout Bog Lake July 8 2008 Hypolimnion (Trout Bog ... ly 8 2008 Hypolimnion, new10pct subsample IHWW Merged Reads) (MER-FS) (*)[D]

Trout Bog Lake June 13 2007 Hypolimnion (Trout Bog ... e 13 2007 Hypolimnion, new10pct subsample IHUS Merged Reads) (MER-FS) (*)[D]

Trout Bog Lake June 13 2008 Hypolimnion (Trout Bog ... e 13 2008 Hypolimnion, new10pct subsample IHWS Merged Reads) (MER-FS) (*)[D]

Trout Bog Lake June 15 2009 Hypolimnion (Trout Bog ... e 15 2009 Hypolimnion, new10pct subsample IHXY Merged Reads) (MER-FS) (*)[D]

Trout Bog Lake June 23 2009 Hypolimnion (Trout Bog ... e 23 2009 Hypolimnion, new10pct subsample IHUN Merged Reads) (MER-FS) (*)[D]

Trout Bog Lake June 24 2008 Hypolimnion (Trout Bog ... e 24 2008 Hypolimnion, new10pct subsample IHWO Merged Reads) (MER-FS) (*)[D]

Trout Bog Lake June 29 2009 Hypolimnion (Trout Bog ... e 29 2009 Hypolimnion, new10pct subsample IHUO Merged Reads) (MER-FS) (*)[D]

Trout Bog Lake June 3 2009 Hypolimnion (Trout Bog ... ne 3 2009 Hypolimnion, new10pct subsample IHTZ Merged Reads) (MER-FS) (*)[D]

Trout Bog Lake June 7 2007 Hypolimnion (Trout Bog ... ne 7 2007 Hypolimnion, new10pct subsample IHWF Merged Reads) (MER-FS) (*)[D]

Trout Bog Lake May 22 2008 Hypolimnion (Trout Bog ... y 22 2008 Hypolimnion, new10pct subsample IHPY Merged Reads) (MER-FS) (*)[D]

Trout Bog Lake May 28 2007 Hypolimnion (Trout Bog Epilimnion - Cvg0066_78) (MER-FS) (*)[D]

Trout Bog Lake May 28 2007 Hypolimnion (Trout Bog Epilimnion - Cvg1280_67) (MER-FS) (*)[D]

Trout Bog Lake May 28 2007 Hypolimnion (Trout Bog Hypolimnion - Com_H_0063_70) (MER-FS) (*)[D]

Trout Bog Lake May 28 2007 Hypolimnion (Trout Bog Hypolimnion - Com_H_0130_88) (MER-FS) (*)[D]

Trout Bog Lake May 28 2007 Hypolimnion (Trout Bog Hypolimnion - Met_H_0124_42) (MER-FS) (*)[D]

Trout Bog Lake May 28 2007 Hypolimnion (Trout Bog Hypolimnion - Met_H_0330_34) (MER-FS) (*)[D]

Trout Bog Lake May 28 2007 Hypolimnion (Trout Bog ... y 28 2007 Hypolimnion, new10pct subsample IHXI Merged Reads) (MER-FS) (*)[D]

Trout Bog Lake May 29 2008 Hypolimnion (Trout Bog ... y 29 2008 Hypolimnion, new10pct subsample IHSB Merged Reads) (MER-FS) (*)[D]

Trout Bog Lake May 29 2009 Hypolimnion (Trout Bog ... y 29 2009 Hypolimnion, new10pct subsample IHWT Merged Reads) (MER-FS) (*)[D]

Trout Bog Lake November 14 2007 Hypolimnion (Trout ... v 14 2007 Hypolimnion, new10pct subsample IHXW Merged Reads) (MER-FS) (*)[D]

Trout Bog Lake November 5 2007 Hypolimnion (Trout ... er 5 2007 Hypolimnion, new10pct subsample IHXN Merged Reads) (MER-FS) (*)[D]

Trout Bog Lake October 1 2007 Hypolimnion (Trout B ... er 1 2007 Hypolimnion, new10pct subsample IHXG Merged Reads) (MER-FS) (*)[D]

Trout Bog Lake October 16 2007 Hypolimnion (Trout ... r 16 2007 Hypolimnion, new10pct subsample IHXO Merged Reads) (MER-FS) (*)[D]

Trout Bog Lake October 23 2008 Hypolimnion (Trout ... r 23 2008 Hypolimnion, new10pct subsample IHPN Merged Reads) (MER-FS) (*)[D]

Trout Bog Lake October 4 2008 Hypolimnion (Trout B ... er 4 2008 Hypolimnion, new10pct subsample IHWP Merged Reads) (MER-FS) (*)[D]

Trout Bog Lake September 10 2007 Hypolimnion (Trou ... t 10 2007 Hypolimnion, new10pct subsample IHWB Merged Reads) (MER-FS) (*)[D]

Trout Bog Lake September 17 2007 Hypolimnion (Trou ... t 17 2007 Hypolimnion, new10pct subsample IHWU Merged Reads) (MER-FS) (*)[D]

Trout Bog Lake September 20 2008 Hypolimnion (Trou ... t 20 2008 Hypolimnion, new10pct subsample IHPO Merged Reads) (MER-FS) (*)[D]

Trout Bog Lake September 9 2008 Hypolimnion (Trout ... pt 9 2008 Hypolimnion, new10pct subsample IHXX Merged Reads) (MER-FS) (*)[D]

Littoral zone

Photosynthetic Consortia grown using incandescent light

Lentic microbial communities from Lake Waban, Well ... osynthetic consortia incandescent light (FW_incandescent_CN) (MER-FS) (*)[D]

Photosynthetic Consortia 720nm

Lentic microbial communities from Wellesley MA, th ... ic and photosynthetic, sample Photosynthetic Consortia 720nm (MER-FS) (*)[D]

Sediment

Sediment methylotrophic communities from Lake Washington

Methylotrophic community from Lake Washington sediment Formaldehyde enrichment (*)[D]

Methylotrophic community from Lake Washington sediment Formate enrichment (MER-FS) (*)[D]

Methylotrophic community from Lake Washington sediment Methane enrichment (*)[D]

Methylotrophic community from Lake Washington sediment Methanol enrichment (*)[D]

Methylotrophic community from Lake Washington sediment Methylamine enrichment (*)[D]

Methylotrophic community from Lake Washington sediment combined (v2) (*)[D]

Sediment microbial communities from Lake Washington for Methane and Nitrogen Cycles

Sediment microbial communities from Lake Washingto ... for Methane and Nitrogen Cycles, original sample replicate 1 (MER-FS) (*)[D]

Sediment microbial communities from Lake Washingto ... for Methane and Nitrogen Cycles, original sample replicate 1 (MER-FS) (*)[D]

Sediment microbial communities from Lake Washingto ... s, original sample replicate 1 (Original sample replicate 1) (MER-FS) (*)[D]

Sediment microbial communities from Lake Washingto ... on-no added nitrate (Aerobic without added nitrate, 13C SIP) (MER-FS) (*)[D]

Sediment microbial communities from Lake Washingto ... ethane aerobic+nitrate (Aerobic with added nitrate, 13C SIP) (MER-FS) (*)[D]

Sediment microbial communities from Lake Washingto ... anaerobic+nitrate (Anaerobic + nitrate SIP Nov 2010 with PE) (MER-FS) (*)[D]

Sediment microbial communities from Lake Washingto ... ne anaerobic+nitrate (Anaerobic with added nitrate, 13C SIP) (MER-FS) (*)[D]

Sediment microbial communities from Lake Washingto ... Sediment Anaerobic+Nitrate 13C-methane SIP, Oct 2010 Assem) (MER-FS) (*)[D]

Sediment microbial communities from Lake Washingto ... robic no nitrate (Anaerobic no nitrate SIP Nov 2010 with PE) (MER-FS) (*)[D]

Sediment microbial communities from Lake Washingto ... erobic no nitrate (Anaerobic without added nitrate, 13C SIP) (MER-FS) (*)[D]

Sediment microbial communities from Lake Washingto ... ion (Aerobic without added nitrate, SIP additional fraction) (MER-FS) (*)[D]

Sediment microbial communities from Lake Washingto ... w sorted aerobic no nitrate (Flow sorted aerobic no nitrate) (MER-FS) (*)[D]

Sediment microbial communities from Lake Washingto ... rted aerobic plus nitrate (Flow sorted aerobic plus nitrate) (MER-FS) (*)[D]

Sediment microbial communities from Lake Washingto ... nitrate (Flow sorted anaerobic no nitrate Feb 2011 assembly) (MER-FS) (*)[D]

Sediment microbial communities from Lake Washingto ... flow sorted anaerobic no nitrate (WGA anaerobic no nitrate) (MER-FS) (*)[D]

Freshwater microbial communities from Lake Vostok at Ice accretion

Freshwater microbial communities from Lake Vostok at Ice accretion (5G Core, April 2011 assem (454, Ilumina combined)) (*)[D]

Lotic microbial communities from Mississippi River ... Minnesota, sample from River Site 1, Mississippi Headwaters (MER-FS) (*)[D]

Lotic microbial communities from Mississippi River ... a, sample from River Site 7, Mississippi Headwaters (Site 7) (MER-FS) (*)[D]

Sediment microbial communities from Lake Washington for Methane and Nitrogen Cycles

Sediment microbial communities from Lake Washingto ... ample replicate 2 (Original sample replicate 2 12C fraction) (MER-FS) (*)[D]

Baltic Sea Metagenome

Baltic Sea Metagenome (*)[D]

Microbial Interactions in Extremophilic Mat Communities

Joseph's Coat JC3_ASED Metagenome (Josephs Coat JC3_ASED Metagenome, ASSEMBLY_DATE=20130501) (MER-FS) (*)[D]

Monarch Geyser MG Metagenome (Monarch Geyser MG Metagenome, ASSEMBLY_DATE=20130420) (MER-FS) (*)[D]

Octopus OCT_B Metagenome (Octopus OCT_B Metagenome, ASSEMBLY_DATE=20130425) (MER-FS) (*)[D]

RS3B Metagenome (RS3B Metagenome, ASSEMBLY_DATE=20130423) (MER-FS) (*)[D]

Washburn Spring WS Metagenome (Washburn Spring WS Metagenome, ASSEMBLY_DATE=20130521) (MER-FS) (*)[D]

Washburn Spring WS Metagenome (Washburn Spring WS Metagenome, ASSEMBLY_DATE=20130521) (MER-FS) (*)[D]

Non-marine Saline and Alkaline Near Boling (>90C)

90C):::Alkaline:::' value='2007309000'>Bath Hot Springs, filamentous community (MER-FS) (*)[D]

90C):::Alkaline:::' value='2007309001'>Bath Hot Springs, planktonic community (MER-FS) (*)[D]

Saline

Sediment microbial community from Etoliko Lagoon, Greece

Benthic water microbial community from Etoliko Lagoon, Greece (Assembled contigs) (*)[D]

Benthic water microbial community from Etoliko Lagoon, Greece (Assembled contigs) (MER-FS) (*)[D]

Benthic water microbial community from Etoliko Lagoon, Greece (Assembled reads (mapped)) (MER-FS) (*)[D]

Benthic water microbial community from Etoliko Lagoon, Greece (Unassembled reads (unmapped)) (MER-FS) (*)[D]

Hypersaline water microbial communities from Lake Tyrrell, Victoria, Australia

Hypersaline water microbial communities from Lake Tyrrell, Victoria, Australia, sample J07AB- all scaffolds (MER-FS) (*)[D]

Saline water microbial communities from Great Salt Lake

Saline water microbial communities from Great Salt Lake, Utah, sample from North Arm Rozel Point (MER-FS) (*)[D]

Saline water microbial communities from Great Salt ... sample from North Arm Stromatolite (North Arm Stromatolite) (MER-FS) (*)[D]

Saline water microbial communities from Great Salt ... from South Arm Antelope Island 1 (South Arm Antelope Island) (MER-FS) (*)[D]

Saline water microbial communities from Great Salt ... sample from South Arm Stromatolite (South Arm Stromatolite) (MER-FS) (*)[D]

Environmental microbial communities from Soda lakes and Soda Solonchak soils

Soda Lake 4KL_010_BRINE (Soda Lake 4KL_010_BRINE, July 2012 Assem) (MER-FS) (*)[D]

Microbial mats

Saline water microbial communities from Elkhorn Slough hypersaline mats, California

Elkhorn Slough cyanobacterial mat Day (7am transcriptome CGUN) (MER-FS) (*)[D]

Elkhorn Slough cyanobacterial mat night (9pm Metatranscriptome CGUI) (MER-FS) (*)[D]

Hypersaline water microbial communities from Lyngbya mats, Guerrero Negro, Mexico and Elkhorn Slough mats, California, USA

Elkhorn Slough mat CD2A Metagenome (Elkhorn Slough mat CD2A, July 2012 Assem) (MER-FS) (*)[D]

Elkhorn Slough mat MD6A Metagenome (Elkhorn Slough mat MD6A, June 2012 Assem) (MER-FS) (*)[D]

Thalassic

Lake Vida Brine Hole Two >0.2 micron fraction (MER-FS) (*)[D]

Lake Vida Brine Hole Two >0.2 micron fraction (Lak ... Brine Hole Two >0.2 micron fraction, ASSEMBLY_DATE=20130302) (MER-FS) (*)[D]

Lake Vida Brine Hole Two >0.2 micron fraction (Lake Vida Brine Hole Two >0.2 micron fraction, Nov 2012 Assem) (MER-FS) (*)[D]

Sediment microbial community from Etoliko Lagoon, Greece

Sediment microbial community from Etoliko Lagoon, Greece (WGS sequence) (*)[D]

Sediment microbial community from Etoliko Lagoon, Greece (combined Illumina-454-Sanger assembly) (*)[D]

Salt crystallizer ponds

Saline water microbial communities from Guerrero Negro hypersaline mats

Guerrero Negro salt ponds hypersaline mat 01(G) (MER-FS) (*)[D]

Guerrero Negro salt ponds hypersaline mat 02(H) (MER-FS) (*)[D]

Guerrero Negro salt ponds hypersaline mat 03(I) (MER-FS) (*)[D]

Guerrero Negro salt ponds hypersaline mat 04(N) (MER-FS) (*)[D]

Guerrero Negro salt ponds hypersaline mat 05(O) (MER-FS) (*)[D]

Guerrero Negro salt ponds hypersaline mat 06(P) (MER-FS) (*)[D]

Guerrero Negro salt ponds hypersaline mat 07(S) (MER-FS) (*)[D]

Guerrero Negro salt ponds hypersaline mat 08(T) (MER-FS) (*)[D]

Guerrero Negro salt ponds hypersaline mat 09(Y) (MER-FS) (*)[D]

Guerrero Negro salt ponds hypersaline mat 10(Z) (MER-FS) (*)[D]

Alkaline

Lake Vida Brine Hole Two 0.1-0.2 micron fraction (... ida Brine Hole Two 0.1-0.2 micron fraction, July 2012 Assem) (MER-FS) (*)[D]

Environmental microbial communities from Soda lakes and Soda Solonchak soils

Soda Lake 5KL_010_BRINE (Soda Lake 5KL_010_BRINE, July 2012 Assem) (MER-FS) (*)[D]

Soda Lake 6KL_011_BRINE (Soda Lake 6KL_011_BRINE, March 2012 Assem) (MER-FS) (*)[D]

Soda Lake 7KL_010_BRINE (Soda Lake 7KL_010_BRINE, July 2012 Assem) (MER-FS) (*)[D]

Environmental microbial communities from Soda lakes and Soda Solonchak soils

Soda Lake 1KL_011_SED (Soda Lake 1KL_011_SED, April 2012 Assem) (MER-FS) (*)[D]

Soda Lake 3KL_010_SED (Soda Lake 3KL_010_SED, July 2012 Assem) (MER-FS) (*)[D]

Soda Lake 5KL_010_SED (Soda Lake 5KL_010_SED, March 2012 Assem) (MER-FS) (*)[D]

Soda Lake 8KL_010_SED (Soda Lake 8KL_010_SED, Oct 2012 Assem) (MER-FS) (*)[D]

Soda Lake 9KL_010_SED (Soda Lake 9KL_010_SED, May 2012 Assem) (MER-FS) (*)[D]

Soda Solonchak soil 2KS_010_SOIL (Soda Solonchak soil 2KS_010_SOIL, May 2012 Assem) (MER-FS) (*)[D]

Soda Solonchak soil 3KL_010_SOIL (Soda Solonchak soil 3KL_010_SOIL, May 2012 Assem) (MER-FS) (*)[D]

Air microbial communities from Singapore

Air microbial communities Singapore indoor air filters 1 (MER-FS) (*)[D]

Air microbial communities Singapore indoor air filters 2 (MER-FS) (*)[D]

Unclassified

Amazon Forest 2010 replicate II A001 (Amazon Forest 2010 replicate II A001, March 2012 Assem) (MER-FS) (*)[D]

Amazon Forest 2010 replicate II A01 (Amazon Forest 2010 replicate II A01, March 2012 Assem) (MER-FS) (*)[D]

Amazon Forest 2010 replicate II A1 (Amazon Forest 2010 replicate II A1, April 2012 Assem) (MER-FS) (*)[D]

Amazon Forest 2010 replicate II A10 (Amazon Forest 2010 replicate II A10, May 2012 Assem) (MER-FS) (*)[D]

Amazon Forest 2010 replicate II A100 (Amazon Forest 2010 replicate II A100, April 2012 Assem) (MER-FS) (*)[D]

Amazon Pasture72 2010 replicate I A001 (Amazon Pasture72 2010 replicate I A001, May 2012 Assem) (MER-FS) (*)[D]

Amazon Pasture72 2010 replicate I A01 (Amazon Pasture72 2010 replicate I A01, April 2012 Assem) (MER-FS) (*)[D]

Amazon Pasture72 2010 replicate I A1 (Amazon Pasture72 2010 replicate I A1, April 2012 Assem) (MER-FS) (*)[D]

Amazon Pasture72 2010 replicate I A10 (Amazon Pasture72 2010 replicate I A10, April 2012 Assem) (MER-FS) (*)[D]

Amazon Pasture72 2010 replicate I A100 (Amazon Pasture72 2010 replicate I A100, April 2012 Assem) (MER-FS) (*)[D]

Microbial communities from Arabidopsis rhizosphere

Arabidopsis rhizosphere microbial communities from ... Arabidopsis soil old (Arabidopsis soil old, Nov 2011 assem) (MER-FS) (*)[D]

Cross-site metagenomic analyses to assess the impacts of experimental nitrogen additions on belowground carbon dynamics

Forest Soil microbial communities with Nitrogen NN ... ial communities with Nitrogen NN102, ASSEMBLY_DATE=20130604) (MER-FS) (*)[D]

Forest Soil microbial communities with Nitrogen NN ... ial communities with Nitrogen NN332, ASSEMBLY_DATE=20130213) (MER-FS) (*)[D]

Forest Soil microbial communities with Nitrogen NN ... ial communities with Nitrogen NN335, ASSEMBLY_DATE=20130213) (MER-FS) (*)[D]

Forest Soil microbial communities with Nitrogen NN ... ial communities with Nitrogen NN336, ASSEMBLY_DATE=20130213) (MER-FS) (*)[D]

Forest Soil microbial communities with Nitrogen NN ... ial communities with Nitrogen NN338, ASSEMBLY_DATE=20130220) (MER-FS) (*)[D]

Forest Soil microbial communities with Nitrogen NN ... ial communities with Nitrogen NN342, ASSEMBLY_DATE=20130213) (MER-FS) (*)[D]

Forest Soil microbial communities with Nitrogen NN ... ial communities with Nitrogen NN344, ASSEMBLY_DATE=20130213) (MER-FS) (*)[D]

Forest Soil microbial communities with Nitrogen NN ... ial communities with Nitrogen NN346, ASSEMBLY_DATE=20130213) (MER-FS) (*)[D]

Forest Soil microbial communities with Nitrogen NN ... ial communities with Nitrogen NN349, ASSEMBLY_DATE=20130221) (MER-FS) (*)[D]

Forest Soil microbial communities with Nitrogen NN ... ial communities with Nitrogen NN350, ASSEMBLY_DATE=20130213) (MER-FS) (*)[D]

Forest Soil microbial communities with Nitrogen NN ... ial communities with Nitrogen NN351, ASSEMBLY_DATE=20130213) (MER-FS) (*)[D]

Forest Soil microbial communities with Nitrogen NN ... ial communities with Nitrogen NN352, ASSEMBLY_DATE=20130213) (MER-FS) (*)[D]

Forest Soil microbial communities with Nitrogen NN ... ial communities with Nitrogen NN356, ASSEMBLY_DATE=20130213) (MER-FS) (*)[D]

Forest Soil microbial communities with Nitrogen NN ... ial communities with Nitrogen NN357, ASSEMBLY_DATE=20130213) (MER-FS) (*)[D]

Forest soil microbial communities from Douglas fir zone in British Columbia and Gulf Coastal Plain in Texas

Mediterranean Blodgett CA OM1_M1 (Mediterranean Blodgett CA OM1_M1, ASSEMBLY_DATE=20130115) (MER-FS) (*)[D]

Mediterranean Blodgett CA OM1_M1 (Mediterranean Blodgett CA OM1_M1, Jul 2012 Assem) (MER-FS) (*)[D]

Mediterranean Blodgett CA OM1_M2 (Mediterranean Blodgett CA OM1_M2, ASSEMBLY_DATE=20130528) (MER-FS) (*)[D]

Mediterranean Blodgett CA OM1_M2 (Mediterranean Blodgett CA OM1_M2, June 2012 Assem) (MER-FS) (*)[D]

Mediterranean Blodgett CA OM1_M3 (Mediterranean Blodgett CA OM1_M3, ASSEMBLY_DATE=20130227) (MER-FS) (*)[D]

Mediterranean Blodgett CA OM1_O1 (Mediterranean Blodgett CA OM1_O1, ASSEMBLY_DATE=20130221) (MER-FS) (*)[D]

Mediterranean Blodgett CA OM1_O1 (Mediterranean Blodgett CA OM1_O1, June 2012 Assem) (MER-FS) (*)[D]

Mediterranean Blodgett CA OM1_O2 (Mediterranean Blodgett CA OM1_O2, ASSEMBLY_DATE=20130221) (MER-FS) (*)[D]

Mediterranean Blodgett CA OM1_O2 (Mediterranean Blodgett CA OM1_O2, June 2012 Assem) (MER-FS) (*)[D]

Mediterranean Blodgett CA OM1_O3 (Mediterranean Blodgett CA OM1_O3, ASSEMBLY_DATE=20130115) (MER-FS) (*)[D]

Mediterranean Blodgett CA OM1_O3 (Mediterranean Blodgett CA OM1_O3, June 2012 Assem) (MER-FS) (*)[D]

Mediterranean Blodgett CA OM1_O3 (Mediterranean Blodgett coassembly) (MER-FS) (*)[D]

Mediterranean Blodgett CA OM2_M2 (Mediterranean Blodgett CA OM2_M2, ASSEMBLY_DATE=20130222) (MER-FS) (*)[D]

Mediterranean Blodgett CA OM2_M2 (Mediterranean Blodgett CA OM2_M2, July 2012 Assem) (MER-FS) (*)[D]

Mediterranean Blodgett CA OM2_M3 (Mediterranean Blodgett CA OM2_M3, ASSEMBLY_DATE=20130115) (MER-FS) (*)[D]

Mediterranean Blodgett CA OM2_M3 (Mediterranean Blodgett CA OM2_M3, July 2012 Assem) (MER-FS) (*)[D]

Mediterranean Blodgett CA OM2_O1 (Mediterranean Blodgett CA OM2_O1, ASSEMBLY_DATE=20130227) (MER-FS) (*)[D]

Mediterranean Blodgett CA OM2_O1 (Mediterranean Blodgett CA OM2_O1, ASSEMBLY_DATE=20130227) (MER-FS) (*)[D]

Mediterranean Blodgett CA OM3_M1 (Mediterranean Blodgett CA OM3_M1, ASSEMBLY_DATE=20130222) (MER-FS) (*)[D]

Mediterranean Blodgett CA OM3_M1 (Mediterranean Blodgett CA OM3_M1, June 2012 Assem) (MER-FS) (*)[D]

Mediterranean Blodgett CA OM3_M3 (Mediterranean Blodgett CA OM3_M3, ASSEMBLY_DATE=20130227) (MER-FS) (*)[D]

Mediterranean Blodgett CA OM3_M3 (Mediterranean Blodgett CA OM3_M3, June 2012 Assem) (MER-FS) (*)[D]

Mediterranean Blodgett CA Ref_M1 (Mediterranean Blodgett CA Ref_M1, ASSEMBLY_DATE=20130115) (MER-FS) (*)[D]

Mediterranean Blodgett CA Ref_M1 (Mediterranean Blodgett CA Ref_M1, June 2012 Assem) (MER-FS) (*)[D]

Mediterranean Blodgett CA Ref_M2 (Mediterranean Blodgett CA Ref_M2, ASSEMBLY_DATE=20130115) (MER-FS) (*)[D]

Mediterranean Blodgett CA Ref_M2 (Mediterranean Blodgett CA Ref_M2, June 2012 Assem) (MER-FS) (*)[D]

Mediterranean Blodgett CA Ref_M3 (Mediterranean Blodgett CA Ref_M3, ASSEMBLY_DATE=20130115) (MER-FS) (*)[D]

Mediterranean Blodgett CA Ref_M3 (Mediterranean Blodgett CA Ref_M3, June 2012 Assem) (MER-FS) (*)[D]

Mediterranean Blodgett CA Ref_O1 (Mediterranean Blodgett CA Ref_O1, ASSEMBLY_DATE=20121222) (MER-FS) (*)[D]

Mediterranean Blodgett CA Ref_O1 (Mediterranean Blodgett CA Ref_O1, June 2012 Assem) (MER-FS) (*)[D]

Mediterranean Blodgett CA Ref_O2 (Mediterranean Blodgett CA Ref_O2, ASSEMBLY_DATE=20130115) (MER-FS) (*)[D]

Mediterranean Blodgett CA Ref_O2 (Mediterranean Blodgett CA Ref_O2, June 2012 Assem) (MER-FS) (*)[D]

Targeted metagenomics and metatranscriptomics of a sulfate-reducing rare biosphere member and potentially novel sulfate reducers that impact methane emission from peatlands

Peat Soil Metagenome SII-2010 (Peat Soil Metagenome SII-2010, Oct 2012 Assem) (MER-FS) (*)[D]

Peat Soil Metagenome SII-SIP-2007 (Peat Soil Metagenome SII-SIP-2007, ASSEMBLY_DATE=20130416) (MER-FS) (*)[D]

Soil microbial communities from Waseca County, Minnesota Farm

Soil microbial communities from Minnesota Farm (MER-FS) (*)[D]

Soil microbial communities from FACE and OTC sites

Soil microbial communities from sample at FACE Site North Carolina NCD_ElevF (NCD_ElevF) (MER-FS) (*)[D]

Soil microbial communities from sample at FACE Site 1 Maryland Estuary CO2+ (Maryland Estuary elevated) (MER-FS) (*)[D]

Soil microbial communities from sample at FACE Site 1 Maryland Estuary CO2- (Maryland Estuary ambient) (MER-FS) (*)[D]

Soil microbial communities from sample at FACE Site 2 North Carolina CO2+ (North Carolina Elevated CO2) (MER-FS) (*)[D]

Soil microbial communities from sample at FACE Site 2 North Carolina CO2- (MER-FS) (*)[D]

Soil microbial communities from sample at FACE Site 5 Oak Ridge CO2+ (Oak Ridge elevated CO2) (MER-FS) (*)[D]

Soil microbial communities from sample at FACE Site 5 Oak Ridge CO2- (Oak Ridge ambient) (MER-FS) (*)[D]

Soil microbial communities from sample at FACE Site Metagenome WIR_Amb2 (WIR_Amb2) (MER-FS) (*)[D]

Soil microbial communities from sample at FACE Site Metagenome WIR_Elev2 (WIR_Elev2) (MER-FS) (*)[D]

Soil microbial communities from sample at FACE Site Metagenome WIR_ElevOz2 (WIR_ElevOz2) (MER-FS) (*)[D]

Soil microbial communities from sample at FACE Site Metagenome WIR_Oz2 (WIR_Oz2) (MER-FS) (*)[D]

Soil microbial communities from sample at FACE Site North Carolina NCD_AmbF (NCD_AmbF) (MER-FS) (*)[D]

Switchgrass rhizosphere microbial community from Michigan, US

Switchgrass rhizosphere microbial community from M ... Rose Lake bulk soil RL2 (Bulk soil RL2 April 2011 assembly) (MER-FS) (*)[D]

Switchgrass rhizosphere microbial community from M ... bulk soil RL2 (Bulk soil RL2 January 2011 combined assembly) (MER-FS) (*)[D]

Switchgrass rhizosphere microbial community from M ... bulk soil RL3 (Bulk soil RL3 January 2011 combined assembly) (MER-FS) (*)[D]

Metagenomic and metatranscriptomic analysis of forest soil communities across North America

Texas A ecozone_OM3H0_O2 (Texas A ecozone_OM3H0_O2, ASSEMBLY_DATE=20130221) (MER-FS) (*)[D]

Texas A ecozone_OM3H0_O3 (Texas A ecozone_OM3H0_O3, ASSEMBLY_DATE=20130529) (MER-FS) (*)[D]

Texas A ecozone_RefH0_M1 (Texas A ecozone_RefH0_M1, ASSEMBLY_DATE=20130221) (MER-FS) (*)[D]

Texas A ecozone_RefH0_M2 (Texas A ecozone_RefH0_M2, ASSEMBLY_DATE=20130221) (MER-FS) (*)[D]

Texas A ecozone_RefH0_M3 (Texas A ecozone_RefH0_M3, ASSEMBLY_DATE=20130226) (MER-FS) (*)[D]

Texas A ecozone_RefH0_O1 (Texas A ecozone_RefH0_O1, ASSEMBLY_DATE=20130227) (MER-FS) (*)[D]

Texas A ecozone_RefH0_O2 (Texas A ecozone_RefH0_O2, ASSEMBLY_DATE=20130221) (MER-FS) (*)[D]

Texas A ecozone_RefH0_O3 (Texas A ecozone_RefH0_O3, ASSEMBLY_DATE=20130226) (MER-FS) (*)[D]

Grasslands

Soil microbial communities from Kansas Great Prairie, that have been labeled with BrdU

Kansas native prairie Total DNA no BrdU F1.4TC (Ka ... s native prairie Total DNA no BrdU F1.4TC, April 2012 Assem) (MER-FS) (*)[D]

Kansas native prairie amended with acetate Total D ... airie amended with acetate Total DNA F2.4TB, Dec 2011 Assem) (MER-FS) (*)[D]

Replicate Kansas native prairie amended with BrdU ... sas native prairie amended with BrdU F1.2B, June 2012 Assem) (MER-FS) (*)[D]

Replicate Kansas native prairie amended with aceta ... rairie amended with acetate and BrdU F2.1B, Jun 2012 Assem) (MER-FS) (*)[D]

Soil microbial communities from Miscanthus in Kellogg Biological Station, MSU

Miscanthus rhizosphere microbial communities from ... Soil Replicate 1 : eDNA_1 (Bulk soil 1 April 2011 assembly) (MER-FS) (*)[D]

Miscanthus rhizosphere microbial communities from Kellogg Bi ... cate 1 : eDNA_1 (Bulk soil 1 January 2011 combined assembly) (*)[D]

Miscanthus rhizosphere microbial communities from ... cate 2: eDNA_1 (Bulk soil 2 January 2011 combined assembly) (MER-FS) (*)[D]

Soil microbial communities from Great Prairies (Kansas, Wisconsin and Iowa)

Soil microbial communities from Great Prairies, sa ... e from Iowa, Continuous Corn soil (Iowa Continuous Corn 454) (MER-FS) (*)[D]

Soil microbial communities from Great Prairies, sa ... Iowa, Continuous Corn soil (Iowa Corn - 2 lanes GAIIx reads) (MER-FS) (*)[D]

Soil microbial communities from Great Prairies, sa ... Iowa, Continuous Corn soil (Iowa corn MSU Illumina assembly) (MER-FS) (*)[D]

Soil microbial communities from Great Prairies, sa ... (Iowa, Continuous Corn soil, Feb 2012 Assem MSU hiseq+gaii) (MER-FS) (*)[D]

Soil microbial communities from Great Prairies, sa ... (Iowa, Continuous Corn soil, Jan 2012 Assem MSU hiseq+gaii) (MER-FS) (*)[D]

Soil microbial communities from Great Prairies, sa ... n soil (Iowa, Continuous Corn soil, Oct 2011 Assem MSU gaii) (MER-FS) (*)[D]

Soil microbial communities from Great Prairies, sa ... (Iowa, Continuous Corn soil, Oct 2012 Assem MSU hiseq+gaii) (MER-FS) (*)[D]

Soil microbial communities from Great Prairies, sa ... il (Iowa, Continuous Corn soil, Sept. 2012 Assem JGI Velvet) (MER-FS) (*)[D]

Soil microbial communities from Great Prairies, sa ... ive Prairie soil (Iowa Native Prairie - 2 lanes GAIIx reads) (MER-FS) (*)[D]

Soil microbial communities from Great Prairies, sa ... ple from Iowa, Native Prairie soil (Iowa Native Prairie 454) (MER-FS) (*)[D]

Soil microbial communities from Great Prairies, sa ... ive Prairie soil (Iowa native prairie MSU Illumina assembly) (MER-FS) (*)[D]

Soil microbial communities from Great Prairies, sa ... l (Iowa, Native Prairie soil, Feb 2012 Assem MSU hiseq+gaii) (MER-FS) (*)[D]

Soil microbial communities from Great Prairies, sa ... ie soil (Iowa, Native Prairie soil, Oct 2011 Assem MSU gaii) (MER-FS) (*)[D]

Soil microbial communities from Great Prairies, sa ... l (Iowa, Native Prairie soil, Oct 2012 Assem MSU hiseq+gaii) (MER-FS) (*)[D]

Soil microbial communities from Great Prairies, sa ... soil (Iowa, Native Prairie soil, Oct. 2012 Assem JGI Velvet) (MER-FS) (*)[D]

Soil microbial communities from Great Prairies, sa ... le from Kansas Corn soil (Kansas Corn - 2 lanes GAIIx reads) (MER-FS) (*)[D]

Soil microbial communities from Great Prairies, sample from Kansas Corn soil (Kansas Corn) (MER-FS) (*)[D]

Soil microbial communities from Great Prairies, sa ... (Kansas, Cultivated Corn soil, Sept. 2012 Assem JGI Velvet) (MER-FS) (*)[D]

Soil microbial communities from Great Prairies, sample from Kansas, Native Prairie soil (MER-FS) (*)[D]

Soil microbial communities from Great Prairies, sa ... e Prairie soil (Kansas Native Prairie - 2 lanes GAIIx reads) (MER-FS) (*)[D]

Soil microbial communities from Great Prairies, sa ... ple from Kansas, Native Prairie soil (Kansas Native Prairie) (MER-FS) (*)[D]

Soil microbial communities from Great Prairies, sa ... as, Native Prairie soil (Kansas prairie abyss kmer depth >8) (MER-FS) (*)[D]

Soil microbial communities from Great Prairies, sa ... s, Native Prairie soil (Kansas prairie velvet kmer depth >8) (MER-FS) (*)[D]

Soil microbial communities from Great Prairies, sa ... l (Kansas, Native Prairie soil, Sept. 2012 Assem JGI Velvet) (MER-FS) (*)[D]

Soil microbial communities from Great Prairies, sample from Wisconsin Native Prairie soil (MER-FS) (*)[D]

Soil microbial communities from Great Prairies, sa ... rairie soil (Wisconsin Native Prairie - 2 lanes GAIIx reads) (MER-FS) (*)[D]

Soil microbial communities from Great Prairies, sa ... Wisconsin Native Prairie soil (Wisconsin Native Prairie 454) (MER-FS) (*)[D]

Soil microbial communities from Great Prairies, sa ... (Wisconsin, Native Prairie soil, Oct. 2012 Assem JGI Velvet) (MER-FS) (*)[D]

Soil microbial communities from Great Prairies, sa ... irie soil (Wisconsin Restored Prairie - 2 lanes GAIIx reads) (MER-FS) (*)[D]

Soil microbial communities from Great Prairies, sa ... Wisconsin Restored Prairie soil (Wisconsin Restored Prairie) (MER-FS) (*)[D]

Soil microbial communities from Great Prairies, sa ... isconsin, Restored Prairie soil, Oct. 2012 Assem JGI Velvet) (MER-FS) (*)[D]

Soil microbial communities from Great Prairies, sa ... Continuous corn soil (Wisconsin Corn - 2 lanes GAIIx reads) (MER-FS) (*)[D]

Soil microbial communities from Great Prairies, sa ... Wisconsin, Continuous Corn soil, Oct. 2012 Assem JGI Velvet) (MER-FS) (*)[D]

Soil microbial communities from Great Prairies, sa ... itchgrass soil (Wisconsin Switchgrass - 2 lanes GAIIx reads) (MER-FS) (*)[D]

Soil microbial communities from Great Prairies, sa ... oil (Wisconsin Switchgrass soil, Feb. 2013 Assem JGI Velvet) (MER-FS) (*)[D]

Soil microbial communities from Great Prairies, sa ... ple from Wisconsin, Switchgrass soil (Wisconsin Switchgrass) (MER-FS) (*)[D]

Soil microbial communities from Kansas, sample fro ... ce (Kansas native prairie Total DNA F1.4 TB, Feb 2012 Assem) (MER-FS) (*)[D]

Soil microbial communities from Kansas, sample fro ... h BrdU and acetate no abondance (no abundance, no trimmed.) (MER-FS) (*)[D]

Soil microbial communities from Kansas, sample fro ... e prairie amended with acetate & BrdU F2.3B, Jan 2012 Assem) (MER-FS) (*)[D]

Uncharacterized

Forest soil microbial communities from Douglas fir zone in British Columbia and Gulf Coastal Plain in Texas

Mediterranean Blodgett CA OM2_O3 (Mediterranean Blodgett CA OM2_O3, ASSEMBLY_DATE=20130221) (MER-FS) (*)[D]

Mediterranean Blodgett CA OM2_O3 (Mediterranean Blodgett CA OM2_O3, July 2012 Assem) (MER-FS) (*)[D]

Permafrost

Next Generation Ecosystem Experiment (NGEE) in the arctic

NGEE Surface sample 210 deep-092012 (NGEE Surface sample 210 deep-092012, ASSEMBLY_DATE=20130514) (MER-FS) (*)[D]

NGEE Surface sample 210 shallow-092012 (NGEE Surface sample 210 shallow-092012, ASSEMBLY_DATE=20130516) (MER-FS) (*)[D]

NGEE Surface sample 210-1 deep-072012 (NGEE Surface sample 210-1 deep-072012, ASSEMBLY_DATE=20130514) (MER-FS) (*)[D]

NGEE Surface sample 210-1 shallow-072012 (NGEE Surface sample 210-1 shallow-072012, ASSEMBLY_DATE=20130514) (MER-FS) (*)[D]

NGEE Surface sample 210-2 deep-072012 (NGEE Surface sample 210-2 deep-072012, ASSEMBLY_DATE=20130514) (MER-FS) (*)[D]

NGEE Surface sample 210-2 deep-092012 (NGEE Surface sample 210-2 deep-092012, ASSEMBLY_DATE=20130516) (MER-FS) (*)[D]

NGEE Surface sample 210-2 shallow-072012 (NGEE Surface sample 210-2 shallow-072012, ASSEMBLY_DATE=20130514) (MER-FS) (*)[D]

NGEE Surface sample 210-2 shallow-092012 (NGEE Surface sample 210-2 shallow-092012, ASSEMBLY_DATE=20130509) (MER-FS) (*)[D]

NGEE Surface sample 210-3 deep-072012 (NGEE Surface sample 210-3 deep-072012, ASSEMBLY_DATE=20130514) (MER-FS) (*)[D]

NGEE Surface sample 210-3 deep-092012 (NGEE Surface sample 210-3 deep-092012, ASSEMBLY_DATE=20130514) (MER-FS) (*)[D]

NGEE Surface sample 210-3 shallow-072012 (NGEE Surface sample 210-3 shallow-072012, ASSEMBLY_DATE=20130514) (MER-FS) (*)[D]

NGEE Surface sample 210-3 shallow-092012 (NGEE Surface sample 210-3 shallow-092012, ASSEMBLY_DATE=20130514) (MER-FS) (*)[D]

NGEE Surface sample 415-1 deep-072012 (NGEE Surface sample 415-1 deep-072012, ASSEMBLY_DATE=20130516) (MER-FS) (*)[D]

NGEE Surface sample 415-1 deep-092012 (NGEE Surface sample 415-1 deep-092012, ASSEMBLY_DATE=20130524) (MER-FS) (*)[D]

NGEE Surface sample 415-1 deep-092012 (NGEE Surface sample 415-1 deep-092012, ASSEMBLY_DATE=20130524) (MER-FS) (*)[D]

NGEE Surface sample 415-1 shallow-072012 (NGEE Surface sample 415-1 shallow-072012, ASSEMBLY_DATE=20130514) (MER-FS) (*)[D]

NGEE Surface sample 415-1 shallow-092012 (NGEE Surface sample 415-1 shallow-092012, ASSEMBLY_DATE=20130523) (MER-FS) (*)[D]

NGEE Surface sample 415-2 deep-072012 (NGEE Surface sample 415-2 deep-072012, ASSEMBLY_DATE=20130514) (MER-FS) (*)[D]

NGEE Surface sample 415-2 deep-092012 (NGEE Surface sample 415-2 deep-092012, ASSEMBLY_DATE=20130523) (MER-FS) (*)[D]

NGEE Surface sample 415-2 shallow-072012 (NGEE Surface sample 415-2 shallow-072012, ASSEMBLY_DATE=20130516) (MER-FS) (*)[D]

NGEE Surface sample 415-2 shallow-092012 (NGEE Surface sample 415-2 shallow-092012, ASSEMBLY_DATE=20130523) (MER-FS) (*)[D]

NGEE Surface sample 415-3 deep-072012 (NGEE Surface sample 415-3 deep-072012, ASSEMBLY_DATE=20130516) (MER-FS) (*)[D]

NGEE Surface sample 415-3 deep-092012 (NGEE Surface sample 415-3 deep-092012, ASSEMBLY_DATE=20130523) (MER-FS) (*)[D]

NGEE Surface sample 415-3 shallow-072012 (NGEE Surface sample 415-3 shallow-072012, ASSEMBLY_DATE=20130516) (MER-FS) (*)[D]

NGEE Surface sample 415-3 shallow-092012 (NGEE Surface sample 415-3 shallow-092012, ASSEMBLY_DATE=20130523) (MER-FS) (*)[D]

NGEE Surface sample 53-1 deep-072012 (NGEE Surface sample 53-1 deep-072012, ASSEMBLY_DATE=20130514) (MER-FS) (*)[D]

NGEE Surface sample 53-1 shallow-072012 (NGEE Surface sample 53-1 shallow-072012, ASSEMBLY_DATE=20130514) (MER-FS) (*)[D]

NGEE Surface sample 53-2 deep-072012 (NGEE Surface sample 53-2 deep-072012, ASSEMBLY_DATE=20130514) (MER-FS) (*)[D]

NGEE Surface sample 53-2 shallow-072012 (NGEE Surface sample 53-2 shallow-072012, ASSEMBLY_DATE=20130514) (MER-FS) (*)[D]

NGEE Surface sample 53-3 deep-072012 (NGEE Surface sample 53-3 deep-072012, ASSEMBLY_DATE=20130516) (MER-FS) (*)[D]

NGEE Surface sample 53-3 shallow-072012 (NGEE Surface sample 53-3 shallow-072012, ASSEMBLY_DATE=20130514) (MER-FS) (*)[D]

NGEE Surface sample F52-2 deep-092012 (NGEE Surface sample F52-2 deep-092012, ASSEMBLY_DATE=20130514) (MER-FS) (*)[D]

NGEE Surface sample F53-1 deep-092012 (NGEE Surface sample F53-1 deep-092012, ASSEMBLY_DATE=20130523) (MER-FS) (*)[D]

NGEE Surface sample F53-1 shallow-092012 (NGEE Surface sample F53-1 shallow-092012, ASSEMBLY_DATE=20130524) (MER-FS) (*)[D]

NGEE Surface sample F53-1 shallow-092012 (NGEE Surface sample F53-1 shallow-092012, ASSEMBLY_DATE=20130524) (MER-FS) (*)[D]

NGEE Surface sample F53-2 shallow-092012 (NGEE Surface sample F53-2 shallow-092012, ASSEMBLY_DATE=20130514) (MER-FS) (*)[D]

NGEE Surface sample F53-3 deep-092012 (NGEE Surface sample F53-3 deep-092012, ASSEMBLY_DATE=20130516) (MER-FS) (*)[D]

NGEE Surface sample F53-3 shallow (NGEE Surface sample F53-3 shallow, ASSEMBLY_DATE=20130516) (MER-FS) (*)[D]

Soil microbial communities from permafrost in Bonanza Creek, Alaska

Permafrost metatranscriptome cDNA-P1 (MER-FS) (*)[D]

Permafrost metatranscriptome cDNA-P3 (MER-FS) (*)[D]

Soil microbial communities from permafrost in Bonanza Creek, Alaska, sample from Active Layer A2 (A2_CLC_pe) (MER-FS) (*)[D]

Soil microbial communities from permafrost in Bona ... Creek, Alaska, sample from Active Layer A2 (A2_first_velvet) (MER-FS) (*)[D]

Soil microbial communities from permafrost in Bonanza Creek, Alaska, sample from Active Layer A2 (MG-A2-I) (MER-FS) (*)[D]

Soil microbial communities from permafrost in Bonanza Creek, Alaska, sample from Active Layer A2 (MT-A2-I) (MER-FS) (*)[D]

Soil microbial communities from permafrost in Bonanza Creek, Alaska, sample from Active Layer A5 (MER-FS) (*)[D]

Soil microbial communities from permafrost in Bonanza Creek, Alaska, sample from Active Layer A5 (A5_CLC_pe) (MER-FS) (*)[D]

Soil microbial communities from permafrost in Bona ... laska, sample from Active Layer A5 (Active_layer_A5_velvet1) (MER-FS) (*)[D]

Soil microbial communities from permafrost in Bonanza Creek, Alaska, sample from Active Layer A5 (MG-A5-I) (MER-FS) (*)[D]

Soil microbial communities from permafrost in Bonanza Creek, Alaska, sample from Active Layer A5 (MG-A5-II) (MER-FS) (*)[D]

Soil microbial communities from permafrost in Bonanza Creek, Alaska, sample from Active Layer A5 (MT-A5-I) (MER-FS) (*)[D]

Soil microbial communities from permafrost in Bonanza Creek, Alaska, sample from Bog Site B3 (MER-FS) (*)[D]

Soil microbial communities from permafrost in Bonanza Creek, Alaska, sample from Bog Site B3 (B3_GZBA_CLC) (MER-FS) (*)[D]

Soil microbial communities from permafrost in Bonanza Creek, Alaska, sample from Bog Site B3 (B3_GZCA_v) (MER-FS) (*)[D]

Soil microbial communities from permafrost in Bonanza Creek, Alaska, sample from Bog Site B3 (B3_GZOS) (MER-FS) (*)[D]

Soil microbial communities from permafrost in Bona ... a Creek, Alaska, sample from Bog Site B3 (B3_GZOS_Velvetvs2) (MER-FS) (*)[D]

Soil microbial communities from permafrost in Bonanza Creek, Alaska, sample from Bog Site B3 (B3_all_CLC) (MER-FS) (*)[D]

Soil microbial communities from permafrost in Bona ... za Creek, Alaska, sample from Bog Site B3 (B3_noscaf_velvet) (MER-FS) (*)[D]

Soil microbial communities from permafrost in Bonanza Creek, Alaska, sample from Bog Site B3 (MT-B3-I) (MER-FS) (*)[D]

Soil microbial communities from permafrost in Bona ... , Alaska, sample from Bog Site B3 (Velvet_1st_assembly_BZBA) (MER-FS) (*)[D]

Soil microbial communities from permafrost in Bonanza Creek, Alaska, sample from Bog Site B4 (B4_CLC) (MER-FS) (*)[D]

Soil microbial communities from permafrost in Bonanza Creek, Alaska, sample from Bog Site B4 (B4_velvet) (MER-FS) (*)[D]

Soil microbial communities from permafrost in Bonanza Creek, Alaska, sample from Bog Site B4 (MT-B4-I) (MER-FS) (*)[D]

Soil microbial communities from permafrost in Bona ... ek, Alaska, sample from Permafrost Layer P1 (First assembly) (MER-FS) (*)[D]

Soil microbial communities from permafrost in Bonanza Creek, Alaska, sample from Permafrost Layer P1 (MG-P1-I) (MER-FS) (*)[D]

Soil microbial communities from permafrost in Bona ... za Creek, Alaska, sample from Permafrost Layer P1 (MG-P1-II) (MER-FS) (*)[D]

Soil microbial communities from permafrost in Bona ... a Creek, Alaska, sample from Permafrost Layer P1 (P1_CLC_pe) (MER-FS) (*)[D]

Soil microbial communities from permafrost in Bonanza Creek, Alaska, sample from Permafrost Layer P3 (MG-P3-I) (MER-FS) (*)[D]

Soil microbial communities from permafrost in Bona ... za Creek, Alaska, sample from Permafrost Layer P3 (MG-P3-II) (MER-FS) (*)[D]

Soil microbial communities from permafrost in Bonanza Creek, Alaska, sample from Permafrost Layer P3 (MT-P3-I) (MER-FS) (*)[D]

Soil microbial communities from permafrost in Bona ... Alaska, sample from Permafrost Layer P3 (P3 first assembly) (MER-FS) (*)[D]

Soil microbial communities from permafrost in Bonanza Creek, Alaska, sample from Permafrost Layer P3 (P3_CLC) (MER-FS) (*)[D]

Thermokarst Bog cDNA B3 (MER-FS) (*)[D]

Thermokarst Bog cDNA B3 (MER-FS) (*)[D]

Thermokarst Bog cDNA B4 (MER-FS) (*)[D]

Thermokarst Bog cDNA B4 (MER-FS) (*)[D]

Thermokarst Bog cDNA B4 (MER-FS) (*)[D]

Soil:Unclassified

Microbial communities from Arabidopsis rhizosphere

Arabidopsis rhizosphere microbial communities from ... idopsis soil young (Arabidopsis soil young, Nov 2011 assem) (MER-FS) (*)[D]

Switchgrass rhizosphere microbial community from Michigan, US

Switchgrass rhizosphere microbial community from Michigan, U ... rom Rose Lake RL3 (Rhizo RL3 January 2011 combined assembly) (*)[D]

Switchgrass rhizosphere microbial community from M ... ple from Rose Lake RL3 (Rhizosphere RL3 April 2011 assembly) (MER-FS) (*)[D]

Shrubland

Blue grama grass rhizosphere eukaryotic and microbial communities from Sevilleta Long Term Ecological Research site, New Mexico, US

Blue Grama RHZ soil Unburned sample replicate 2 (MER-FS) (*)[D]

Tropical rainforest

Soil microbial communities from Puerto Rico rain forest

Luquillo Experimental Forest Soil, Puerto Rico (MER-FS) (*)[D]

Soil microbial communities from Puerto Rico rain forest, that decompose switchgrass

Soil microbial communities from Puerto Rico rain f ... s, sample from Feedstock-adapted consortia SG + Fe (SG + Fe) (MER-FS) (*)[D]

Soil microbial communities from Puerto Rico rain f ... stock-adapted consortia SG + Fe (SG + Fe, May 2011 assembly) (MER-FS) (*)[D]

Soil microbial communities from Puerto Rico rain f ... s, sample from feedstock-adapted consortia SG only (SG only) (MER-FS) (*)[D]

Soil microbial communities from Puerto Rico rain f ... stock-adapted consortia SG only (SG only, May 2011 assembly) (MER-FS) (*)[D]

Agricultural land

Soil microbial communities from switchgrass rhizosphere

Maize field bulk soil microbial communities from ... na, IL (Bulk soil sample from field growing corn (Zea mays)) (MER-FS) (*)[F]

Miscanthus field bulk soil microbial communities f ... (Bulk soil sample from field growing Miscanthus x giganteus) (MER-FS) (*)[F]

Switchgrass field bulk soil microbial communities ... il sample from field growing switchgrass (Panicum virgatum)) (MER-FS) (*)[F]

Forest soil microbial communities from Douglas fir zone in British Columbia and Gulf Coastal Plain in Texas

Mediterranean Blodgett CA OM2_M1 (Mediterranean Blodgett CA OM2_M1, ASSEMBLY_DATE=20121207) (MER-FS) (*)[D]

Mediterranean Blodgett CA OM2_M1 (Mediterranean Blodgett CA OM2_M1, July 2012 Assem) (MER-FS) (*)[D]

Mediterranean Blodgett CA OM2_O2 (Mediterranean Blodgett CA OM2_O2, ASSEMBLY_DATE=20121207) (MER-FS) (*)[D]

Mediterranean Blodgett CA OM2_O2 (Mediterranean Blodgett CA OM2_O2, June 2012 Assem) (MER-FS) (*)[D]

Mediterranean Blodgett CA OM3_M2 (Mediterranean Blodgett CA OM3_M2, ASSEMBLY_DATE=20121207) (MER-FS) (*)[D]

Mediterranean Blodgett CA OM3_M2 (Mediterranean Blodgett CA OM3_M2, June 2012 Assem) (MER-FS) (*)[D]

Mediterranean Blodgett CA Ref_O3 (Mediterranean Blodgett CA Ref_O3, ASSEMBLY_DATE=20130115) (MER-FS) (*)[D]

Mediterranean Blodgett CA Ref_O3 (Mediterranean Blodgett CA Ref_O3, June 2012 Assem) (MER-FS) (*)[D]

Uranium contaminated

Terabase sequencing for comprehensive genome reconstruction to assess metabolic potential for environmental bioremediation

Rifle sediment 13ft 3 (Rifle sediment 13ft 3, ASSEMBLY_DATE=20130221) (MER-FS) (*)[D]

Loam

Metagenomic and metatranscriptomic analysis of forest soil communities across North America

Black Spruce, Ontario site 2_A8_OM1_M1 (Black Spruce, Ontario site 2_A8_OM1_M1, ASSEMBLY_DATE=20130219) (MER-FS) (*)[D]

Black Spruce, Ontario site 2_A8_OM1_M2 (Black Spruce, Ontario site 2_A8_OM1_M2, ASSEMBLY_DATE=20130311) (MER-FS) (*)[D]

Black Spruce, Ontario site 2_A8_OM1_M3 (Black Spruce, Ontario site 2_A8_OM1_M3, ASSEMBLY_DATE=20130219) (MER-FS) (*)[D]

Black Spruce, Ontario site 2_A8_OM1_O1 (Black Spruce, Ontario site 2_A8_OM1_O1, ASSEMBLY_DATE=20130219) (MER-FS) (*)[D]

Black Spruce, Ontario site 2_A8_OM1_O2 (Black Spruce, Ontario site 2_A8_OM1_O2, ASSEMBLY_DATE=20130227) (MER-FS) (*)[D]

Black Spruce, Ontario site 2_A8_OM1_O3 (Black Spruce, Ontario site 2_A8_OM1_O3, ASSEMBLY_DATE=20130219) (MER-FS) (*)[D]

Black Spruce, Ontario site 2_A8_OM2_M1 (Black Spruce, Ontario site 2_A8_OM2_M1, ASSEMBLY_DATE=20130306) (MER-FS) (*)[D]

Black Spruce, Ontario site 2_A8_OM2_M2 (Black Spruce, Ontario combined, ASSEMBLY_DATE=20130703) (MER-FS) (*)[D]

Black Spruce, Ontario site 2_A8_OM2_M2 (Black Spruce, Ontario site 2_A8_OM2_M2, ASSEMBLY_DATE=20130226) (MER-FS) (*)[D]
Black Spruce, Ontario site 2_A8_OM2_M3 (Black Spruce, Ontario site 2_A8_OM2_M3, ASSEMBLY_DATE=20130311) (MER-FS) (*)[D]
Black Spruce, Ontario site 2_A8_OM2_O1 (Black Spruce, Ontario site 2_A8_OM2_O1, ASSEMBLY_DATE=20130306) (MER-FS) (*)[D]
Black Spruce, Ontario site 2_A8_OM2_O2 (Black Spruce, Ontario site 2_A8_OM2_O2, ASSEMBLY_DATE=20130306) (MER-FS) (*)[D]
Black Spruce, Ontario site 2_A8_OM2_O3 (Black Spruce, Ontario site 2_A8_OM2_O3, ASSEMBLY_DATE=20130311) (MER-FS) (*)[D]
Black Spruce, Ontario site 2_A8_OM3_M1 (Black Spruce, Ontario site 2_A8_OM3_M1, ASSEMBLY_DATE=20130219) (MER-FS) (*)[D]
Black Spruce, Ontario site 2_A8_OM3_M2 (Black Spruce, Ontario site 2_A8_OM3_M2, ASSEMBLY_DATE=20130226) (MER-FS) (*)[D]
Black Spruce, Ontario site 2_A8_OM3_M3 (Black Spruce, Ontario site 2_A8_OM3_M3, ASSEMBLY_DATE=20130219) (MER-FS) (*)[D]
Black Spruce, Ontario site 2_A8_Ref_M1 (Black Spruce, Ontario site 2_A8_Ref_M1, ASSEMBLY_DATE=20130227) (MER-FS) (*)[D]
Black Spruce, Ontario site 2_A8_Ref_M2 (Black Spruce, Ontario site 2_A8_Ref_M2, ASSEMBLY_DATE=20130227) (MER-FS) (*)[D]
Black Spruce, Ontario site 2_A8_Ref_M3 (Black Spruce, Ontario site 2_A8_Ref_M3, ASSEMBLY_DATE=20130227) (MER-FS) (*)[D]
Black Spruce, Ontario site 2_A8_Ref_O1 (Black Spruce, Ontario site 2_A8_Ref_O1, ASSEMBLY_DATE=20130228) (MER-FS) (*)[D]
Black Spruce, Ontario site 2_A8_Ref_O2 (Black Spruce, Ontario site 2_A8_Ref_O2, ASSEMBLY_DATE=20130227) (MER-FS) (*)[D]
Black Spruce, Ontario site 2_A8_Ref_O3 (Black Spruce, Ontario site 2_A8_Ref_O3, ASSEMBLY_DATE=20130227) (MER-FS) (*)[D]
Jack Pine, Ontario site 1_JW_OM1H0_M1 (Jack Pine, Ontario site 1_JW_OM1H0_M1, ASSEMBLY_DATE=20130226) (MER-FS) (*)[D]
Jack Pine, Ontario site 1_JW_OM1H0_M2 (Jack Pine, Ontario site 1_JW_OM1H0_M2, ASSEMBLY_DATE=20130306) (MER-FS) (*)[D]
Jack Pine, Ontario site 1_JW_OM1H0_M3 (Jack Pine, Ontario site 1_JW_OM1H0_M3, ASSEMBLY_DATE=20130311) (MER-FS) (*)[D]
Jack Pine, Ontario site 1_JW_OM1H0_O1 (Jack Pine, Ontario site 1_JW_OM1H0_O1, ASSEMBLY_DATE=20130226) (MER-FS) (*)[D]
Jack Pine, Ontario site 1_JW_OM1H0_O2 (Jack Pine, Ontario site 1_JW_OM1H0_O2, ASSEMBLY_DATE=20130306) (MER-FS) (*)[D]
Jack Pine, Ontario site 1_JW_OM1H0_O3 (Jack Pine, Ontario site 1_JW_OM1H0_O3, ASSEMBLY_DATE=20130306) (MER-FS) (*)[D]
Jack Pine, Ontario site 1_JW_OM2H0_M1 (Jack Pine, Ontario site 1_JW_OM2H0_M1, ASSEMBLY_DATE=20130616) (MER-FS) (*)[D]
Jack Pine, Ontario site 1_JW_OM2H0_M2 (Jack Pine, Ontario site 1_JW_OM2H0_M2, ASSEMBLY_DATE=20130311) (MER-FS) (*)[D]
Jack Pine, Ontario site 1_JW_OM2H0_M3 (Jack Pine, Ontario site 1_JW_OM2H0_M3, ASSEMBLY_DATE=20130226) (MER-FS) (*)[D]
Jack Pine, Ontario site 1_JW_OM2H0_O1 (Jack Pine, Ontario site 1_JW_OM2H0_O1, ASSEMBLY_DATE=20130605) (MER-FS) (*)[D]
Jack Pine, Ontario site 1_JW_OM2H0_O2 (Jack Pine, Ontario site 1_JW_OM2H0_O2, ASSEMBLY_DATE=20130306) (MER-FS) (*)[D]
Jack Pine, Ontario site 1_JW_OM2H0_O3 (Jack Pine, Ontario site 1_JW_OM2H0_O3, ASSEMBLY_DATE=20130220) (MER-FS) (*)[D]
Jack Pine, Ontario site 1_JW_OM3H0_M1 (Jack Pine, Ontario site 1_JW_OM3H0_M1, ASSEMBLY_DATE=20130501) (MER-FS) (*)[D]
Jack Pine, Ontario site 1_JW_OM3H0_M2 (Jack Pine, Ontario site 1_JW_OM3H0_M2, ASSEMBLY_DATE=20130226) (MER-FS) (*)[D]
Jack Pine, Ontario site 1_JW_OM3H0_M3 (Jack Pine, Ontario site 1_JW_OM3H0_M3, ASSEMBLY_DATE=20130219) (MER-FS) (*)[D]
Jack Pine, Ontario site 1_JW_Ref_M1 (Jack Pine, Ontario site 1_JW_Ref_M1, ASSEMBLY_DATE=20130225) (MER-FS) (*)[D]
Jack Pine, Ontario site 1_JW_Ref_M2 (Jack Pine, Ontario site 1_JW_Ref_M2, ASSEMBLY_DATE=20130311) (MER-FS) (*)[D]
Jack Pine, Ontario site 1_JW_Ref_M3 (Jack Pine, Ontario site 1_JW_Ref_M3, ASSEMBLY_DATE=20130311) (MER-FS) (*)[D]
Jack Pine, Ontario site 1_JW_Ref_O1 (Jack Pine, Ontario site 1_JW_Ref_O1, ASSEMBLY_DATE=20130226) (MER-FS) (*)[D]
Jack Pine, Ontario site 1_JW_Ref_O2 (Jack Pine, Ontario site 1_JW_Ref_O2, ASSEMBLY_DATE=20130606) (MER-FS) (*)[D]
Jack Pine, Ontario site 1_JW_Ref_O3 (Jack Pine, Ontario site 1_JW_Ref_O3, ASSEMBLY_DATE=20130311) (MER-FS) (*)[D]
Texas A ecozone_OM1H0_M1 (Texas A ecozone_OM1H0_M1, ASSEMBLY_DATE=20130221) (MER-FS) (*)[D]
Texas A ecozone_OM1H0_M2 (Texas A ecozone_OM1H0_M2, ASSEMBLY_DATE=20130221) (MER-FS) (*)[D]
Texas A ecozone_OM1H0_M3 (Texas A ecozone_OM1H0_M3, ASSEMBLY_DATE=20130221) (MER-FS) (*)[D]
Texas A ecozone_OM1H0_O1 (Texas A ecozone_OM1H0_O1, ASSEMBLY_DATE=20130221) (MER-FS) (*)[D]
Texas A ecozone_OM1H0_O2 (Texas A ecozone_OM1H0_O2, ASSEMBLY_DATE=20130227) (MER-FS) (*)[D]
Texas A ecozone_OM1H0_O3 (Texas A ecozone_OM1H0_O3, ASSEMBLY_DATE=20130221) (MER-FS) (*)[D]
Texas A ecozone_OM2H0_M1 (Texas A ecozone_OM2H0_M1, ASSEMBLY_DATE=20130227) (MER-FS) (*)[D]
Texas A ecozone_OM2H0_M2 (Texas A ecozone_OM2H0_M2, ASSEMBLY_DATE=20130227) (MER-FS) (*)[D]
Texas A ecozone_OM2H0_O1 (Texas A ecozone_OM2H0_O1, ASSEMBLY_DATE=20130221) (MER-FS) (*)[D]
Texas A ecozone_OM2H0_O2 (Texas A ecozone_OM2H0_O2, ASSEMBLY_DATE=20130227) (MER-FS) (*)[D]
Texas A ecozone_OM2H0_O3 (Texas A ecozone_OM2H0_O3, ASSEMBLY_DATE=20130222) (MER-FS) (*)[D]

Texas A ecozone_OM3H0_M1 (Texas A ecozone_OM3H0_M1, ASSEMBLY_DATE=20130222) (MER-FS) (*)[D]

Texas A ecozone_OM3H0_M2 (Texas A ecozone_OM3H0_M2, ASSEMBLY_DATE=20130221) (MER-FS) (*)[D]

Texas A ecozone_OM3H0_M3 (Texas A ecozone_OM3H0_M3, ASSEMBLY_DATE=20130226) (MER-FS) (*)[D]

Texas A ecozone_OM3H0_O1 (Texas A ecozone_OM3H0_O1, ASSEMBLY_DATE=20130226) (MER-FS) (*)[D]

Cross-site metagenomic analyses to assess the impacts of experimental nitrogen additions on belowground carbon dynamics

Forest Soil microbial communities with Nitrogen NN ... ial communities with Nitrogen NN103, ASSEMBLY_DATE=20130603) (MER-FS) (*)[D]

Forest Soil microbial communities with Nitrogen NN ... ial communities with Nitrogen NN106, ASSEMBLY_DATE=20130409) (MER-FS) (*)[D]

Forest Soil microbial communities with Nitrogen NN ... ial communities with Nitrogen NN109, ASSEMBLY_DATE=20130603) (MER-FS) (*)[D]

Forest Soil microbial communities with Nitrogen NN ... ial communities with Nitrogen NN112, ASSEMBLY_DATE=20130603) (MER-FS) (*)[D]

Forest Soil microbial communities with Nitrogen NN ... ial communities with Nitrogen NN117, ASSEMBLY_DATE=20130220) (MER-FS) (*)[D]

Forest Soil microbial communities with Nitrogen NN ... ial communities with Nitrogen NN120, ASSEMBLY_DATE=20130213) (MER-FS) (*)[D]

Forest Soil microbial communities with Nitrogen NN ... ial communities with Nitrogen NN391, ASSEMBLY_DATE=20130213) (MER-FS) (*)[D]

Forest Soil microbial communities with Nitrogen NN ... ial communities with Nitrogen NN393, ASSEMBLY_DATE=20130213) (MER-FS) (*)[D]

Forest Soil microbial communities with Nitrogen NN ... ial communities with Nitrogen NN395, ASSEMBLY_DATE=20130221) (MER-FS) (*)[D]

Forest Soil microbial communities with Nitrogen NN ... ial communities with Nitrogen NN397, ASSEMBLY_DATE=20130221) (MER-FS) (*)[D]

Forest Soil microbial communities with Nitrogen NN ... ial communities with Nitrogen NN407, ASSEMBLY_DATE=20130221) (MER-FS) (*)[D]

Forest Soil microbial communities with Nitrogen NN ... ial communities with Nitrogen NN410, ASSEMBLY_DATE=20130213) (MER-FS) (*)[D]

Forest Soil microbial communities with Nitrogen NN ... ial communities with Nitrogen NN411, ASSEMBLY_DATE=20130213) (MER-FS) (*)[D]

Forest Soil microbial communities with Nitrogen NN ... ial communities with Nitrogen NN412, ASSEMBLY_DATE=20130213) (MER-FS) (*)[D]

Forest Soil microbial communities with Nitrogen NN ... ial communities with Nitrogen NN413, ASSEMBLY_DATE=20130213) (MER-FS) (*)[D]

Forest Soil microbial communities with Nitrogen NN ... bial communities with Nitrogen NN91, ASSEMBLY_DATE=20130603) (MER-FS) (*)[D]

Forest Soil microbial communities with Nitrogen NN ... bial communities with Nitrogen NN94, ASSEMBLY_DATE=20130603) (MER-FS) (*)[D]

Grasslands

Cross-site metagenomic analyses to assess the impacts of experimental nitrogen additions on belowground carbon dynamics

Forest Soil microbial communities with Nitrogen NN ... al communities with Nitrogen NN1093, ASSEMBLY_DATE=20130213) (MER-FS) (*)[D]

Forest Soil microbial communities with Nitrogen NN ... al communities with Nitrogen NN1094, ASSEMBLY_DATE=20130213) (MER-FS) (*)[D]

Forest Soil microbial communities with Nitrogen NN ... al communities with Nitrogen NN1095, ASSEMBLY_DATE=20130213) (MER-FS) (*)[D]

Forest Soil microbial communities with Nitrogen NN ... al communities with Nitrogen NN1096, ASSEMBLY_DATE=20130213) (MER-FS) (*)[D]

Forest Soil microbial communities with Nitrogen NN ... al communities with Nitrogen NN1097, ASSEMBLY_DATE=20130213) (MER-FS) (*)[D]

Forest Soil microbial communities with Nitrogen NN ... al communities with Nitrogen NN1101, ASSEMBLY_DATE=20130221) (MER-FS) (*)[D]

Forest Soil microbial communities with Nitrogen NN ... al communities with Nitrogen NN1102, ASSEMBLY_DATE=20130213) (MER-FS) (*)[D]

Forest Soil microbial communities with Nitrogen NN ... al communities with Nitrogen NN1104, ASSEMBLY_DATE=20130213) (MER-FS) (*)[D]

Forest Soil microbial communities with Nitrogen NN ... al communities with Nitrogen NN1105, ASSEMBLY_DATE=20130213) (MER-FS) (*)[D]

Forest Soil microbial communities with Nitrogen NN ... al communities with Nitrogen NN1109, ASSEMBLY_DATE=20130213) (MER-FS) (*)[D]

Forest Soil microbial communities with Nitrogen NN ... al communities with Nitrogen NN1111, ASSEMBLY_DATE=20130213) (MER-FS) (*)[D]

Forest Soil microbial communities with Nitrogen NN ... al communities with Nitrogen NN1116, ASSEMBLY_DATE=20130213) (MER-FS) (*)[D]

Forest Soil microbial communities with Nitrogen NN ... al communities with Nitrogen NN1117, ASSEMBLY_DATE=20130213) (MER-FS) (*)[D]

Forest Soil microbial communities with Nitrogen NN ... al communities with Nitrogen NN1118, ASSEMBLY_DATE=20130213) (MER-FS) (*)[D]

Forest Soil microbial communities with Nitrogen NN ... al communities with Nitrogen NN1120, ASSEMBLY_DATE=20130213) (MER-FS) (*)[D]

Forest Soil microbial communities with Nitrogen NN ... ial communities with Nitrogen NN574, ASSEMBLY_DATE=20130213) (MER-FS) (*)[D]

Forest Soil microbial communities with Nitrogen NN ... ial communities with Nitrogen NN575, ASSEMBLY_DATE=20130221) (MER-FS) (*)[D]

Forest Soil microbial communities with Nitrogen NN ... ial communities with Nitrogen NN577, ASSEMBLY_DATE=20130220) (MER-FS) (*)[D]

Forest Soil microbial communities with Nitrogen NN ... ial communities with Nitrogen NN581, ASSEMBLY_DATE=20130220) (MER-FS) (*)[D]

Forest Soil microbial communities with Nitrogen NN ... ial communities with Nitrogen NN588, ASSEMBLY_DATE=20130213) (MER-FS) (*)[D]

Forest Soil microbial communities with Nitrogen NN ... ial communities with Nitrogen NN589, ASSEMBLY_DATE=20130213) (MER-FS) (*)[D]

Forest Soil microbial communities with Nitrogen NN ... ial communities with Nitrogen NN591, ASSEMBLY_DATE=20130221) (MER-FS) (*)[D]

Forest Soil microbial communities with Nitrogen NN ... ial communities with Nitrogen NN592, ASSEMBLY_DATE=20130213) (MER-FS) (*)[D]

Forest Soil microbial communities with Nitrogen NN ... ial communities with Nitrogen NN593, ASSEMBLY_DATE=20130213) (MER-FS) (*)[D]

Forest Soil microbial communities with Nitrogen NN ... ial communities with Nitrogen NN595, ASSEMBLY_DATE=20130213) (MER-FS) (*)[D]

Forest Soil microbial communities with Nitrogen NN ... ial communities with Nitrogen NN603, ASSEMBLY_DATE=20130213) (MER-FS) (*)[D]

Forest Soil microbial communities with Nitrogen NN ... ial communities with Nitrogen NN606, ASSEMBLY_DATE=20130213) (MER-FS) (*)[D]

Forest Soil microbial communities with Nitrogen NN ... ial communities with Nitrogen NN607, ASSEMBLY_DATE=20130213) (MER-FS) (*)[D]

Forest Soil microbial communities with Nitrogen NN ... ial communities with Nitrogen NN609, ASSEMBLY_DATE=20130213) (MER-FS) (*)[D]

Forest Soil microbial communities with Nitrogen NN ... ial communities with Nitrogen NN611, ASSEMBLY_DATE=20130213) (MER-FS) (*)[D]

Forest Soil microbial communities with Nitrogen NN ... ial communities with Nitrogen NN613, ASSEMBLY_DATE=20130213) (MER-FS) (*)[D]

Forest Soil microbial communities with Nitrogen NN ... ial communities with Nitrogen NN619, ASSEMBLY_DATE=20130213) (MER-FS) (*)[D]

Forest Soil microbial communities with Nitrogen NN ... ial communities with Nitrogen NN620, ASSEMBLY_DATE=20130220) (MER-FS) (*)[D]

Forest Soil microbial communities with Nitrogen NN ... ial communities with Nitrogen NN621, ASSEMBLY_DATE=20130213) (MER-FS) (*)[D]

Forest Soil microbial communities with Nitrogen NN ... ial communities with Nitrogen NN624, ASSEMBLY_DATE=20130213) (MER-FS) (*)[D]

Forest Soil microbial communities with Nitrogen NN ... ial communities with Nitrogen NN626, ASSEMBLY_DATE=20130213) (MER-FS) (*)[D]

Forest Soil microbial communities with Nitrogen NN ... ial communities with Nitrogen NN628, ASSEMBLY_DATE=20130213) (MER-FS) (*)[D]

Forest Soil microbial communities with Nitrogen NN ... ial communities with Nitrogen NN630, ASSEMBLY_DATE=20130213) (MER-FS) (*)[D]

Forest Soil microbial communities with Nitrogen NN ... bial communities with Nitrogen NN95, ASSEMBLY_DATE=20130520) (MER-FS) (*)[D]

Soil microbial pyrene-degrading mixed culture

Soil microbial pyrene-degrading mixed culture (*)[D]

Desert

Metagenomic Sequencing for Understanding Microbial Carbon Cycling by Biological Soil Crusts of Arid Lands

Soil Crust Dry out, 3 days (biological replicate A) D5A (MER-FS) (*)[D]

Soil Crust Dry out, 3 days (biological replicate B) D5B (MER-FS) (*)[D]

Soil Crust Dry out, 3 days (biological replicate C) D5C (MER-FS) (*)[D]

Soil Crust Prior wet up (biological replicate A) 0A (MER-FS) (*)[D]

Soil Crust Prior wet up (biological replicate B) 0B (MER-FS) (*)[D]

Soil Crust Prior wet up (biological replicate C) 0C (MER-FS) (*)[D]

Soil microbial communities from FACE and OTC sites

Soil microbial communities from sample at FACE Site 3 Nevada Test Site Creosote CO2+ (MER-FS) (*)[D]

Soil microbial communities from sample at FACE Site 3 Nevada Test Site Creosote CO2- (MER-FS) (*)[D]

Soil microbial communities from sample at FACE Site 4 Nevada Test Site Crust CO2+ (NTS Crust elevated CO2) (MER-FS) (*)[D]

Soil microbial communities from sample at FACE Site 4 Nevada Test Site Crust CO2- (MER-FS) (*)[D]

Soil microbial communities from sample at FACE Site NTS_007 Nevada Test Site (NTS_007) (MER-FS) (*)[D]

Soil microbial communities from sample at FACE Site NTS_067 Nevada Test Site (NTS_067) (MER-FS) (*)[D]

Soil microbial communities from sample at FACE Site NTS_071 Nevada Test Site (NTS_071) (MER-FS) (*)[D]

Soil microbial communities from sample at Multiple FACE and OTC sites (NTS_010) (MER-FS) (*)[D]

Soil microbial communities from four geographically distinct crusts in the Colorado Plateau and Sonoran desert

Soil microbial communities sample from Dark Crust, ... (Dark Crust, Colorado Plateau, Green Butte June 2011 assem) (MER-FS) (*)[D]

Soil microbial communities sample from Dark Crust, Colorado Plateau, Green Butte (Dark crust) (MER-FS) (*)[D]

Soil microbial communities sample from Light Crust ... ght Crust, Colorado Plateau, Green Butte 2 June 2011 assem) (MER-FS) (*)[D]

Soil microbial communities sample from Light Crust ... t, Colorado Plateau, Green Butte 2, Illumina Assem Apr 2012) (MER-FS) (*)[D]

Soil microbial communities sample from Light Crust, Colorado Plateau, Green Butte (Light crust) (MER-FS) (*)[D]

Soil microbial communities sample from Light Crust ... (Light Crust Colorado Plateau Green Butte 2, Oct 2011 assem) (MER-FS) (*)[D]

Bioreactor

Wastewater treatment Type I Accumulibacter from EBPR Bioreactor in Madison WI

Accumulibacter clade IA genome-pooled fosmids (Acc ... clade IA genome-pooled fosmids, Hudson-Alpha Feb 2013 Assem) (MER-FS) (*)[D]

Wastewater treatment Type I Accumulibacter community from EBPR Bioreactor

Candidatus Accumulibacter phosphatis Type I (MER-FS) (*)[D]

Candidatus Accumulibacter phosphatis Type I (09/10 Assembly including illumina data) (MER-FS) (*)[D]

Candidatus Accumulibacter phosphatis Type I (Possible Clade IA Scaffolds (with 454/Sanger/Illumina)) (*)[D]

Candidatus Accumulibacter phosphatis Type I (Possible Clade IA Scaffolds from 0910 Assembly) (*)[D]

Candidatus Accumulibacter phosphatis Type I (Sanger/454/Illimina Metagenome Assembly < 97% CAP2UW1) (MER-FS) (*)[D]

US sludge - combined Sanger 454 assembly

Sludge/Australian, Phrap Assembly (*)[D]

Sludge/US Virion (fgenesb) (*)[D]

Sludge/US, Jazz Assembly (*)[D]

Sludge/US, Phrap Assembly (*)[D]

Activated sludge

Wastewater treatment plant plasmid pool from Switzerland

Activated sludge plasmid pool Morges (MIRA contigs) (MIRA contigs, 5x coverage) (*)[D]

Activated sludge plasmid pool Morges-2007 (PGA) (MER-FS) (*)[D]

Activated sludge plasmid pool Visp-2009 (Newbler) (MER-FS) (*)[D]

Nitrogen removal

Bioreactor Anammox bacterial community from Nijmegen, The Netherlands

Bioreactor Anammox bacterial community from Nijmegen, The Netherlands, sample from Brocadia fulgida enrichment (MER-FS) (*)[D]

Bioreactor Anammox bacterial community from Nijmegen, The Ne ... , sample from Brocadia fulgida enrichment (Brocadia contigs) (*)[D]

Bioreactor Anammox bacterial community from Nijmeg ... n, The Netherlands, sample from Scalindua species enirchment (MER-FS) (*)[D]

Bioreactor Anammox bacterial community from Nijmegen, The Netherlands, sample from Scalindua species enirchment (*)[D]

Biofuel metagenome

Biofuel metagenome 3 (Biofuel metagenome 3 - Enterobacter Contigs, August 2012 ALLpaths Assem) (MER-FS) (*)[D]

Biofuel metagenome 3 (Biofuel metagenome 3 - Enterobacter Scaffolds, August 2012 ALLpaths Assem) (MER-FS) (*)[D]

Biofuel metagenome 3 (Biofuel metagenome 3 Illumina assembly) (MER-FS) (*)[D]

Biofuel metagenome 3 (Biofuel metagenome 3 July 2011 assem) (MER-FS) (*)[D]

Petrochemical

Hydrocarbon Resource Environments Microbial Communities

CG13:San Juan basin coal bed production water (CG13: San Juan Basin coal bed production water 454 assembly) (MER-FS) (*)[D]

CG19:San Juan basin coal bed production water (CG19: San Juan Basin coal bed production water 454 assembly) (MER-FS) (*)[D]

CG7:San Juan basin coal bed production water (CG7: San Juan Basin coal bed production water 454 assembly) (MER-FS) (*)[D]

CG8:San Juan basin coal bed production water (CG8: San Juan Basin coal bed production water 454 assembly) (MER-FS) (*)[D]

Unclassified

Produced water sample 10PW from MHGC oil field

PW_MHGC_2012April10: Produced water sample 10PW from MHGC oil field (PW_MHGC_2012April10: 454 assembly) (MER-FS) (*)[D]

Hydrocarbon Resource Environments Microbial Communities

SyncrudeMLSB2011: syncurde West In-Pit metagenome (454 assembly) (MER-FS) (*)[D]

TP6_2010: Tailings pond microbial communities from Northern Alberta (MER-FS) (*)[D]

WIP-PD_SYN_TP_WS_002_003_071511: WIP surface sample (WIP-PD_SYN_TP_WS_002_003_071511: 454 assembly) (MER-FS) (*)[D]

Biofuel metagenome

Biofuel metagenome 1 (454/Illumina combined assembly) (MER-FS) (*)[D]

Biofuel metagenome 2 (Biofuel metagenome 2 Illumina assembly) (MER-FS) (*)[D]

Environmental microbial communities from Soda lakes and Soda Solonchak soils

Soda Reactor TP_S13sol (Soda Reactor TP_S13sol, Oct 2012 Assem) (MER-FS) (*)[D]

Soda Reactor TP_S2 (Soda Reactor TP_S2, July 2012 Assem) (MER-FS) (*)[D]

Soda Reactor TP_S3 (Soda Reactor TP_S3, July 2012 Assem) (MER-FS) (*)[D]

Hydrocarbon Resource Environments Microbial Communities

Coal cuttings from Coal bed Methane well site (CO182: coal bed cutting Illumina Assembly) (MER-FS) (*)[D]

Coal cuttings from Coal bed Methane well site (CO183: coal bed cutting Illumina Assembly) (MER-FS) (*)[D]

Trident_1560D: Coal Bed microbial communities from Alberta (MER-FS) (*)[D]

Mixed alcohol (MixAlco) bioreactor

Mixed alcohol bioreactor microbial communities from Texas A& ... sample 55C, Day 16 (55 degree reactor, August 2010 assembly) (*)[D]

Bioreactor

Green-waste compost microbial community from soild state bioreactor

Compost Minireactor Metagenome (final assembly) (MER-FS) (*)[D]

Rice-straw enriched compost microbial community from Berkeley

Mesophilic rice straw/compost enrichment metagenome: eDNA_1 (Mesophilic 454 only) (MER-FS) (*)[D]

Mesophilic rice straw/compost enrichment metagenom ... : eDNA_1 (Mesophilic 454/Illumina Combined June 2011 assem) (MER-FS) (*)[D]

Mesophilic rice straw/compost enrichment metagenome: eDNA_1 (Mesophilic March 2011 454/Illumina combined) (MER-FS) (*)[D]

Mesophilic rice straw/compost enrichment metagenome: eDNA_1 (Mesophilic May 2011 454/Illumina combined) (MER-FS) (*)[D]

Thermophilic rice straw/compost enrichment metagenome: eDNA_2 (March 2011 combined assembly) (MER-FS) (*)[D]

Thermophilic rice straw/compost enrichment metagenome: eDNA_2 (Thermophilic 454 only) (MER-FS) (*)[D]

Thermophilic rice straw/compost enrichment metagen ... DNA_2 (Thermophilic 454/Illumina Combined June 2011 assem) (MER-FS) (*)[D]

Thermophilic rice straw/compost enrichment metagenome: eDNA_2 (Thermophilic combined May 2011) (MER-FS) (*)[D]

Thermophilic enrichment culture SG0.5JP960

Thermophilic enrichment culture SG0.5JP960 (454-Illumina assembly) - version 2 (454-Illumina assembly v2) (*)[D]

Thermophilic enrichment culture SG0.5Z960 (454-Illumina assembly) (454-Illumina assembly) (*)[D]

Unclassified

Feedstock adapted compost microbial communities fr ... wbly Island Compost Facility, Passage 4_AFX, Feb 2012 assem) (MER-FS) (*)[D]

Feedstock adapted compost microbial communities fr ... ewbly Island Compost Facility, Passage 4_IL, Feb 2012 assem) (MER-FS) (*)[D]

Feedstock adapted compost microbial communities fr ... wbly Island Compost Facility, Passage 4_MCC, Feb 2012 assem) (MER-FS) (*)[D]

Feedstock adapted compost microbial communities fr ... ewbly Island Compost Facility, Passage 4_SG, Feb 2012 assem) (MER-FS) (*)[D]

Feedstock adapted compost microbial communities fr ... ly Island Compost Facility, Passage 4_Xylan, Feb 2012 assem) (MER-FS) (*)[D]

Sao Paulo Zoo Compost

ZC1 (MER-FS) (*)[D]

ZC2 (MER-FS) (*)[D]

Wood

Poplar biomass decaying microbial community

Poplar biomass bioreactor microbial communities from Brookhaven National Lab, NY, sample from pooled GH fosmids (*)[D]

Poplar biomass bioreactor microbial communities from Brookha ... National Lab, NY, sample from total biomass decay community (*)[D]

Poplar biomass bioreactor microbial communities fr ... National Lab, NY, sample from total biomass decay community (MER-FS) (*)[D]

Poplar biomass bioreactor microbial communities from Brookha ... National Lab, NY, sample from total biomass decay community (*)[D]

Poplar biomass bioreactor microbial communities from Brookha ... decay community (13 April 2010 assembly with 454 paired-end) (*)[D]

Poplar biomass bioreactor microbial communities fr ... y community (Untrimmed scaffolds, contigs, trimmed singlets) (MER-FS) (*)[D]

Decomposing wood compost microbial communities from rain forest habitat in Puerto Rico, that are thermophilic

Thermal compost enrichment from Puerto Rico rainfo ... t (Compost thermophiles - Biomass metagenome May 2011 assem) (MER-FS) (*)[D]

Bioremediation

Contaminated groundwater dechlorinating community (KB-1) from synthetic mineral medium in Toronto, ON

Groundwater dechlorinating community (KB-1) from s ... , ON, sample from Site contaminated with chlorinated ethenes (MER-FS) (*)[D]

Soil microbial community from Bioreactor with Chloroethene contaminated sediment

Soil microbial community from bioreactor at Alamed ... Air Station, CA, contaminated with Chloroethene, Sample 196 (MER-FS) (*)[D]

Soil microbial community from bioreactor at Alamed ... contaminated with Chloroethene, Sample 196 (Jan 2009 assem) (MER-FS) (*)[D]

Groundwater dechlorinating microbial community from Kitchener, Ontario, containing dehalobacter

TCA/MEAL culture (TCA/MEAL culture Nov 2010 assembly with PE data) (MER-FS) (*)[D]

TCA/MEAL culture (TCA/MEAL culture) (MER-FS) (*)[D]

Bioreactor

PCE-dechlorinating microbial communities from Ithaca, NY

PCE-dechlorinating microbial communities from Ithaca, NY (Untrimmed contigs) (Untrimmed contigs) (*)[D]

PCE-dechlorinating mixed culture (PCEOT) from Ithaca, NY (MER-FS) (*)[D]

Bioremediation:Unclassified

Hypersaline water microbial communities from Lyngbya mats, Guerrero Negro, Mexico and Elkhorn Slough mats, California, USA

Hot Lake Anabaena-heterotroph coculture metagenome (Hot Lake Anabaena-heterotroph coculture, July 2012 Assem) (MER-FS) (*)[D]

Hot Lake Oscillatoria-heterotroph coculture metage ... ot Lake Oscillatoria-heterotroph coculture, Sept 2012 Assem) (MER-FS) (*)[D]

Hydrocarbon

Benzene-Degrading Methanogenic communities from Bioreactor

September 2009 gDNA_4 (MER-FS) (*)[D]

September 2009 gDNA_4 (Assembly with PE data) (MER-FS) (*)[D]

September 2009 gDNA_4 (Metafolded sample) (*)[D]

Wastewater

Wastewater Terephthalate-degrading communities from Bioreactor

TA reactor DNA contigs from 4 sample (Terephthalate degrading reactor metagenome contigs from 4 samples) (MER-FS) (*)[F]

Wastewater Terephthalate-degrading communities from Bioreactor (*)[F]

Wastewater:Unclassified

GEBA uncultured microbes

Terephthalate degrading community TA Biofilm (Terephthalate degrading community TA Biofilm, May 2012 Assem) (MER-FS) (*)[D]

Terephthalate degrading community TA Sludge (Terephthalate degrading community TA Sludge, Aug 2012 Assem) (MER-FS) (*)[D]

Persistent organic pollutants (POP)

Hydrocarbon Resource Environments Microbial Communities

TP6_2008_30ft: Tailings pond microbial communities from Northern Alberta (MER-FS) (*)[D]

Unclassified

Microbial community impact on carbon sequestration in managed wetland ?carbon farming?

Wetland Surface Sediment Aug2011 Site C1 Bulk Meta ... ent Aug2011 Site C1 Bulk Metagenome, ASSEMBLY_DATE=20130530) (MER-FS) (*)[D]

Oral TM7 microbial communities of Human

Single-cell genome from subgingival tooth surface TM7a (*)[D]

Single-cell genome from subgingival tooth surface TM7b (*)[D]

Single-cell genome from subgingival tooth surface TM7c (*)[D]

Fecal microbiome of Human from distal gut of healthy adults

Human Gut Community Subject 7 (*)[D]

Human Gut Community Subject 8 (*)[D]

HUMAN MICROBIOME PROJECT (HMP) SAMPLES FROM DIFFERENT BODY SITES

Human Microbiome Production Phase community

Human Anterior nares microbiome from visit number 1 of subject 158337416 (MER-FS) (*)[D]

Human Anterior nares microbiome from visit number 1 of subject 158398106 (MER-FS) (*)[D]

Human Anterior nares microbiome from visit number 1 of subject 158458797 (MER-FS) (*)[D]

Human Anterior nares microbiome from visit number 1 of subject 158479027 (MER-FS) (*)[D]

Human Anterior nares microbiome from visit number 1 of subject 158742018 (MER-FS) (*)[D]

Human Anterior nares microbiome from visit number 1 of subject 158984779 (MER-FS) (*)[D]

Human Anterior nares microbiome from visit number 1 of subject 159207311 (MER-FS) (*)[D]

Human Anterior nares microbiome from visit number 1 of subject 159490532 (MER-FS) (*)[D]

Human Anterior nares microbiome from visit number 1 of subject 159551223 (MER-FS) (*)[D]

Human Anterior nares microbiome from visit number 1 of subject 159571453 (MER-FS) (*)[D]

Human Anterior nares microbiome from visit number 1 of subject 159591683 (MER-FS) (*)[D]

Human Anterior nares microbiome from visit number 1 of subject 159672603 (MER-FS) (*)[D]

Human Anterior nares microbiome from visit number 1 of subject 159753524 (MER-FS) (*)[D]

Human Anterior nares microbiome from visit number 1 of subject 159753524 replicate 1 (MER-FS) (*)[D]

Human Anterior nares microbiome from visit number 1 of subject 159814214 (MER-FS) (*)[D]

Human Anterior nares microbiome from visit number 1 of subject 159915365 (MER-FS) (*)[D]

Human Anterior nares microbiome from visit number 1 of subject 160178356 (MER-FS) (*)[D]

Human Anterior nares microbiome from visit number 1 of subject 160218816 (MER-FS) (*)[D]

Human Anterior nares microbiome from visit number 1 of subject 160319967 (MER-FS) (*)[D]

Human Anterior nares microbiome from visit number 1 of subject 160380657 (MER-FS) (*)[D]

Human Anterior nares microbiome from visit number 1 of subject 160400887 (MER-FS) (*)[D]

Human Anterior nares microbiome from visit number 1 of subject 160421117 (MER-FS) (*)[D]

Human Anterior nares microbiome from visit number 1 of subject 160582958 (MER-FS) (*)[D]

Human Anterior nares microbiome from visit number 1 of subject 160603188 (MER-FS) (*)[D]

Human Anterior nares microbiome from visit number 1 of subject 160643649 (MER-FS) (*)[D]

Human Anterior nares microbiome from visit number 1 of subject 160704339 (MER-FS) (*)[D]

Human Anterior nares microbiome from visit number 1 of subject 160765029 (MER-FS) (*)[D]

Human Anterior nares microbiome from visit number 1 of subject 338793263 (MER-FS) (*)[D]

Human Anterior nares microbiome from visit number 1 of subject 404239096 (MER-FS) (*)[D]

Human Anterior nares microbiome from visit number 1 of subject 508703490 (MER-FS) (*)[D]

Human Anterior nares microbiome from visit number 1 of subject 550534656 (MER-FS) (*)[D]

Human Anterior nares microbiome from visit number 1 of subject 706846339 (MER-FS) (*)[D]

Human Anterior nares microbiome from visit number 1 of subject 737052003 (MER-FS) (*)[D]

Human Anterior nares microbiome from visit number 1 of subject 763435843 (MER-FS) (*)[D]

Human Anterior nares microbiome from visit number 1 of subject 763496533 (MER-FS) (*)[D]

Human Anterior nares microbiome from visit number 1 of subject 763577454 (MER-FS) (*)[D]

Human Anterior nares microbiome from visit number 1 of subject 763860675 (MER-FS) (*)[D]

Human Anterior nares microbiome from visit number 1 of subject 763901136 (MER-FS) (*)[D]

Human Anterior nares microbiome from visit number 1 of subject 763961826 (MER-FS) (*)[D]

Human Anterior nares microbiome from visit number 1 of subject 763982056 (MER-FS) (*)[D]

Human Anterior nares microbiome from visit number 1 of subject 764083206 replicate 1 (MER-FS) (*)[D]

Human Anterior nares microbiome from visit number 1 of subject 764083206 replicate 2 (MER-FS) (*)[D]

Human Anterior nares microbiome from visit number 1 of subject 764224817 (MER-FS) (*)[D]

Human Anterior nares microbiome from visit number 1 of subject 764325968 (MER-FS) (*)[D]

Human Anterior nares microbiome from visit number 1 of subject 764447348 (MER-FS) (*)[D]

Human Anterior nares microbiome from visit number 1 of subject 764487809 (MER-FS) (*)[D]

Human Anterior nares microbiome from visit number 1 of subject 764508039 (MER-FS) (*)[D]

Human Anterior nares microbiome from visit number 1 of subject 764588959 (MER-FS) (*)[D]

Human Anterior nares microbiome from visit number 1 of subject 764649650 (MER-FS) (*)[D]

Human Anterior nares microbiome from visit number 1 of subject 765013792 (MER-FS) (*)[D]

Human Anterior nares microbiome from visit number 1 of subject 765034022 (MER-FS) (*)[D]

Human Anterior nares microbiome from visit number 1 of subject 765074482 (MER-FS) (*)[D]

Human Anterior nares microbiome from visit number 1 of subject 765094712 (MER-FS) (*)[D]

Human Anterior nares microbiome from visit number 1 of subject 765135172 (MER-FS) (*)[D]

Human Anterior nares microbiome from visit number 1 of subject 765337473 (MER-FS) (*)[D]

Human Anterior nares microbiome from visit number 1 of subject 765560005 (MER-FS) (*)[D]

Human Anterior nares microbiome from visit number 1 of subject 765620695 (MER-FS) (*)[D]

Human Anterior nares microbiome from visit number 1 of subject 765640925 (MER-FS) (*)[D]

Human Anterior nares microbiome from visit number 1 of subject 765701615 (MER-FS) (*)[D]

Human Anterior nares microbiome from visit number 1 of subject 823052294 (MER-FS) (*)[D]

Human Anterior nares microbiome from visit number 1 of subject 861967750 (MER-FS) (*)[D]

Human Anterior nares microbiome from visit number 2 of subject 158256496 (MER-FS) (*)[D]

Human Anterior nares microbiome from visit number 2 of subject 158337416 (MER-FS) (*)[D]

Human Anterior nares microbiome from visit number 2 of subject 158479027 (MER-FS) (*)[D]

Human Anterior nares microbiome from visit number 2 of subject 158499257 (MER-FS) (*)[D]

Human Anterior nares microbiome from visit number 2 of subject 158883629 (MER-FS) (*)[D]

Human Anterior nares microbiome from visit number 2 of subject 158924089 (MER-FS) (*)[D]

Human Anterior nares microbiome from visit number 2 of subject 158944319 (MER-FS) (*)[D]

Human Anterior nares microbiome from visit number 2 of subject 159247771 (MER-FS) (*)[D]

Human Anterior nares microbiome from visit number 2 of subject 159369152 (MER-FS) (*)[D]

Human Anterior nares microbiome from visit number 2 of subject 159490532 (MER-FS) (*)[D]

Human Anterior nares microbiome from visit number 2 of subject 159591683 (MER-FS) (*)[D]

Human Anterior nares microbiome from visit number 2 of subject 159713063 (MER-FS) (*)[D]

Human Anterior nares microbiome from visit number 2 of subject 159814214 (MER-FS) (*)[D]

Human Anterior nares microbiome from visit number 2 of subject 763435843 (MER-FS) (*)[D]

Human Anterior nares microbiome from visit number 2 of subject 763496533 (MER-FS) (*)[D]

Human Anterior nares microbiome from visit number 2 of subject 763759525 (MER-FS) (*)[D]

Human Anterior nares microbiome from visit number 2 of subject 763840445 (MER-FS) (*)[D]

Human Anterior nares microbiome from visit number 2 of subject 763961826 replicate 2 (MER-FS) (*)[D]

Human Anterior nares microbiome from visit number 2 of subject 763961826 replicate1 (MER-FS) (*)[D]

Human Anterior nares microbiome from visit number 2 of subject 763982056 (MER-FS) (*)[D]

Human Anterior nares microbiome from visit number 2 of subject 764042746 (MER-FS) (*)[D]

Human Anterior nares microbiome from visit number 2 of subject 764062976 (MER-FS) (*)[D]

Human Anterior nares microbiome from visit number 2 of subject 764083206 replicate 1 (MER-FS) (*)[D]

Human Anterior nares microbiome from visit number 2 of subject 764083206 replicate 2 (MER-FS) (*)[D]
Human Anterior nares microbiome from visit number 2 of subject 764143897 (MER-FS) (*)[D]
Human Anterior nares microbiome from visit number 2 of subject 764305738 (MER-FS) (*)[D]
Human Anterior nares microbiome from visit number 2 of subject 764325968 (MER-FS) (*)[D]
Human Anterior nares microbiome from visit number 2 of subject 764447348 (MER-FS) (*)[D]
Human Anterior nares microbiome from visit number 2 of subject 764487809 (MER-FS) (*)[D]
Human Anterior nares microbiome from visit number 2 of subject 764892411 (MER-FS) (*)[D]
Human Anterior nares microbiome from visit number 2 of subject 765094712 (MER-FS) (*)[D]
Human Anterior nares microbiome from visit number 2 of subject 765560005 (MER-FS) (*)[D]
Human Anterior nares microbiome from visit number 3 of subject 159510762 (MER-FS) (*)[D]

Human Microbiome Production Phase community
Human Attached/Keratinized gingiva microbiome from visit number 1 of subject 763496533 (MER-FS) (*)[D]
Human Attached/Keratinized gingiva microbiome from visit number 1 of subject 763577454 (MER-FS) (*)[D]
Human Attached/Keratinized gingiva microbiome from visit number 1 of subject 763961826 (MER-FS) (*)[D]
Human Attached/Keratinized gingiva microbiome from visit number 2 of subject 763496533 (MER-FS) (*)[D]
Human Attached/Keratinized gingiva microbiome from visit number 2 of subject 763577454 (MER-FS) (*)[D]
Human Attached/Keratinized gingiva microbiome from visit number 2 of subject 763961826 (MER-FS) (*)[D]

Human Microbiome Production Phase community
Human Buccal mucosa microbiome from visit number 1 of subject 158337416 (MER-FS) (*)[D]
Human Buccal mucosa microbiome from visit number 1 of subject 158458797 (MER-FS) (*)[D]
Human Buccal mucosa microbiome from visit number 1 of subject 158499257 (MER-FS) (*)[D]
Human Buccal mucosa microbiome from visit number 1 of subject 158742018 (MER-FS) (*)[D]
Human Buccal mucosa microbiome from visit number 1 of subject 158944319 (MER-FS) (*)[D]
Human Buccal mucosa microbiome from visit number 1 of subject 159207311 (MER-FS) (*)[D]
Human Buccal mucosa microbiome from visit number 1 of subject 159268001 (MER-FS) (*)[D]
Human Buccal mucosa microbiome from visit number 1 of subject 159490532 (MER-FS) (*)[D]
Human Buccal mucosa microbiome from visit number 1 of subject 159551223 (MER-FS) (*)[D]
Human Buccal mucosa microbiome from visit number 1 of subject 159571453 (MER-FS) (*)[D]
Human Buccal mucosa microbiome from visit number 1 of subject 159591683 (MER-FS) (*)[D]
Human Buccal mucosa microbiome from visit number 1 of subject 159611913 (MER-FS) (*)[D]
Human Buccal mucosa microbiome from visit number 1 of subject 159632143 (MER-FS) (*)[D]
Human Buccal mucosa microbiome from visit number 1 of subject 159753524 replicate 1 (MER-FS) (*)[D]
Human Buccal mucosa microbiome from visit number 1 of subject 159753524 replicate 2 (MER-FS) (*)[D]
Human Buccal mucosa microbiome from visit number 1 of subject 159814214 (MER-FS) (*)[D]
Human Buccal mucosa microbiome from visit number 1 of subject 159915365 (MER-FS) (*)[D]
Human Buccal mucosa microbiome from visit number 1 of subject 160158126 (MER-FS) (*)[D]
Human Buccal mucosa microbiome from visit number 1 of subject 160178356 (MER-FS) (*)[D]
Human Buccal mucosa microbiome from visit number 1 of subject 160218816 (MER-FS) (*)[D]
Human Buccal mucosa microbiome from visit number 1 of subject 160319967 (MER-FS) (*)[D]
Human Buccal mucosa microbiome from visit number 1 of subject 160380657 (MER-FS) (*)[D]
Human Buccal mucosa microbiome from visit number 1 of subject 160400887 (MER-FS) (*)[D]
Human Buccal mucosa microbiome from visit number 1 of subject 160502038 (MER-FS) (*)[D]
Human Buccal mucosa microbiome from visit number 1 of subject 160582958 (MER-FS) (*)[D]
Human Buccal mucosa microbiome from visit number 1 of subject 160603188 (MER-FS) (*)[D]

Human Buccal mucosa microbiome from visit number 1 of subject 160643649 (MER-FS) (*)[D]
Human Buccal mucosa microbiome from visit number 1 of subject 160704339 (MER-FS) (*)[D]
Human Buccal mucosa microbiome from visit number 1 of subject 160765029 (MER-FS) (*)[D]
Human Buccal mucosa microbiome from visit number 1 of subject 246515023 (MER-FS) (*)[D]
Human Buccal mucosa microbiome from visit number 1 of subject 338793263 (MER-FS) (*)[D]
Human Buccal mucosa microbiome from visit number 1 of subject 370425937 (MER-FS) (*)[D]
Human Buccal mucosa microbiome from visit number 1 of subject 404239096 (MER-FS) (*)[D]
Human Buccal mucosa microbiome from visit number 1 of subject 508703490 (MER-FS) (*)[D]
Human Buccal mucosa microbiome from visit number 1 of subject 550534656 (MER-FS) (*)[D]
Human Buccal mucosa microbiome from visit number 1 of subject 604812005 (MER-FS) (*)[D]
Human Buccal mucosa microbiome from visit number 1 of subject 638754422 (MER-FS) (*)[D]
Human Buccal mucosa microbiome from visit number 1 of subject 675950834 (MER-FS) (*)[D]
Human Buccal mucosa microbiome from visit number 1 of subject 686765762 (MER-FS) (*)[D]
Human Buccal mucosa microbiome from visit number 1 of subject 706846339 (MER-FS) (*)[D]
Human Buccal mucosa microbiome from visit number 1 of subject 737052003 (MER-FS) (*)[D]
Human Buccal mucosa microbiome from visit number 1 of subject 763435843 (MER-FS) (*)[D]
Human Buccal mucosa microbiome from visit number 1 of subject 763496533 (MER-FS) (*)[D]
Human Buccal mucosa microbiome from visit number 1 of subject 763577454 (MER-FS) (*)[D]
Human Buccal mucosa microbiome from visit number 1 of subject 763759525 (MER-FS) (*)[D]
Human Buccal mucosa microbiome from visit number 1 of subject 763820215 (MER-FS) (*)[D]
Human Buccal mucosa microbiome from visit number 1 of subject 763840445 (MER-FS) (*)[D]
Human Buccal mucosa microbiome from visit number 1 of subject 763860675 (MER-FS) (*)[D]
Human Buccal mucosa microbiome from visit number 1 of subject 763901136 (MER-FS) (*)[D]
Human Buccal mucosa microbiome from visit number 1 of subject 763961826 (MER-FS) (*)[D]
Human Buccal mucosa microbiome from visit number 1 of subject 763982056 (MER-FS) (*)[D]
Human Buccal mucosa microbiome from visit number 1 of subject 764042746 (MER-FS) (*)[D]
Human Buccal mucosa microbiome from visit number 1 of subject 764083206 (MER-FS) (*)[D]
Human Buccal mucosa microbiome from visit number 1 of subject 764224817 (MER-FS) (*)[D]
Human Buccal mucosa microbiome from visit number 1 of subject 764285508 (MER-FS) (*)[D]
Human Buccal mucosa microbiome from visit number 1 of subject 764305738 replicate 1 (MER-FS) (*)[D]
Human Buccal mucosa microbiome from visit number 1 of subject 764305738 replicate 2 (MER-FS) (*)[D]
Human Buccal mucosa microbiome from visit number 1 of subject 764447348 (MER-FS) (*)[D]
Human Buccal mucosa microbiome from visit number 1 of subject 764487809 (MER-FS) (*)[D]
Human Buccal mucosa microbiome from visit number 1 of subject 764508039 (MER-FS) (*)[D]
Human Buccal mucosa microbiome from visit number 1 of subject 764588959 (MER-FS) (*)[D]
Human Buccal mucosa microbiome from visit number 1 of subject 764649650 (MER-FS) (*)[D]
Human Buccal mucosa microbiome from visit number 1 of subject 765013792 (MER-FS) (*)[D]
Human Buccal mucosa microbiome from visit number 1 of subject 765034022 (MER-FS) (*)[D]
Human Buccal mucosa microbiome from visit number 1 of subject 765074482 (MER-FS) (*)[D]
Human Buccal mucosa microbiome from visit number 1 of subject 765094712 (MER-FS) (*)[D]
Human Buccal mucosa microbiome from visit number 1 of subject 765135172 (MER-FS) (*)[D]
Human Buccal mucosa microbiome from visit number 1 of subject 765337473 (MER-FS) (*)[D]
Human Buccal mucosa microbiome from visit number 1 of subject 765560005 (MER-FS) (*)[D]
Human Buccal mucosa microbiome from visit number 1 of subject 765620695 (MER-FS) (*)[D]
Human Buccal mucosa microbiome from visit number 1 of subject 765701615 (MER-FS) (*)[D]
Human Buccal mucosa microbiome from visit number 1 of subject 809635352 (MER-FS) (*)[D]

Human Buccal mucosa microbiome from visit number 1 of subject 823052294 (MER-FS) (*)[D]
Human Buccal mucosa microbiome from visit number 1 of subject 861967750 (MER-FS) (*)[D]
Human Buccal mucosa microbiome from visit number 2 of subject 158256496 (MER-FS) (*)[D]
Human Buccal mucosa microbiome from visit number 2 of subject 158337416 (MER-FS) (*)[D]
Human Buccal mucosa microbiome from visit number 2 of subject 158479027 (MER-FS) (*)[D]
Human Buccal mucosa microbiome from visit number 2 of subject 158499257 (MER-FS) (*)[D]
Human Buccal mucosa microbiome from visit number 2 of subject 158802708 (MER-FS) (*)[D]
Human Buccal mucosa microbiome from visit number 2 of subject 158883629 (MER-FS) (*)[D]
Human Buccal mucosa microbiome from visit number 2 of subject 158944319 (MER-FS) (*)[D]
Human Buccal mucosa microbiome from visit number 2 of subject 159005010 (MER-FS) (*)[D]
Human Buccal mucosa microbiome from visit number 2 of subject 159207311 (MER-FS) (*)[D]
Human Buccal mucosa microbiome from visit number 2 of subject 159247771 (MER-FS) (*)[D]
Human Buccal mucosa microbiome from visit number 2 of subject 159268001 (MER-FS) (*)[D]
Human Buccal mucosa microbiome from visit number 2 of subject 159369152 (MER-FS) (*)[D]
Human Buccal mucosa microbiome from visit number 2 of subject 159490532 (MER-FS) (*)[D]
Human Buccal mucosa microbiome from visit number 2 of subject 159551223 (MER-FS) (*)[D]
Human Buccal mucosa microbiome from visit number 2 of subject 159571453 (MER-FS) (*)[D]
Human Buccal mucosa microbiome from visit number 2 of subject 159591683 (MER-FS) (*)[D]
Human Buccal mucosa microbiome from visit number 2 of subject 159611913 (MER-FS) (*)[D]
Human Buccal mucosa microbiome from visit number 2 of subject 159713063 (MER-FS) (*)[D]
Human Buccal mucosa microbiome from visit number 2 of subject 159753524 (MER-FS) (*)[D]
Human Buccal mucosa microbiome from visit number 2 of subject 159814214 (MER-FS) (*)[D]
Human Buccal mucosa microbiome from visit number 2 of subject 160158126 (MER-FS) (*)[D]
Human Buccal mucosa microbiome from visit number 2 of subject 246515023 (MER-FS) (*)[D]
Human Buccal mucosa microbiome from visit number 2 of subject 370425937 (MER-FS) (*)[D]
Human Buccal mucosa microbiome from visit number 2 of subject 604812005 (MER-FS) (*)[D]
Human Buccal mucosa microbiome from visit number 2 of subject 638754422 (MER-FS) (*)[D]
Human Buccal mucosa microbiome from visit number 2 of subject 686765762 (MER-FS) (*)[D]
Human Buccal mucosa microbiome from visit number 2 of subject 763435843 (MER-FS) (*)[D]
Human Buccal mucosa microbiome from visit number 2 of subject 763496533 (MER-FS) (*)[D]
Human Buccal mucosa microbiome from visit number 2 of subject 763577454 (MER-FS) (*)[D]
Human Buccal mucosa microbiome from visit number 2 of subject 763759525 (MER-FS) (*)[D]
Human Buccal mucosa microbiome from visit number 2 of subject 763820215 (MER-FS) (*)[D]
Human Buccal mucosa microbiome from visit number 2 of subject 763840445 (MER-FS) (*)[D]
Human Buccal mucosa microbiome from visit number 2 of subject 763901136 (MER-FS) (*)[D]
Human Buccal mucosa microbiome from visit number 2 of subject 763961826 (MER-FS) (*)[D]
Human Buccal mucosa microbiome from visit number 2 of subject 764062976 (MER-FS) (*)[D]
Human Buccal mucosa microbiome from visit number 2 of subject 764083206 (MER-FS) (*)[D]
Human Buccal mucosa microbiome from visit number 2 of subject 764143897 (MER-FS) (*)[D]
Human Buccal mucosa microbiome from visit number 2 of subject 764224817 (MER-FS) (*)[D]
Human Buccal mucosa microbiome from visit number 2 of subject 764305738 (MER-FS) (*)[D]
Human Buccal mucosa microbiome from visit number 2 of subject 764325968 (MER-FS) (*)[D]
Human Buccal mucosa microbiome from visit number 2 of subject 764487809 (MER-FS) (*)[D]
Human Buccal mucosa microbiome from visit number 2 of subject 764811490 (MER-FS) (*)[D]
Human Buccal mucosa microbiome from visit number 2 of subject 764892411 (MER-FS) (*)[D]
Human Buccal mucosa microbiome from visit number 2 of subject 765094712 (MER-FS) (*)[D]

Human Buccal mucosa microbiome from visit number 2 of subject 809635352 (MER-FS) (*)[D]
Human Buccal mucosa microbiome from visit number 3 of subject 158883629 (MER-FS) (*)[D]
Human Buccal mucosa microbiome from visit number 3 of subject 159753524 (MER-FS) (*)[D]
Human Buccal mucosa microbiome from visit number 3 of subject 763536994 (MER-FS) (*)[D]

Human Microbiome Production Phase community
Human Hard palate microbiome from visit number 2 of subject 765560005 (MER-FS) (*)[D]

Human Microbiome Production Phase community
Human Palatine Tonsils microbiome from visit number 1 of subject 763496533 (MER-FS) (*)[D]
Human Palatine Tonsils microbiome from visit number 1 of subject 763577454 (MER-FS) (*)[D]
Human Palatine Tonsils microbiome from visit number 2 of subject 763496533 (MER-FS) (*)[D]
Human Palatine Tonsils microbiome from visit number 2 of subject 763577454 (MER-FS) (*)[D]
Human Palatine Tonsils microbiome from visit number 2 of subject 763961826 (MER-FS) (*)[D]
Human Palatine Tonsils microbiome from visit number 2 of subject 764042746 (MER-FS) (*)[D]

Human Microbiome Production Phase community
Human Saliva microbiome from visit number 1 of subject 763496533 (MER-FS) (*)[D]
Human Saliva microbiome from visit number 1 of subject 763577454 (MER-FS) (*)[D]
Human Saliva microbiome from visit number 1 of subject 763961826 (MER-FS) (*)[D]
Human Saliva microbiome from visit number 2 of subject 763496533 (MER-FS) (*)[D]
Human Saliva microbiome from visit number 2 of subject 763577454 (MER-FS) (*)[D]

Human Microbiome Production Phase community
Human Subgingival plaque microbiome from visit number 1 of subject 763435843 (MER-FS) (*)[D]
Human Subgingival plaque microbiome from visit number 1 of subject 763496533 (MER-FS) (*)[D]
Human Subgingival plaque microbiome from visit number 1 of subject 763577454 (MER-FS) (*)[D]
Human Subgingival plaque microbiome from visit number 1 of subject 763961826 (MER-FS) (*)[D]
Human Subgingival plaque microbiome from visit number 2 of subject 763496533 (MER-FS) (*)[D]
Human Subgingival plaque microbiome from visit number 2 of subject 763577454 (MER-FS) (*)[D]
Human Subgingival plaque microbiome from visit number 2 of subject 763961826 (MER-FS) (*)[D]
Human Subgingival plaque microbiome from visit number 2 of subject 764042746 (MER-FS) (*)[D]

Human Microbiome Production Phase community
Human Supragingival plaque microbiome from visit number 1 of subject 158337416 (MER-FS) (*)[D]
Human Supragingival plaque microbiome from visit number 1 of subject 158458797 (MER-FS) (*)[D]
Human Supragingival plaque microbiome from visit number 1 of subject 158479027 (MER-FS) (*)[D]
Human Supragingival plaque microbiome from visit number 1 of subject 158499257 (MER-FS) (*)[D]
Human Supragingival plaque microbiome from visit number 1 of subject 158742018 (MER-FS) (*)[D]
Human Supragingival plaque microbiome from visit number 1 of subject 158944319 (MER-FS) (*)[D]
Human Supragingival plaque microbiome from visit number 1 of subject 159207311 (MER-FS) (*)[D]
Human Supragingival plaque microbiome from visit number 1 of subject 159227541 (MER-FS) (*)[D]
Human Supragingival plaque microbiome from visit number 1 of subject 159268001 (MER-FS) (*)[D]
Human Supragingival plaque microbiome from visit number 1 of subject 159369152 (MER-FS) (*)[D]
Human Supragingival plaque microbiome from visit number 1 of subject 159490532 (MER-FS) (*)[D]
Human Supragingival plaque microbiome from visit number 1 of subject 159551223 (MER-FS) (*)[D]

Human Supragingival plaque microbiome from visit number 1 of subject 159571453 (MER-FS) (*)[D]
Human Supragingival plaque microbiome from visit number 1 of subject 159591683 (MER-FS) (*)[D]
Human Supragingival plaque microbiome from visit number 1 of subject 159611913 (MER-FS) (*)[D]
Human Supragingival plaque microbiome from visit number 1 of subject 159632143 (MER-FS) (*)[D]
Human Supragingival plaque microbiome from visit number 1 of subject 159753524 (MER-FS) (*)[D]
Human Supragingival plaque microbiome from visit number 1 of subject 159814214 (MER-FS) (*)[D]
Human Supragingival plaque microbiome from visit number 1 of subject 159915365 (MER-FS) (*)[D]
Human Supragingival plaque microbiome from visit number 1 of subject 160158126 (MER-FS) (*)[D]
Human Supragingival plaque microbiome from visit number 1 of subject 160178356 (MER-FS) (*)[D]
Human Supragingival plaque microbiome from visit number 1 of subject 160319967 (MER-FS) (*)[D]
Human Supragingival plaque microbiome from visit number 1 of subject 160380657 (MER-FS) (*)[D]
Human Supragingival plaque microbiome from visit number 1 of subject 160400887 (MER-FS) (*)[D]
Human Supragingival plaque microbiome from visit number 1 of subject 160421117 (MER-FS) (*)[D]
Human Supragingival plaque microbiome from visit number 1 of subject 160502038 (MER-FS) (*)[D]
Human Supragingival plaque microbiome from visit number 1 of subject 160582958 (MER-FS) (*)[D]
Human Supragingival plaque microbiome from visit number 1 of subject 160603188 (MER-FS) (*)[D]
Human Supragingival plaque microbiome from visit number 1 of subject 160643649 (MER-FS) (*)[D]
Human Supragingival plaque microbiome from visit number 1 of subject 160704339 (MER-FS) (*)[D]
Human Supragingival plaque microbiome from visit number 1 of subject 160765029 (MER-FS) (*)[D]
Human Supragingival plaque microbiome from visit number 1 of subject 246515023 (MER-FS) (*)[D]
Human Supragingival plaque microbiome from visit number 1 of subject 338793263 (MER-FS) (*)[D]
Human Supragingival plaque microbiome from visit number 1 of subject 370425937 (MER-FS) (*)[D]
Human Supragingival plaque microbiome from visit number 1 of subject 404239096 (MER-FS) (*)[D]
Human Supragingival plaque microbiome from visit number 1 of subject 508703490 (MER-FS) (*)[D]
Human Supragingival plaque microbiome from visit number 1 of subject 550534656 (MER-FS) (*)[D]
Human Supragingival plaque microbiome from visit number 1 of subject 604812005 (MER-FS) (*)[D]
Human Supragingival plaque microbiome from visit number 1 of subject 638754422 (MER-FS) (*)[D]
Human Supragingival plaque microbiome from visit number 1 of subject 675950834 (MER-FS) (*)[D]
Human Supragingival plaque microbiome from visit number 1 of subject 686765762 (MER-FS) (*)[D]
Human Supragingival plaque microbiome from visit number 1 of subject 706846339 (MER-FS) (*)[D]
Human Supragingival plaque microbiome from visit number 1 of subject 737052003 (MER-FS) (*)[D]
Human Supragingival plaque microbiome from visit number 1 of subject 763496533 (MER-FS) (*)[D]
Human Supragingival plaque microbiome from visit number 1 of subject 763577454 (MER-FS) (*)[D]
Human Supragingival plaque microbiome from visit number 1 of subject 763759525 (MER-FS) (*)[D]
Human Supragingival plaque microbiome from visit number 1 of subject 763840445 (MER-FS) (*)[D]
Human Supragingival plaque microbiome from visit number 1 of subject 763860675 (MER-FS) (*)[D]
Human Supragingival plaque microbiome from visit number 1 of subject 763901136 (MER-FS) (*)[D]
Human Supragingival plaque microbiome from visit number 1 of subject 763961826 (MER-FS) (*)[D]
Human Supragingival plaque microbiome from visit number 1 of subject 763982056 (MER-FS) (*)[D]
Human Supragingival plaque microbiome from visit number 1 of subject 764042746 (MER-FS) (*)[D]
Human Supragingival plaque microbiome from visit number 1 of subject 764083206 replicate 1 (MER-FS) (*)[D]
Human Supragingival plaque microbiome from visit number 1 of subject 764083206 replicate 2 (MER-FS) (*)[D]
Human Supragingival plaque microbiome from visit number 1 of subject 764143897 (MER-FS) (*)[D]
Human Supragingival plaque microbiome from visit number 1 of subject 764224817 (MER-FS) (*)[D]
Human Supragingival plaque microbiome from visit number 1 of subject 764285508 (MER-FS) (*)[D]
Human Supragingival plaque microbiome from visit number 1 of subject 764305738 (MER-FS) (*)[D]

Human Supragingival plaque microbiome from visit number 1 of subject 764325968 (MER-FS) (*)[D]
Human Supragingival plaque microbiome from visit number 1 of subject 764447348 (MER-FS) (*)[D]
Human Supragingival plaque microbiome from visit number 1 of subject 764487809 (MER-FS) (*)[D]
Human Supragingival plaque microbiome from visit number 1 of subject 764508039 (MER-FS) (*)[D]
Human Supragingival plaque microbiome from visit number 1 of subject 764588959 (MER-FS) (*)[D]
Human Supragingival plaque microbiome from visit number 1 of subject 764649650 (MER-FS) (*)[D]
Human Supragingival plaque microbiome from visit number 1 of subject 765013792 (MER-FS) (*)[D]
Human Supragingival plaque microbiome from visit number 1 of subject 765034022 (MER-FS) (*)[D]
Human Supragingival plaque microbiome from visit number 1 of subject 765074482 (MER-FS) (*)[D]
Human Supragingival plaque microbiome from visit number 1 of subject 765094712 (MER-FS) (*)[D]
Human Supragingival plaque microbiome from visit number 1 of subject 765135172 (MER-FS) (*)[D]
Human Supragingival plaque microbiome from visit number 1 of subject 765337473 (MER-FS) (*)[D]
Human Supragingival plaque microbiome from visit number 1 of subject 765560005 (MER-FS) (*)[D]
Human Supragingival plaque microbiome from visit number 1 of subject 765620695 (MER-FS) (*)[D]
Human Supragingival plaque microbiome from visit number 1 of subject 765640925 (MER-FS) (*)[D]
Human Supragingival plaque microbiome from visit number 1 of subject 765701615 (MER-FS) (*)[D]
Human Supragingival plaque microbiome from visit number 1 of subject 809635352 (MER-FS) (*)[D]
Human Supragingival plaque microbiome from visit number 1 of subject 861967750 (MER-FS) (*)[D]
Human Supragingival plaque microbiome from visit number 2 of subject 158256496 (MER-FS) (*)[D]
Human Supragingival plaque microbiome from visit number 2 of subject 158337416 (MER-FS) (*)[D]
Human Supragingival plaque microbiome from visit number 2 of subject 158479027 (MER-FS) (*)[D]
Human Supragingival plaque microbiome from visit number 2 of subject 158499257 (MER-FS) (*)[D]
Human Supragingival plaque microbiome from visit number 2 of subject 158802708 (MER-FS) (*)[D]
Human Supragingival plaque microbiome from visit number 2 of subject 158883629 (MER-FS) (*)[D]
Human Supragingival plaque microbiome from visit number 2 of subject 158944319 (MER-FS) (*)[D]
Human Supragingival plaque microbiome from visit number 2 of subject 159005010 (MER-FS) (*)[D]
Human Supragingival plaque microbiome from visit number 2 of subject 159207311 (MER-FS) (*)[D]
Human Supragingival plaque microbiome from visit number 2 of subject 159247771 (MER-FS) (*)[D]
Human Supragingival plaque microbiome from visit number 2 of subject 159268001 (MER-FS) (*)[D]
Human Supragingival plaque microbiome from visit number 2 of subject 159369152 (MER-FS) (*)[D]
Human Supragingival plaque microbiome from visit number 2 of subject 159490532 (MER-FS) (*)[D]
Human Supragingival plaque microbiome from visit number 2 of subject 159510762 (MER-FS) (*)[D]
Human Supragingival plaque microbiome from visit number 2 of subject 159551223 (MER-FS) (*)[D]
Human Supragingival plaque microbiome from visit number 2 of subject 159571453 (MER-FS) (*)[D]
Human Supragingival plaque microbiome from visit number 2 of subject 159591683 (MER-FS) (*)[D]
Human Supragingival plaque microbiome from visit number 2 of subject 159611913 (MER-FS) (*)[D]
Human Supragingival plaque microbiome from visit number 2 of subject 159713063 (MER-FS) (*)[D]
Human Supragingival plaque microbiome from visit number 2 of subject 159814214 (MER-FS) (*)[D]
Human Supragingival plaque microbiome from visit number 2 of subject 160158126 (MER-FS) (*)[D]
Human Supragingival plaque microbiome from visit number 2 of subject 246515023 (MER-FS) (*)[D]
Human Supragingival plaque microbiome from visit number 2 of subject 370425937 (MER-FS) (*)[D]
Human Supragingival plaque microbiome from visit number 2 of subject 604812005 (MER-FS) (*)[D]
Human Supragingival plaque microbiome from visit number 2 of subject 638754422 (MER-FS) (*)[D]
Human Supragingival plaque microbiome from visit number 2 of subject 686765762 (MER-FS) (*)[D]
Human Supragingival plaque microbiome from visit number 2 of subject 763435843 (MER-FS) (*)[D]
Human Supragingival plaque microbiome from visit number 2 of subject 763496533 (MER-FS) (*)[D]

Human Supragingival plaque microbiome from visit number 2 of subject 763577454 (MER-FS) (*)[D]
Human Supragingival plaque microbiome from visit number 2 of subject 763759525 (MER-FS) (*)[D]
Human Supragingival plaque microbiome from visit number 2 of subject 763820215 (MER-FS) (*)[D]
Human Supragingival plaque microbiome from visit number 2 of subject 763840445 (MER-FS) (*)[D]
Human Supragingival plaque microbiome from visit number 2 of subject 763860675 (MER-FS) (*)[D]
Human Supragingival plaque microbiome from visit number 2 of subject 763901136 (MER-FS) (*)[D]
Human Supragingival plaque microbiome from visit number 2 of subject 763961826 (MER-FS) (*)[D]
Human Supragingival plaque microbiome from visit number 2 of subject 763982056 (MER-FS) (*)[D]
Human Supragingival plaque microbiome from visit number 2 of subject 764042746 (MER-FS) (*)[D]
Human Supragingival plaque microbiome from visit number 2 of subject 764062976 (MER-FS) (*)[D]
Human Supragingival plaque microbiome from visit number 2 of subject 764083206 (MER-FS) (*)[D]
Human Supragingival plaque microbiome from visit number 2 of subject 764143897 (MER-FS) (*)[D]
Human Supragingival plaque microbiome from visit number 2 of subject 764224817 (MER-FS) (*)[D]
Human Supragingival plaque microbiome from visit number 2 of subject 764305738 (MER-FS) (*)[D]
Human Supragingival plaque microbiome from visit number 2 of subject 764325968 (MER-FS) (*)[D]
Human Supragingival plaque microbiome from visit number 2 of subject 764487809 (MER-FS) (*)[D]
Human Supragingival plaque microbiome from visit number 2 of subject 764811490 (MER-FS) (*)[D]
Human Supragingival plaque microbiome from visit number 2 of subject 764892411 (MER-FS) (*)[D]
Human Supragingival plaque microbiome from visit number 2 of subject 765094712 (MER-FS) (*)[D]
Human Supragingival plaque microbiome from visit number 2 of subject 809635352 (MER-FS) (*)[D]
Human Supragingival plaque microbiome from visit number 3 of subject 158883629 (MER-FS) (*)[D]
Human Supragingival plaque microbiome from visit number 3 of subject 159510762 (MER-FS) (*)[D]
Human Supragingival plaque microbiome from visit number 3 of subject 763536994 (MER-FS) (*)[D]

Human Microbiome Production Phase community
Human Throat microbiome from visit number 1 of subject 763496533 (MER-FS) (*)[D]
Human Throat microbiome from visit number 1 of subject 763577454 (MER-FS) (*)[D]
Human Throat microbiome from visit number 1 of subject 763961826 (MER-FS) (*)[D]
Human Throat microbiome from visit number 2 of subject 763496533 (MER-FS) (*)[D]
Human Throat microbiome from visit number 2 of subject 763577454 (MER-FS) (*)[D]
Human Throat microbiome from visit number 2 of subject 763961826 (MER-FS) (*)[D]
Human Throat microbiome from visit number 2 of subject 765560005 (MER-FS) (*)[D]

Human Microbiome Production Phase community
Human Tongue dorsum microbiome from visit number 1 of subject 158337416 (MER-FS) (*)[D]
Human Tongue dorsum microbiome from visit number 1 of subject 158458797 (MER-FS) (*)[D]
Human Tongue dorsum microbiome from visit number 1 of subject 158499257 (MER-FS) (*)[D]
Human Tongue dorsum microbiome from visit number 1 of subject 158742018 (MER-FS) (*)[D]
Human Tongue dorsum microbiome from visit number 1 of subject 158944319 (MER-FS) (*)[D]
Human Tongue dorsum microbiome from visit number 1 of subject 159207311 (MER-FS) (*)[D]
Human Tongue dorsum microbiome from visit number 1 of subject 159268001 (MER-FS) (*)[D]
Human Tongue dorsum microbiome from visit number 1 of subject 159369152 (MER-FS) (*)[D]
Human Tongue dorsum microbiome from visit number 1 of subject 159551223 (MER-FS) (*)[D]
Human Tongue dorsum microbiome from visit number 1 of subject 159571453 (MER-FS) (*)[D]
Human Tongue dorsum microbiome from visit number 1 of subject 159591683 (MER-FS) (*)[D]
Human Tongue dorsum microbiome from visit number 1 of subject 159611913 (MER-FS) (*)[D]

Human Tongue dorsum microbiome from visit number 1 of subject 159632143 (MER-FS) (*)[D]
Human Tongue dorsum microbiome from visit number 1 of subject 159753524 replicate 1 (MER-FS) (*)[D]
Human Tongue dorsum microbiome from visit number 1 of subject 159753524 replicate 2 (MER-FS) (*)[D]
Human Tongue dorsum microbiome from visit number 1 of subject 159814214 (MER-FS) (*)[D]
Human Tongue dorsum microbiome from visit number 1 of subject 159915365 (MER-FS) (*)[D]
Human Tongue dorsum microbiome from visit number 1 of subject 160158126 (MER-FS) (*)[D]
Human Tongue dorsum microbiome from visit number 1 of subject 160178356 (MER-FS) (*)[D]
Human Tongue dorsum microbiome from visit number 1 of subject 160218816 (MER-FS) (*)[D]
Human Tongue dorsum microbiome from visit number 1 of subject 160319967 (MER-FS) (*)[D]
Human Tongue dorsum microbiome from visit number 1 of subject 160380657 (MER-FS) (*)[D]
Human Tongue dorsum microbiome from visit number 1 of subject 160400887 (MER-FS) (*)[D]
Human Tongue dorsum microbiome from visit number 1 of subject 160421117 (MER-FS) (*)[D]
Human Tongue dorsum microbiome from visit number 1 of subject 160502038 (MER-FS) (*)[D]
Human Tongue dorsum microbiome from visit number 1 of subject 160582958 (MER-FS) (*)[D]
Human Tongue dorsum microbiome from visit number 1 of subject 160603188 (MER-FS) (*)[D]
Human Tongue dorsum microbiome from visit number 1 of subject 160643649 (MER-FS) (*)[D]
Human Tongue dorsum microbiome from visit number 1 of subject 160704339 (MER-FS) (*)[D]
Human Tongue dorsum microbiome from visit number 1 of subject 160765029 (MER-FS) (*)[D]
Human Tongue dorsum microbiome from visit number 1 of subject 246515023 (MER-FS) (*)[D]
Human Tongue dorsum microbiome from visit number 1 of subject 338793263 (MER-FS) (*)[D]
Human Tongue dorsum microbiome from visit number 1 of subject 370425937 (MER-FS) (*)[D]
Human Tongue dorsum microbiome from visit number 1 of subject 404239096 (MER-FS) (*)[D]
Human Tongue dorsum microbiome from visit number 1 of subject 508703490 (MER-FS) (*)[D]
Human Tongue dorsum microbiome from visit number 1 of subject 550534656 (MER-FS) (*)[D]
Human Tongue dorsum microbiome from visit number 1 of subject 604812005 (MER-FS) (*)[D]
Human Tongue dorsum microbiome from visit number 1 of subject 638754422 (MER-FS) (*)[D]
Human Tongue dorsum microbiome from visit number 1 of subject 675950834 (MER-FS) (*)[D]
Human Tongue dorsum microbiome from visit number 1 of subject 686765762 (MER-FS) (*)[D]
Human Tongue dorsum microbiome from visit number 1 of subject 706846339 (MER-FS) (*)[D]
Human Tongue dorsum microbiome from visit number 1 of subject 737052003 (MER-FS) (*)[D]
Human Tongue dorsum microbiome from visit number 1 of subject 763435843 (MER-FS) (*)[D]
Human Tongue dorsum microbiome from visit number 1 of subject 763536994 (MER-FS) (*)[D]
Human Tongue dorsum microbiome from visit number 1 of subject 763577454 (MER-FS) (*)[D]
Human Tongue dorsum microbiome from visit number 1 of subject 763820215 (MER-FS) (*)[D]
Human Tongue dorsum microbiome from visit number 1 of subject 763840445 (MER-FS) (*)[D]
Human Tongue dorsum microbiome from visit number 1 of subject 763860675 (MER-FS) (*)[D]
Human Tongue dorsum microbiome from visit number 1 of subject 763901136 replicate 2 (MER-FS) (*)[D]
Human Tongue dorsum microbiome from visit number 1 of subject 763961826 (MER-FS) (*)[D]
Human Tongue dorsum microbiome from visit number 1 of subject 763982056 (MER-FS) (*)[D]
Human Tongue dorsum microbiome from visit number 1 of subject 764042746 (MER-FS) (*)[D]
Human Tongue dorsum microbiome from visit number 1 of subject 764083206 (MER-FS) (*)[D]
Human Tongue dorsum microbiome from visit number 1 of subject 764143897 (MER-FS) (*)[D]
Human Tongue dorsum microbiome from visit number 1 of subject 764224817 (MER-FS) (*)[D]
Human Tongue dorsum microbiome from visit number 1 of subject 764285508 (MER-FS) (*)[D]
Human Tongue dorsum microbiome from visit number 1 of subject 764305738 (MER-FS) (*)[D]
Human Tongue dorsum microbiome from visit number 1 of subject 764325968 (MER-FS) (*)[D]

Human Tongue dorsum microbiome from visit number 1 of subject 764447348 (MER-FS) (*)[D]
Human Tongue dorsum microbiome from visit number 1 of subject 764487809 (MER-FS) (*)[D]
Human Tongue dorsum microbiome from visit number 1 of subject 764508039 (MER-FS) (*)[D]
Human Tongue dorsum microbiome from visit number 1 of subject 764588959 (MER-FS) (*)[D]
Human Tongue dorsum microbiome from visit number 1 of subject 764649650 (MER-FS) (*)[D]
Human Tongue dorsum microbiome from visit number 1 of subject 765013792 (MER-FS) (*)[D]
Human Tongue dorsum microbiome from visit number 1 of subject 765034022 (MER-FS) (*)[D]
Human Tongue dorsum microbiome from visit number 1 of subject 765074482 (MER-FS) (*)[D]
Human Tongue dorsum microbiome from visit number 1 of subject 765094712 (MER-FS) (*)[D]
Human Tongue dorsum microbiome from visit number 1 of subject 765135172 (MER-FS) (*)[D]
Human Tongue dorsum microbiome from visit number 1 of subject 765337473 (MER-FS) (*)[D]
Human Tongue dorsum microbiome from visit number 1 of subject 765560005 (MER-FS) (*)[D]
Human Tongue dorsum microbiome from visit number 1 of subject 765620695 (MER-FS) (*)[D]
Human Tongue dorsum microbiome from visit number 1 of subject 765640925 (MER-FS) (*)[D]
Human Tongue dorsum microbiome from visit number 1 of subject 765701615 (MER-FS) (*)[D]
Human Tongue dorsum microbiome from visit number 1 of subject 809635352 (MER-FS) (*)[D]
Human Tongue dorsum microbiome from visit number 1 of subject 823052294 (MER-FS) (*)[D]
Human Tongue dorsum microbiome from visit number 1 of subject 861967750 (MER-FS) (*)[D]
Human Tongue dorsum microbiome from visit number 2 of subject 158256496 (MER-FS) (*)[D]
Human Tongue dorsum microbiome from visit number 2 of subject 158337416 (MER-FS) (*)[D]
Human Tongue dorsum microbiome from visit number 2 of subject 158479027 (MER-FS) (*)[D]
Human Tongue dorsum microbiome from visit number 2 of subject 158499257 (MER-FS) (*)[D]
Human Tongue dorsum microbiome from visit number 2 of subject 158802708 (MER-FS) (*)[D]
Human Tongue dorsum microbiome from visit number 2 of subject 158883629 (MER-FS) (*)[D]
Human Tongue dorsum microbiome from visit number 2 of subject 158944319 (MER-FS) (*)[D]
Human Tongue dorsum microbiome from visit number 2 of subject 159005010 (MER-FS) (*)[D]
Human Tongue dorsum microbiome from visit number 2 of subject 159207311 (MER-FS) (*)[D]
Human Tongue dorsum microbiome from visit number 2 of subject 159227541 (MER-FS) (*)[D]
Human Tongue dorsum microbiome from visit number 2 of subject 159247771 (MER-FS) (*)[D]
Human Tongue dorsum microbiome from visit number 2 of subject 159268001 (MER-FS) (*)[D]
Human Tongue dorsum microbiome from visit number 2 of subject 159369152 (MER-FS) (*)[D]
Human Tongue dorsum microbiome from visit number 2 of subject 159490532 (MER-FS) (*)[D]
Human Tongue dorsum microbiome from visit number 2 of subject 159510762 (MER-FS) (*)[D]
Human Tongue dorsum microbiome from visit number 2 of subject 159551223 (MER-FS) (*)[D]
Human Tongue dorsum microbiome from visit number 2 of subject 159571453 (MER-FS) (*)[D]
Human Tongue dorsum microbiome from visit number 2 of subject 159591683 (MER-FS) (*)[D]
Human Tongue dorsum microbiome from visit number 2 of subject 159611913 (MER-FS) (*)[D]
Human Tongue dorsum microbiome from visit number 2 of subject 159713063 (MER-FS) (*)[D]
Human Tongue dorsum microbiome from visit number 2 of subject 159814214 (MER-FS) (*)[D]
Human Tongue dorsum microbiome from visit number 2 of subject 160158126 (MER-FS) (*)[D]
Human Tongue dorsum microbiome from visit number 2 of subject 246515023 (MER-FS) (*)[D]
Human Tongue dorsum microbiome from visit number 2 of subject 370425937 (MER-FS) (*)[D]
Human Tongue dorsum microbiome from visit number 2 of subject 604812005 (MER-FS) (*)[D]
Human Tongue dorsum microbiome from visit number 2 of subject 638754422 (MER-FS) (*)[D]
Human Tongue dorsum microbiome from visit number 2 of subject 686765762 (MER-FS) (*)[D]
Human Tongue dorsum microbiome from visit number 2 of subject 763435843 (MER-FS) (*)[D]

Human Tongue dorsum microbiome from visit number 2 of subject 763496533 (MER-FS) (*)[D]
Human Tongue dorsum microbiome from visit number 2 of subject 763536994 (MER-FS) (*)[D]
Human Tongue dorsum microbiome from visit number 2 of subject 763577454 (MER-FS) (*)[D]
Human Tongue dorsum microbiome from visit number 2 of subject 763759525 (MER-FS) (*)[D]
Human Tongue dorsum microbiome from visit number 2 of subject 763820215 (MER-FS) (*)[D]
Human Tongue dorsum microbiome from visit number 2 of subject 763840445 (MER-FS) (*)[D]
Human Tongue dorsum microbiome from visit number 2 of subject 763860675 (MER-FS) (*)[D]
Human Tongue dorsum microbiome from visit number 2 of subject 763901136 replicate 2 (MER-FS) (*)[D]
Human Tongue dorsum microbiome from visit number 2 of subject 763901136 replicate1 (MER-FS) (*)[D]
Human Tongue dorsum microbiome from visit number 2 of subject 763961826 replicate 1 (MER-FS) (*)[D]
Human Tongue dorsum microbiome from visit number 2 of subject 763961826 replicate 2 (MER-FS) (*)[D]
Human Tongue dorsum microbiome from visit number 2 of subject 763982056 (MER-FS) (*)[D]
Human Tongue dorsum microbiome from visit number 2 of subject 764042746 (MER-FS) (*)[D]
Human Tongue dorsum microbiome from visit number 2 of subject 764062976 (MER-FS) (*)[D]
Human Tongue dorsum microbiome from visit number 2 of subject 764083206 (MER-FS) (*)[D]
Human Tongue dorsum microbiome from visit number 2 of subject 764143897 (MER-FS) (*)[D]
Human Tongue dorsum microbiome from visit number 2 of subject 764224817 (MER-FS) (*)[D]
Human Tongue dorsum microbiome from visit number 2 of subject 764305738 (MER-FS) (*)[D]
Human Tongue dorsum microbiome from visit number 2 of subject 764325968 (MER-FS) (*)[D]
Human Tongue dorsum microbiome from visit number 2 of subject 764447348 (MER-FS) (*)[D]
Human Tongue dorsum microbiome from visit number 2 of subject 764487809 (MER-FS) (*)[D]
Human Tongue dorsum microbiome from visit number 2 of subject 764811490 (MER-FS) (*)[D]
Human Tongue dorsum microbiome from visit number 2 of subject 764892411 (MER-FS) (*)[D]
Human Tongue dorsum microbiome from visit number 2 of subject 765094712 (MER-FS) (*)[D]
Human Tongue dorsum microbiome from visit number 2 of subject 809635352 (MER-FS) (*)[D]
Human Tongue dorsum microbiome from visit number 3 of subject 158883629 (MER-FS) (*)[D]
Human Tongue dorsum microbiome from visit number 3 of subject 159510762 (MER-FS) (*)[D]
Human Tongue dorsum microbiome from visit number 3 of subject 159753524 (MER-FS) (*)[D]
Human Tongue dorsum microbiome from visit number 3 of subject 763536994 (MER-FS) (*)[D]

Human Microbiome Production Phase community
Human Stool microbiome from visit number 1 of subject 158337416 (MER-FS) (*)[D]
Human Stool microbiome from visit number 1 of subject 158458797 (MER-FS) (*)[D]
Human Stool microbiome from visit number 1 of subject 158499257 (MER-FS) (*)[D]
Human Stool microbiome from visit number 1 of subject 158742018 (MER-FS) (*)[D]
Human Stool microbiome from visit number 1 of subject 158802708 (MER-FS) (*)[D]
Human Stool microbiome from visit number 1 of subject 158883629 (MER-FS) (*)[D]
Human Stool microbiome from visit number 1 of subject 158944319 (MER-FS) (*)[D]
Human Stool microbiome from visit number 1 of subject 159146620 (MER-FS) (*)[D]
Human Stool microbiome from visit number 1 of subject 159166850 (MER-FS) (*)[D]
Human Stool microbiome from visit number 1 of subject 159207311 (MER-FS) (*)[D]
Human Stool microbiome from visit number 1 of subject 159227541 (MER-FS) (*)[D]
Human Stool microbiome from visit number 1 of subject 159247771 (MER-FS) (*)[D]
Human Stool microbiome from visit number 1 of subject 159268001 (MER-FS) (*)[D]
Human Stool microbiome from visit number 1 of subject 159369152 (MER-FS) (*)[D]
Human Stool microbiome from visit number 1 of subject 159490532 (MER-FS) (*)[D]

Human Stool microbiome from visit number 1 of subject 159510762 (MER-FS) (*)[D]
Human Stool microbiome from visit number 1 of subject 159551223 (MER-FS) (*)[D]
Human Stool microbiome from visit number 1 of subject 159571453 (MER-FS) (*)[D]
Human Stool microbiome from visit number 1 of subject 159591683 (MER-FS) (*)[D]
Human Stool microbiome from visit number 1 of subject 159611913 (MER-FS) (*)[D]
Human Stool microbiome from visit number 1 of subject 159632143 (MER-FS) (*)[D]
Human Stool microbiome from visit number 1 of subject 159733294 (MER-FS) (*)[D]
Human Stool microbiome from visit number 1 of subject 159753524 replicate 1 (MER-FS) (*)[D]
Human Stool microbiome from visit number 1 of subject 159753524 replicate 2 (MER-FS) (*)[D]
Human Stool microbiome from visit number 1 of subject 159814214 (MER-FS) (*)[D]
Human Stool microbiome from visit number 1 of subject 159915365 (MER-FS) (*)[D]
Human Stool microbiome from visit number 1 of subject 160158126 (MER-FS) (*)[D]
Human Stool microbiome from visit number 1 of subject 160178356 (MER-FS) (*)[D]
Human Stool microbiome from visit number 1 of subject 160218816 (MER-FS) (*)[D]
Human Stool microbiome from visit number 1 of subject 160319967 (MER-FS) (*)[D]
Human Stool microbiome from visit number 1 of subject 160380657 (MER-FS) (*)[D]
Human Stool microbiome from visit number 1 of subject 160400887 (MER-FS) (*)[D]
Human Stool microbiome from visit number 1 of subject 160421117 (MER-FS) (*)[D]
Human Stool microbiome from visit number 1 of subject 160502038 (MER-FS) (*)[D]
Human Stool microbiome from visit number 1 of subject 160582958 (MER-FS) (*)[D]
Human Stool microbiome from visit number 1 of subject 160603188 (MER-FS) (*)[D]
Human Stool microbiome from visit number 1 of subject 160643649 (MER-FS) (*)[D]
Human Stool microbiome from visit number 1 of subject 160704339 (MER-FS) (*)[D]
Human Stool microbiome from visit number 1 of subject 160765029 (MER-FS) (*)[D]
Human Stool microbiome from visit number 1 of subject 246515023 (MER-FS) (*)[D]
Human Stool microbiome from visit number 1 of subject 338793263 (MER-FS) (*)[D]
Human Stool microbiome from visit number 1 of subject 370425937 (MER-FS) (*)[D]
Human Stool microbiome from visit number 1 of subject 404239096 (MER-FS) (*)[D]
Human Stool microbiome from visit number 1 of subject 508703490 (MER-FS) (*)[D]
Human Stool microbiome from visit number 1 of subject 550534656 (MER-FS) (*)[D]
Human Stool microbiome from visit number 1 of subject 604812005 (MER-FS) (*)[D]
Human Stool microbiome from visit number 1 of subject 638754422 (MER-FS) (*)[D]
Human Stool microbiome from visit number 1 of subject 675950834 (MER-FS) (*)[D]
Human Stool microbiome from visit number 1 of subject 686765762 (MER-FS) (*)[D]
Human Stool microbiome from visit number 1 of subject 706846339 (MER-FS) (*)[D]
Human Stool microbiome from visit number 1 of subject 737052003 (MER-FS) (*)[D]
Human Stool microbiome from visit number 1 of subject 763496533 (MER-FS) (*)[D]
Human Stool microbiome from visit number 1 of subject 763536994 (MER-FS) (*)[D]
Human Stool microbiome from visit number 1 of subject 763577454 (MER-FS) (*)[D]
Human Stool microbiome from visit number 1 of subject 763678604 (MER-FS) (*)[D]
Human Stool microbiome from visit number 1 of subject 763759525 (MER-FS) (*)[D]
Human Stool microbiome from visit number 1 of subject 763820215 (MER-FS) (*)[D]
Human Stool microbiome from visit number 1 of subject 763840445 (MER-FS) (*)[D]
Human Stool microbiome from visit number 1 of subject 763860675 (MER-FS) (*)[D]
Human Stool microbiome from visit number 1 of subject 763901136 (MER-FS) (*)[D]
Human Stool microbiome from visit number 1 of subject 763961826 (MER-FS) (*)[D]

Human Stool microbiome from visit number 1 of subject 763982056 (MER-FS) (*)[D]
Human Stool microbiome from visit number 1 of subject 764042746 (MER-FS) (*)[D]
Human Stool microbiome from visit number 1 of subject 764062976 (MER-FS) (*)[D]
Human Stool microbiome from visit number 1 of subject 764143897 (MER-FS) (*)[D]
Human Stool microbiome from visit number 1 of subject 764224817 (MER-FS) (*)[D]
Human Stool microbiome from visit number 1 of subject 764285508 (MER-FS) (*)[D]
Human Stool microbiome from visit number 1 of subject 764325968 (MER-FS) (*)[D]
Human Stool microbiome from visit number 1 of subject 764447348 (MER-FS) (*)[D]
Human Stool microbiome from visit number 1 of subject 764487809 (MER-FS) (*)[D]
Human Stool microbiome from visit number 1 of subject 764508039 (MER-FS) (*)[D]
Human Stool microbiome from visit number 1 of subject 764588959 (MER-FS) (*)[D]
Human Stool microbiome from visit number 1 of subject 764649650 (MER-FS) (*)[D]
Human Stool microbiome from visit number 1 of subject 764669880 (MER-FS) (*)[D]
Human Stool microbiome from visit number 1 of subject 765013792 (MER-FS) (*)[D]
Human Stool microbiome from visit number 1 of subject 765034022 (MER-FS) (*)[D]
Human Stool microbiome from visit number 1 of subject 765074482 (MER-FS) (*)[D]
Human Stool microbiome from visit number 1 of subject 765094712 (MER-FS) (*)[D]
Human Stool microbiome from visit number 1 of subject 765135172 (MER-FS) (*)[D]
Human Stool microbiome from visit number 1 of subject 765560005 (MER-FS) (*)[D]
Human Stool microbiome from visit number 1 of subject 765620695 (MER-FS) (*)[D]
Human Stool microbiome from visit number 1 of subject 765640925 (MER-FS) (*)[D]
Human Stool microbiome from visit number 1 of subject 765701615 (MER-FS) (*)[D]
Human Stool microbiome from visit number 1 of subject 809635352 (MER-FS) (*)[D]
Human Stool microbiome from visit number 1 of subject 823052294 (MER-FS) (*)[D]
Human Stool microbiome from visit number 1 of subject 861967750 (MER-FS) (*)[D]
Human Stool microbiome from visit number 2 of subject 158256496 (MER-FS) (*)[D]
Human Stool microbiome from visit number 2 of subject 158337416 (MER-FS) (*)[D]
Human Stool microbiome from visit number 2 of subject 158479027 (MER-FS) (*)[D]
Human Stool microbiome from visit number 2 of subject 158499257 (MER-FS) (*)[D]
Human Stool microbiome from visit number 2 of subject 158802708 (MER-FS) (*)[D]
Human Stool microbiome from visit number 2 of subject 158883629 (MER-FS) (*)[D]
Human Stool microbiome from visit number 2 of subject 158924089 (MER-FS) (*)[D]
Human Stool microbiome from visit number 2 of subject 158944319 (MER-FS) (*)[D]
Human Stool microbiome from visit number 2 of subject 159005010 (MER-FS) (*)[D]
Human Stool microbiome from visit number 2 of subject 159207311 (MER-FS) (*)[D]
Human Stool microbiome from visit number 2 of subject 159227541 (MER-FS) (*)[D]
Human Stool microbiome from visit number 2 of subject 159247771 (MER-FS) (*)[D]
Human Stool microbiome from visit number 2 of subject 159268001 (MER-FS) (*)[D]
Human Stool microbiome from visit number 2 of subject 159369152 (MER-FS) (*)[D]
Human Stool microbiome from visit number 2 of subject 159490532 (MER-FS) (*)[D]
Human Stool microbiome from visit number 2 of subject 159510762 (MER-FS) (*)[D]
Human Stool microbiome from visit number 2 of subject 159551223 (MER-FS) (*)[D]
Human Stool microbiome from visit number 2 of subject 159571453 (MER-FS) (*)[D]
Human Stool microbiome from visit number 2 of subject 159591683 (MER-FS) (*)[D]
Human Stool microbiome from visit number 2 of subject 159611913 (MER-FS) (*)[D]
Human Stool microbiome from visit number 2 of subject 159713063 (MER-FS) (*)[D]

Human Stool microbiome from visit number 2 of subject 159753524 (MER-FS) (*)[D]

Human Stool microbiome from visit number 2 of subject 159814214 (MER-FS) (*)[D]

Human Stool microbiome from visit number 2 of subject 160158126 (MER-FS) (*)[D]

Human Stool microbiome from visit number 2 of subject 246515023 (MER-FS) (*)[D]

Human Stool microbiome from visit number 2 of subject 370425937 (MER-FS) (*)[D]

Human Stool microbiome from visit number 2 of subject 604812005 (MER-FS) (*)[D]

Human Stool microbiome from visit number 2 of subject 638754422 (MER-FS) (*)[D]

Human Stool microbiome from visit number 2 of subject 686765762 (MER-FS) (*)[D]

Human Stool microbiome from visit number 2 of subject 763435843 (MER-FS) (*)[D]

Human Stool microbiome from visit number 2 of subject 763496533 (MER-FS) (*)[D]

Human Stool microbiome from visit number 2 of subject 763536994 (MER-FS) (*)[D]

Human Stool microbiome from visit number 2 of subject 763577454 (MER-FS) (*)[D]

Human Stool microbiome from visit number 2 of subject 763597684 (MER-FS) (*)[D]

Human Stool microbiome from visit number 2 of subject 763678604 (MER-FS) (*)[D]

Human Stool microbiome from visit number 2 of subject 763759525 (MER-FS) (*)[D]

Human Stool microbiome from visit number 2 of subject 763820215 (MER-FS) (*)[D]

Human Stool microbiome from visit number 2 of subject 763840445 (MER-FS) (*)[D]

Human Stool microbiome from visit number 2 of subject 763860675 (MER-FS) (*)[D]

Human Stool microbiome from visit number 2 of subject 763901136 (MER-FS) (*)[D]

Human Stool microbiome from visit number 2 of subject 763961826 (MER-FS) (*)[D]

Human Stool microbiome from visit number 2 of subject 763982056 (MER-FS) (*)[D]

Human Stool microbiome from visit number 2 of subject 764002286 (MER-FS) (*)[D]

Human Stool microbiome from visit number 2 of subject 764042746 (MER-FS) (*)[D]

Human Stool microbiome from visit number 2 of subject 764062976 (MER-FS) (*)[D]

Human Stool microbiome from visit number 2 of subject 764143897 (MER-FS) (*)[D]

Human Stool microbiome from visit number 2 of subject 764184357 (MER-FS) (*)[D]

Human Stool microbiome from visit number 2 of subject 764224817 (MER-FS) (*)[D]

Human Stool microbiome from visit number 2 of subject 764325968 (MER-FS) (*)[D]

Human Stool microbiome from visit number 2 of subject 764447348 (MER-FS) (*)[D]

Human Stool microbiome from visit number 2 of subject 764487809 (MER-FS) (*)[D]

Human Stool microbiome from visit number 2 of subject 764669880 (MER-FS) (*)[D]

Human Stool microbiome from visit number 2 of subject 764811490 (MER-FS) (*)[D]

Human Stool microbiome from visit number 2 of subject 764892411 (MER-FS) (*)[D]

Human Stool microbiome from visit number 2 of subject 765074482 replicate 1 (MER-FS) (*)[D]

Human Stool microbiome from visit number 2 of subject 765074482 replicate 2 (MER-FS) (*)[D]

Human Stool microbiome from visit number 2 of subject 765094712 (MER-FS) (*)[D]

Human Stool microbiome from visit number 2 of subject 809635352 (MER-FS) (*)[D]

Human Stool microbiome from visit number 3 of subject 159227541 (MER-FS) (*)[D]

Human Stool microbiome from visit number 3 of subject 159753524 (MER-FS) (*)[D]

Human Stool microbiome from visit number 3 of subject 763536994 (MER-FS) (*)[D]

Human Microbiome Production Phase community

Human Left Retroauricular crease microbiome from visit number 2 of subject 763961826 (MER-FS) (*)[D]

Human Left Retroauricular crease microbiome from visit number 2 of subject 764083206 (MER-FS) (*)[D]

Human Retroauricular crease microbiome from visit number 1 of subject 159450072 (MER-FS) (*)[D]

Human Retroauricular crease microbiome from visit number 1 of subject 159591683 replicate 2 (MER-FS) (*)[D]
Human Retroauricular crease microbiome from visit number 1 of subject 159591683 replicate1 (MER-FS) (*)[D]
Human Retroauricular crease microbiome from visit number 1 of subject 16040087 replicate 1 (MER-FS) (*)[D]
Human Retroauricular crease microbiome from visit number 1 of subject 16040088 replicate 2 (MER-FS) (*)[D]
Human Retroauricular crease microbiome from visit number 1 of subject 160603188 replicate 1 (MER-FS) (*)[D]
Human Retroauricular crease microbiome from visit number 1 of subject 160603188 replicate 2 (MER-FS) (*)[D]
Human Retroauricular crease microbiome from visit number 1 of subject 338793263 (MER-FS) (*)[D]
Human Retroauricular crease microbiome from visit number 1 of subject 404239096 (MER-FS) (*)[D]
Human Retroauricular crease microbiome from visit number 1 of subject 764042746 (MER-FS) (*)[D]
Human Retroauricular crease microbiome from visit number 2 of subject 159490532 (MER-FS) (*)[D]
Human Retroauricular crease microbiome from visit number 2 of subject 159571453 (MER-FS) (*)[D]
Human Retroauricular crease microbiome from visit number 2 of subject 159591683 replicate 1 (MER-FS) (*)[D]
Human Retroauricular crease microbiome from visit number 2 of subject 159591683 replicate 2 (MER-FS) (*)[D]
Human Retroauricular crease microbiome from visit number 2 of subject 15961191 (MER-FS) (*)[D]
Human Retroauricular crease microbiome from visit number 2 of subject 763496533 (MER-FS) (*)[D]
Human Retroauricular crease microbiome from visit number 2 of subject 764224817 (MER-FS) (*)[D]
Human Retroauricular crease microbiome from visit number 2 of subject 764305738 (MER-FS) (*)[D]
Human Retroauricular crease microbiome from visit number 2 of subject 764447348 (MER-FS) (*)[D]
Human Retroauricular crease microbiome from visit number 2 of subject 765094712 (MER-FS) (*)[D]

Human Right Retroauricular crease microbiome from visit number 2 of subject 15961191 replicate 2 (MER-FS) (*)[D]
Human Right Retroauricular crease microbiome from visit number 2 of subject 763961826 replicate 1 (MER-FS) (*)[D]
Human Right Retroauricular crease microbiome from visit number 2 of subject 763961826 replicate 2 (MER-FS) (*)[D]
Human Right Retroauricular crease microbiome from visit number 2 of subject 764083206 replicate 1 (MER-FS) (*)[D]
Human Right Retroauricular crease microbiome from visit number 2 of subject 764083206 replicate 2 (MER-FS) (*)[D]

Human Microbiome Production Phase community
Human Mid vagina microbiome from visit number 1 of subject 763577454 (MER-FS) (*)[D]
Human Mid vagina microbiome from visit number 2 of subject 763577454 (MER-FS) (*)[D]

Human Microbiome Production Phase community
Human Posterior fornix microbiome from visit number 1 of subject 158337416 (MER-FS) (*)[D]
Human Posterior fornix microbiome from visit number 1 of subject 158458797 (MER-FS) (*)[D]
Human Posterior fornix microbiome from visit number 1 of subject 158742018 (MER-FS) (*)[D]
Human Posterior fornix microbiome from visit number 1 of subject 158944319 (MER-FS) (*)[D]
Human Posterior fornix microbiome from visit number 1 of subject 159227541 (MER-FS) (*)[D]
Human Posterior fornix microbiome from visit number 1 of subject 159247771 (MER-FS) (*)[D]
Human Posterior fornix microbiome from visit number 1 of subject 159753524 replicate 1 (MER-FS) (*)[D]
Human Posterior fornix microbiome from visit number 1 of subject 159753524 replicate 2 (MER-FS) (*)[D]
Human Posterior fornix microbiome from visit number 1 of subject 159915365 (MER-FS) (*)[D]
Human Posterior fornix microbiome from visit number 1 of subject 160319967 (MER-FS) (*)[D]
Human Posterior fornix microbiome from visit number 1 of subject 160502038 (MER-FS) (*)[D]
Human Posterior fornix microbiome from visit number 1 of subject 160582958 (MER-FS) (*)[D]
Human Posterior fornix microbiome from visit number 1 of subject 160643649 (MER-FS) (*)[D]
Human Posterior fornix microbiome from visit number 1 of subject 160704339 (MER-FS) (*)[D]

Human Posterior fornix microbiome from visit number 1 of subject 246515023 (MER-FS) (*)[D]
Human Posterior fornix microbiome from visit number 1 of subject 338793263 (MER-FS) (*)[D]
Human Posterior fornix microbiome from visit number 1 of subject 370425937 (MER-FS) (*)[D]
Human Posterior fornix microbiome from visit number 1 of subject 508703490 (MER-FS) (*)[D]
Human Posterior fornix microbiome from visit number 1 of subject 550534656 (MER-FS) (*)[D]
Human Posterior fornix microbiome from visit number 1 of subject 638754422 (MER-FS) (*)[D]
Human Posterior fornix microbiome from visit number 1 of subject 675950834 (MER-FS) (*)[D]
Human Posterior fornix microbiome from visit number 1 of subject 706846339 (MER-FS) (*)[D]
Human Posterior fornix microbiome from visit number 1 of subject 737052003 (MER-FS) (*)[D]
Human Posterior fornix microbiome from visit number 1 of subject 763577454 (MER-FS) (*)[D]
Human Posterior fornix microbiome from visit number 1 of subject 763759525 (MER-FS) (*)[D]
Human Posterior fornix microbiome from visit number 1 of subject 763820215 (MER-FS) (*)[D]
Human Posterior fornix microbiome from visit number 1 of subject 763840445 (MER-FS) (*)[D]
Human Posterior fornix microbiome from visit number 1 of subject 763901136 (MER-FS) (*)[D]
Human Posterior fornix microbiome from visit number 1 of subject 764042746 (MER-FS) (*)[D]
Human Posterior fornix microbiome from visit number 1 of subject 764143897 (MER-FS) (*)[D]
Human Posterior fornix microbiome from visit number 1 of subject 764588959 (MER-FS) (*)[D]
Human Posterior fornix microbiome from visit number 1 of subject 764649650 (MER-FS) (*)[D]
Human Posterior fornix microbiome from visit number 1 of subject 765013792 (MER-FS) (*)[D]
Human Posterior fornix microbiome from visit number 1 of subject 765337473 (MER-FS) (*)[D]
Human Posterior fornix microbiome from visit number 1 of subject 765620695 (MER-FS) (*)[D]
Human Posterior fornix microbiome from visit number 1 of subject 765640925 (MER-FS) (*)[D]
Human Posterior fornix microbiome from visit number 1 of subject 809635352 (MER-FS) (*)[D]
Human Posterior fornix microbiome from visit number 2 of subject 158256496 (MER-FS) (*)[D]
Human Posterior fornix microbiome from visit number 2 of subject 158337416 (MER-FS) (*)[D]
Human Posterior fornix microbiome from visit number 2 of subject 158883629 (MER-FS) (*)[D]
Human Posterior fornix microbiome from visit number 2 of subject 158944319 (MER-FS) (*)[D]
Human Posterior fornix microbiome from visit number 2 of subject 159005010 (MER-FS) (*)[D]
Human Posterior fornix microbiome from visit number 2 of subject 159227541 (MER-FS) (*)[D]
Human Posterior fornix microbiome from visit number 2 of subject 159247771 (MER-FS) (*)[D]
Human Posterior fornix microbiome from visit number 2 of subject 159713063 (MER-FS) (*)[D]
Human Posterior fornix microbiome from visit number 2 of subject 159753524 (MER-FS) (*)[D]
Human Posterior fornix microbiome from visit number 2 of subject 246515023 (MER-FS) (*)[D]
Human Posterior fornix microbiome from visit number 2 of subject 370425937 (MER-FS) (*)[D]
Human Posterior fornix microbiome from visit number 2 of subject 763577454 (MER-FS) (*)[D]
Human Posterior fornix microbiome from visit number 2 of subject 763759525 (MER-FS) (*)[D]
Human Posterior fornix microbiome from visit number 2 of subject 763820215 (MER-FS) (*)[D]
Human Posterior fornix microbiome from visit number 2 of subject 763840445 (MER-FS) (*)[D]
Human Posterior fornix microbiome from visit number 2 of subject 763901136 (MER-FS) (*)[D]
Human Posterior fornix microbiome from visit number 2 of subject 764042746 (MER-FS) (*)[D]
Human Posterior fornix microbiome from visit number 2 of subject 764062976 (MER-FS) (*)[D]
Human Posterior fornix microbiome from visit number 2 of subject 764143897 (MER-FS) (*)[D]
Human Posterior fornix microbiome from visit number 2 of subject 764811490 (MER-FS) (*)[D]
Human Posterior fornix microbiome from visit number 2 of subject 809635352 (MER-FS) (*)[D]
Human Posterior fornix microbiome from visit number 3 of subject 158883629 (MER-FS) (*)[D]
Human Posterior fornix microbiome from visit number 3 of subject 159753524 (MER-FS) (*)[D]

Human Microbiome Production Phase community

Human Vaginal introitus microbiome from visit number 1 of subject 763577454 (MER-FS) (*)[D]

Human Vaginal introitus microbiome from visit number 2 of subject 763577454 (MER-FS) (*)[D]

Human Vaginal introitus microbiome from visit number 2 of subject 764042746 (MER-FS) (*)[D]
