# Supplementary material for: Functional Environmental Screening of a Metagenomic Library Identifies stlA; A Unique Salt Tolerance Locus from the Human Gut Microbiome
Source: PLoS One. 2013 Dec 12;8(12):e82985. doi: 10.1371/journal.pone.0082985 (PMC3861447; doi:10.1371/journal.pone.0082985)
Supplement: Table S1 — Bacterial strains, plasmids and transposon used in this study. (PDF) [file pone.0082985.s007.pdf]

Table S1. Strains, plasmids and transposon used in this study.

Strain, plasmid or transposon	Genotype or characteristic(s)	Source or reference
**Strains**		
E. coli EPI300	F$^-$ *mcrA*Δ(*mrr-hsd*RMS-*mcrBC*) Φ80d*lacZ*ΔM15 Δ*lac*X74 *recA*1 *endA*1 *araD*139 Δ(*ara, leu*)7697 *galU galK* λ$^-$ *rpsL nupG trfA dhfr*; high-transformation efficiency of large DNA	Epicentre Biotechnologies, Madison, WI, USA
E. coli MKH13	MC4100*Δ*(*putPA*)101D(*proP*)2D(*proU*)	[22]
E. coli MKH13::pCI372	MKH13 containing pCI372 shuttle vector	This study
E. coli MKH13::pCI372-*stlA*	MKH13 containing pCI372 with *stlA* gene from human gut metagenomic library clone SMG 25	This study
L. lactis subspecies *cremoris* MG1363	Plasmid-free *Lactococcus* strain	[23]
L. lactis MG1363::pCI372	MG1363 containing pCI372 shuttle vector	This study
L. lactis MG1363::pCI372-*stlA*	MG1363 containing pCI372 with *stlA* gene from human gut metagenomic library clone SMG 25	This study
**Plasmids**		
pCI372	Shuttle vector between *E. coli* and *L. lactis*, Cm^R	[24]
pCC1FOS	Fosmid cloning vector, Cm^R	Epicentre Biotechnologies, Madison, WI, USA
**Transposon**		
EZ-Tn*5* <*ori*V/ KAN-2>	Hyperactive Tn*5* transposon, Kan^R, inducible high copy origin of replication – *ori*V	Epicentre Biotechnologies, Madison, WI, USA

Cm^R = chloramphenicol resistance; Kan^R = kanamycin resistance.
