# Supplementary material for: Functional Environmental Screening of a Metagenomic Library Identifies stlA; A Unique Salt Tolerance Locus from the Human Gut Microbiome
Source: PLoS One. 2013 Dec 12;8(12):e82985. doi: 10.1371/journal.pone.0082985 (PMC3861447; doi:10.1371/journal.pone.0082985)
Supplement: Table S2 — Primers used in this study. (PDF) [file pone.0082985.s008.pdf]

Table S2. Primers used in this study.

Primer	Sequence (5' – 3')[a]
pCI372 FP	CGGGAAGCTAGAGTAAGTAG
pCI372 RP	CCTCTCGGTTATGAGTTAG
stlA FP	AAAACTGCAGTTCTGGCAGCAGTGATTTTG
stlA RP	GCTCTAGACGGTCGAGCAAGGTAATAGG
stlA-J FP	TGCTCTTCCGAAGCAGTCAG
stlA-J RP	AGCATATCGAAGACGGCCAG
stlA-OUT FP	CTGCTCTGTTGATGGGGTTT
stlA-OUT RP	CGGGCAACTACAAGGATGAT
stlA-IN FP	TATGGGAGGGGCTACTACGG
stlA-IN RP	ACCCAGTTGCCAAGCATATC
EZ-Tn FP-1	GCCAACGACTACGCACTAGCCAAC
EZ-Tn RP-1	GAGCCAATATGCGAGAACACCCGAGAA

[a]Restriction enzyme recognition sequences are underlined; FP= forward primer; RP= reverse primer.
